# Faradaic Electrodes Open a New Era for Capacitive Deionization

**DOI:** 10.1002/advs.202002213

**Published:** 2020-10-11

**Authors:** Qian Li, Yun Zheng, Dengji Xiao, Tyler Or, Rui Gao, Zhaoqiang Li, Ming Feng, Lingling Shui, Guofu Zhou, Xin Wang, Zhongwei Chen

**Affiliations:** ^1^ South China Academy of Advanced Optoelectronics and International Academy of Optoelectronics at Zhaoqing South China Normal University Guangdong 510631 P. R. China; ^2^ Department of Chemical Engineering Waterloo Institute of Nanotechnology University of Waterloo 200 University Ave West Waterloo Ontario N2L 3G1 Canada; ^3^ Key Laboratory of Functional Materials Physics and Chemistry of the Ministry of Education Jilin Normal University Changchun 130103 P. R. China

**Keywords:** capacitive deionization, desalination, Faradaic electrodes, ion capture mechanisms

## Abstract

Capacitive deionization (CDI) is an emerging desalination technology for effective removal of ionic species from aqueous solutions. Compared to conventional CDI, which is based on carbon electrodes and struggles with high salinity streams due to a limited salt removal capacity by ion electrosorption and excessive co‐ion expulsion, the emerging Faradaic electrodes provide unique opportunities to upgrade the CDI performance, i.e., achieving much higher salt removal capacities and energy‐efficient desalination for high salinity streams, due to the Faradaic reaction for ion capture. This article presents a comprehensive overview on the current developments of Faradaic electrode materials for CDI. Here, the fundamentals of Faradaic electrode‐based CDI are first introduced in detail, including novel CDI cell architectures, key CDI performance metrics, ion capture mechanisms, and the design principles of Faradaic electrode materials. Three main categories of Faradaic electrode materials are summarized and discussed regarding their crystal structure, physicochemical characteristics, and desalination performance. In particular, the ion capture mechanisms in Faradaic electrode materials are highlighted to obtain a better understanding of the CDI process. Moreover, novel tailored applications, including selective ion removal and contaminant removal, are specifically introduced. Finally, the remaining challenges and research directions are also outlined to provide guidelines for future research.

## Graphical abstract

Faradaic electrodes open a new era for capacitive deionization (CDI) by providing enhanced salt removal capacity and energy‐efficient desalination for high salinity streams. Advances in the development of Faradaic electrodes for CDI are comprehensively summarized, spanning from cell architectures, key performance metrics, advanced materials, and related ion capture mechanisms, to tailored applications as well as main challenges and perspectives.

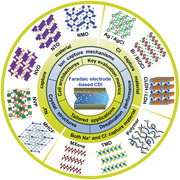

## 1 Introduction

### 1.1 Water Scarcity and Main Desalination Strategies

**Figure 1:**
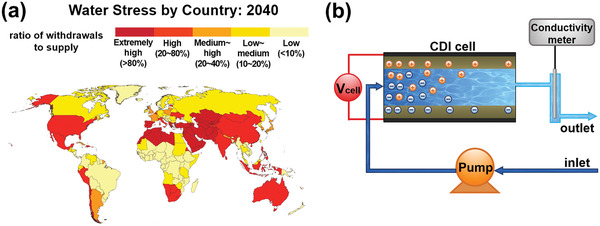
a) Water stress by country: 2040. Reproduced with permission.^[^ ^1^ ^]^ Copyright 2015, World Resources Institute. b) Schematic diagram of CDI setup, including CDI cell, peristaltic pump, raw inlet water, direct‐current power supply, and conductivity meter.

Water scarcity has become one of the most concerning global challenges of our time. Currently, 17 countries face extremely high levels of water stress, where on average ≥ 80% of their available supply is consumed each year, and by 2040, this number could rise to 33 (**Figure** **1**a).^[^ ^1^, ^2^ ^]^ Such a narrow gap between supply and demand can place severe stress on nations and societies, potentially impacting the economic vitality, national security, public health, and ecosystem balance. However, the challenge of providing adequate and clean drinking water is further complicated by population expansion, industrialization, water pollution, and climate change.^[^ ^2^ ^]^ Therefore, it is very urgent to develop strategies to mitigate water scarcity. Although some approaches, such as water reuse, water conservation, and developed catchment and distribution systems can be adopted to alleviate the stresses on water supply to some extent, they are still far from satisfactory to meet the dramatically growing demand of fresh water.^[^ ^2^ ^]^

Given that over 97% of water on earth is saline,^[^ ^2^ ^]^ desalination of seawater or brackish water is a possible way to ensure a sustainable supply of freshwater.^[^ ^3^ ^]^ Technologies for desalination have developed rapidly in recent years, which mainly include reverse osmosis (RO) and electrodialysis (ED) based on membrane separation, and multistage flash (MSF) and multieffect distillation (MED) based on a phase change thermal process.^[^ ^4^ ^]^ These technologies have been adopted by some water‐stressed countries, especially those in the Middle East such as Saudi Arabia, where over 70% of its fresh water comes from desalination. However, most of these traditional desalination technologies are highly energy intensive.^[^ ^2^, ^3^ ^]^ RO must be driven by high osmotic pressure (1–10 MPa) for salt separation and suffer from membrane fouling.^[^ ^5^, ^6^ ^]^ Thermal desalination processes require large amounts of heat energy to separate water by vaporization and has constant issues with the corrosion of facility equipment.^[^ ^2^, ^3^ ^]^ ED requires extremely high voltages (>20 V) to force ions to move in a directional manner to realize separation, which may result in water decomposition.^[^ ^3^, ^7^ ^]^ Therefore, the search for new alternative desalination technologies with low energy consumption, high efficiency, and devoid of pollution is urgently needed.

Capacitive deionization (CDI) is burgeoning as a desalination technology in recent years due to it being an energy‐efficient, cost effective, and environmentally‐friendly process.^[^ ^8^, ^9^ ^]^ Unlike traditional desalination technologies, which need to be equipped with sophisticated membrane elements, high pressure pumps or thermal sources, CDI system could be operated under low (sub‐osmotic) pressures and room temperatures with low applied cell voltage (<2 V), making it easier to achieve scaling. Moreover, the CDI process remove the minority salt ions rather than the majority water from the saline solution, making CDI suitable for energy efficient desalination of low salinity streams, such as brackish water that generally contain total dissolved salts (TDS) in the range of 1–10 g L^−1^ (the TDS of seawater is 35 g L^−1^).^[^ ^3^, ^10^ ^]^ For desalination of brackish water, the energy consumption of CDI is only 0.13–0.59 kWh m^−3^,^[^ ^11^ ^]^ which is much lower than that of RO (3.5–4.5 kWh m^−3^),^[^ ^3^ ^]^ the most energy efficient traditional desalination technology.

### 1.2 Carbon Electrodes for CDI

The schematic diagram of CDI setup is shown in Figure 1b, where the conventional CDI cell are composed of a pair of parallel porous carbon electrodes, with or without a porous electric insulating separator in‐between. When applying a voltage difference (generally 1.0–1.5 V) on the two electrodes, dissolved salt ions migrate into electric double layers (EDLs) on the porous surface of the oppositely charged electrode respectively (a process called as electrosorption), realizing the removal of salt from the feed water. When the external voltage is reversed or removed, the electrodes can achieve regeneration by releasing the captured ions back into the solution.^[^ ^8^, ^12^ ^]^ Among all these components in CDI cells, electrodes play the most critical role in capturing salt ions.

Carbon materials have been widely employed as electrode for CDI cells, such as activated carbon (AC),^[^ ^13^ ^]^ carbon nanotubes,^[^ ^14^ ^]^ graphene,^[^ ^15^ ^]^ mesoporous carbon,^[^ ^16^ ^]^ carbon frameworks,^[^ ^17^ ^]^ and carbon aerogel,^[^ ^18^ ^]^ which have obvious merits, such as abundant resources, easy to produce, good electrical conductivity, and tunable porous structure. Meanwhile, the CDI consisting of carbon electrodes has made great development in cell design,^[^ ^19^, ^20^, ^21^ ^]^ pore structure optimization,^[^ ^22^ ^]^ and modification of Donnan model for exploring the ion electrosorption process,^[^ ^23^, ^24^ ^]^ which leads several effective improvements in desalination performance.

However, as capacitive carbon electrodes are based on ion electrosorption mechanism which strongly depends on the effective specific surface area, the salt removal capacities of the corresponding CDI cells are generally limited to 20 mg g^−1^. Besides, unavoidable co‐ion expulsion has always been parasitic in the process of capturing counterions from feedwater, and is especially severe in high ionic strength feedwaters, leading to a significant decrease in charge efficiency and raised energy consumption.^[^ ^25^, ^26^ ^]^ Moreover, during continuous charge–discharge cycles, the oxidation of anodic carbon electrodes will lead to deterioration of structural properties of the electrodes and further rapid capacity fading, especially when feedwaters contain dissolved oxygen at typical levels of natural surface water (5–10 mg L^−1^).^[^ ^8^, ^27^, ^28^ ^]^ Adding permselective ion‐exchange membranes (IEMs) to separate electrodes from the saline water can effectively ameliorate the above‐mentioned problems.^[^ ^21^, ^29^, ^30^ ^]^ Depending on their permselectivity, IEMs can block co‐ion expulsion, resulting in an enhancement in salt removal capacity and charge efficiency.^[^ ^26^, ^30^ ^]^ IEMs can also effectively inhibit the oxidation of anodic carbon electrodes, thereby improving the stability of CDI long‐term desalination.^[^ ^31^ ^]^ The development of flow‐electrode CDI can even enable continuous desalination.^[^ ^32^, ^33^ ^]^ Although various strategies have been developed for CDI with carbon electrodes,^[^ ^16^, ^30^, ^32^ ^]^ inherent performance limitations of carbon electrodes based on ion electrosorption still remain. Therefore, this has motivated research into electrode materials with new ion capture mechanisms.

### 1.3 Faradaic Electrodes for CDI

Inspired by the materials used in highly developed electrochemical energy storage fields such as batteries^[^ ^34^, ^35^ ^]^ and pseudocapacitors,^[^ ^36^, ^37^ ^]^ which store ions by Faradaic reactions (hence termed as Faradaic electrodes) rather than electrosorption, Faradaic electrode materials have generated interest in the CDI community. This was first demonstrated in 1960 by Blair and Murphy^[^ ^38^ ^]^ who paired a chemically modified carbon electrode with Ag/AgCl for electrochemical deionization. Since then, there was no significant progress in exploring Faradaic materials for desalination until 2012 when Pasta et al.^[^ ^39^ ^]^ proposed the concept of a “desalination battery” employing two Faradaic electrodes for desalination, in which a sodium manganese oxide (Na_2−_ *_x_*Mn_5_O_10_) cathode captures Na^+^ by insertion while a Ag anode captures Cl^−^ via conversion reaction to AgCl. Unlike carbon, Faradaic materials capture ions by Faradaic reactions involving the charge transfer between electrodes and the ions in solution. Such charge transfer can be accomplished by ion insertion within crystal structures,^[^ ^40^, ^41^ ^]^ conversion reaction with forming new compounds,^[^ ^42^, ^43^ ^]^ or ion‐redox active moiety interaction.^[^ ^44^, ^45^ ^]^ This endows Faradaic materials with three advantages over carbon as CDI electrode. First, Faradaic reactions within the bulk of electrodes allow for higher desalination capacity, which can be as high as >100 mg g^−1^. Second, Faradaic electrodes with permselectivity are not affected by significant co‐ion expulsion, thus making it possible to desalinate higher salinity feedwaters such as seawater. These two advantages bring Faradaic electrodes the third advantage, that is, lower energy consumption for desalination, which is especially significant at high ionic strength streams with reduced ionic resistance for ion migration. As a consequence, the exploration of Faradaic electrodes for enhanced desalination performance has attracted considerable interest. With the growing of paper numbers, the desalination performance level also increases.

With respect to the specific development of Faradaic electrode materials, in 2014, Lee et al.^[^ ^46^ ^]^ proposed a concept similar to Blair and Murphy's earlier, named “hybrid CDI”, which used Na_4_Mn_9_O_18_ as the cathode and AC as the anode with an anion‐exchange membrane (AEM), exhibiting a high desalination capacity of 31.2 mg g^−1^. Later, a new Cl^−^ capture material was introduced by Nam et al.^[^ ^46^ ^]^ and Chen et al.,^[^ ^47^ ^]^ where Bi anode was employed to capture Cl^−^ by oxidation to BiOCl (a reversible conversion reaction, Bi/BiOCl). From the perspective of avoiding the issue of Cl^−^ capture, some researchers proposed novel CDI cells employing Na^+^ capture materials for both electrodes with an AEM in‐between.^[^ ^48^, ^49^ ^]^ Corresponding proof‐of‐concept cells with metal hexacyanoferrates as electrode have achieved considerable capacity with a range of 30–100 mg g^−1^.^[^ ^50^, ^51^, ^52^, ^53^ ^]^ Apart from exclusive Na^+^/Cl^−^ capture materials, some materials have emerged for both Na^+^ and Cl^−^ capture, such as MXenes^[^ ^54^ ^]^ and transition metal dichalcogenides^[^ ^55^ ^]^ consisting of 2D layers (with large interlayer distance) relying on the mechanism of ion insertion. In addition, redox‐active polymers are also promising for water desalination, as they can show either strong interactions with Na^+^ (such as redox‐active polyimide)^[^ ^56^ ^]^ or Cl^−^ (such as polymers with [Fe(CN)_6_]^4−^)^[^ ^44^ ^]^ depending on the tunable redox active moieties. In addition to the aforementioned novel ion‐selective Na^+^/Cl^−^ capture electrode materials^[^ ^47^, ^57^, ^58^ ^]^ and various cell designs,^[^ ^43^, ^46^, ^49^ ^]^ some recent promising developments of Faradaic electrodes have been achieved, such as research on the influences of operational parameters on CDI performance metrics,^[^ ^41^, ^43^, ^59^ ^]^ and some typical scientific and practical application.^[^ ^40^, ^60^, ^61^, ^62^, ^63^ ^]^ However, it appears that Faradaic electrodes used in CDI cells are still not mature enough to meet the requirement for practical implementation and commercialization, which can be attributed to major challenges such as not fully understanding the ion capture mechanisms and behaviors of materials, matching issues between the Na^+^ capture cathode and Cl^−^ capture anode, and the need to establish standardized test conditions, etc.

**Figure 2:**
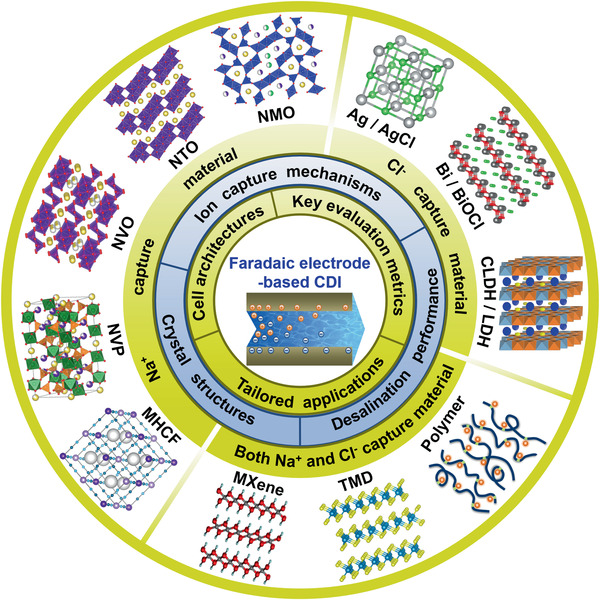
Overview for the fundamentals, advanced materials and applications of CDI with Faradaic electrodes.

To facilitate research and development in overcoming these challenges, we present this comprehensive review to specifically focused on Faradaic electrode material‐based CDI, which is rarely summarized in current published review papers.^[^ ^64^, ^65^, ^66^, ^67^, ^68^ ^]^ As outlined in **Figure** **2**, the key topics discussed in this paper span from the fundamentals and novel ion capture electrode materials to tailored applications. In particular, insights into ion capture mechanisms will be highlighted to provide a better understanding of the desalination process. Furthermore, the outstanding challenges and possible research directions will be summarized and proposed. We believe that Faradaic electrode material‐based CDI cells are highly promising for widespread application in water desalination with further research and development.

## 2 Fundamentals of Faradaic Electrode Materials in CDI

To provide a fundamental understanding of CDI and the working mechanism of Faradaic electrodes in CDI, we introduce the current CDI cell architectures based on Faradaic electrodes, the key evaluation metrics for CDI performance along with corresponding affecting factors, and the ion capture mechanisms of Faradaic electrode materials as well as the basic requirements for high performance Faradaic electrode materials. More details can be seen as follows.

### 2.1 CDI Cell Architectures

#### 2.1.1 CDI Cell Architectures

**Figure 3:**
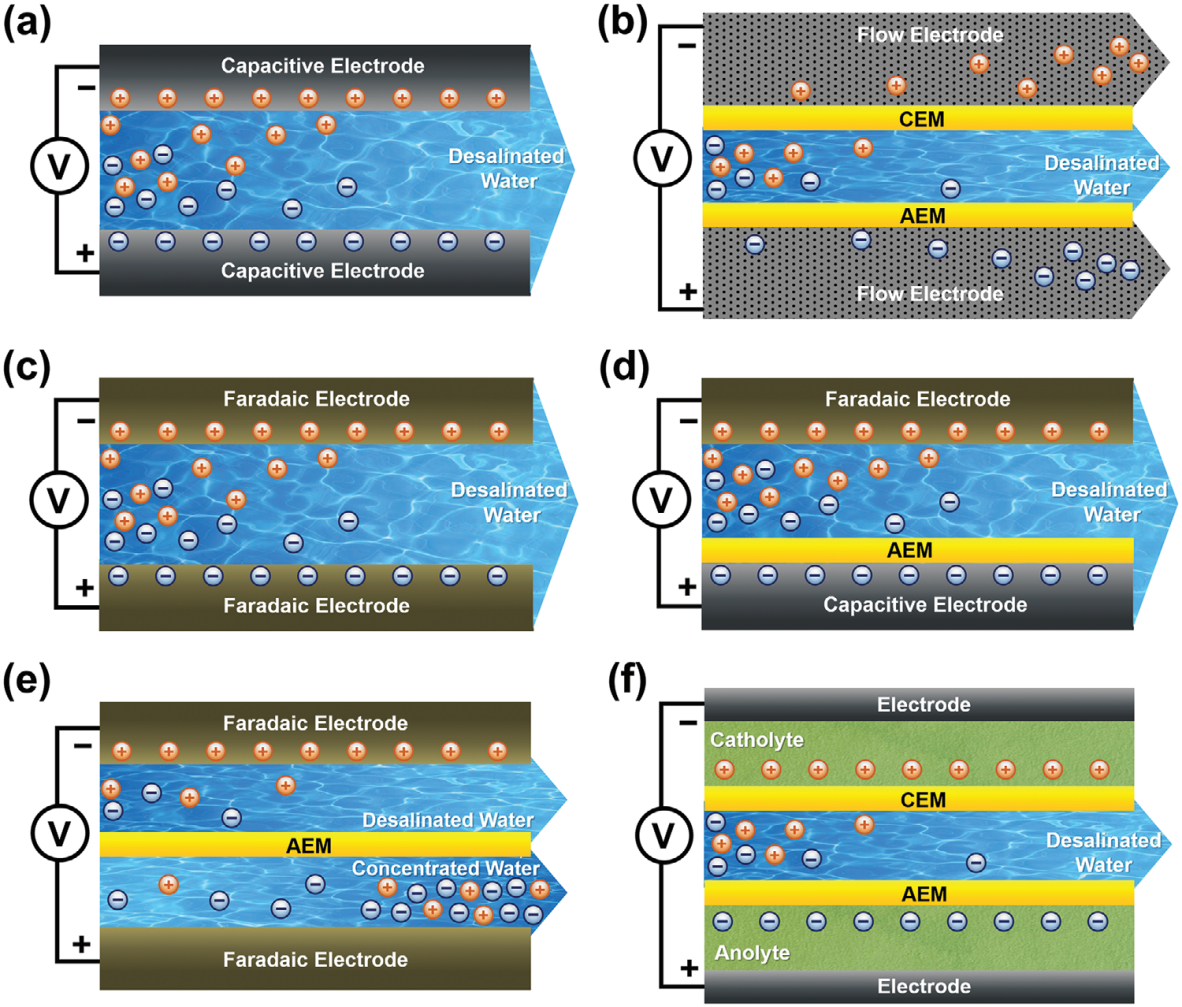
Schematic diagram of desalination with different CDI architectures: a) conventional CDI, b) flow‐electrode CDI, c) Faradaic CDI, d) hybrid CDI, e) sodium‐ion desalination (NID), and f) CDI with redox electrolyte. In these CDI cell architectures, all electrodes, whether carbon materials or Faradaic materials, can be paired with an IEM.

We classify the CDI cell architectures according to the type of electrode material (i.e., carbon or Faradaic material) and their targeted ions for removal (i.e., cation or anion), as shown in **Figure** **3**. In these CDI cell architectures, all electrodes, whether carbon materials or Faradaic materials, can be paired with an IEM.

The conventional CDI (Figure 3a) is the original CDI cell architecture dating back to the late 1960s,^[^ ^69^ ^]^ which is based on the electrosorption of ions on the surface of a pair of oppositely‐charged electrodes that typically comprise highly porous carbons. This CDI architecture is typically used to treat low salinity streams of less than 3 g L^−1^.^[^ ^70^ ^]^ One important improvement of the conventional CDI cell architecture is the introduction of IEMs in front of the electrodes, which was termed membrane CDI (MCDI) in 2006 by Lee et al.^[^ ^21^ ^]^ Due to the permselectivity of IEMs, co‐ion expulsion can be effectively suppressed, leading to increased flux of counterions to reach electroneutrality, with the end result that fewer ions remain in the stream and the desalination performance is significantly improved.^[^ ^26^, ^30^ ^]^ Some studies have explored the possibility of MCDI for high salinity streams,^[^ ^29^, ^30^, ^71^ ^]^ such as applying a reversed‐voltage during discharge step, resulting in a salt removal capacity of 26 mg g^−1^ in 600 × 10^−3^ m NaCl solution.^[^ ^30^ ^]^ Another example using an over‐potential (2.4 V) obtained a value up to 64.7 mg g^−1^ in 500 × 10^−3^ m NaCl solution.^[^ ^29^ ^]^ IEMs has also been demonstrated as effective in prolonging the lifetime of electrodes by alleviating oxidation of anodic carbon electrodes.^[^ ^31^, ^66^ ^]^ In addition to CDI with static electrodes, the development of flow‐electrode CDI (Figure 3b) even can enable continuous desalination.^[^ ^32^, ^33^ ^]^ The flow electrodes consist of a suspension of carbon particles, which can be pumped through the electrode compartments. The regeneration of flow electrodes can occur as a separate process by mixing^[^ ^72^ ^]^ or in a separate module,^[^ ^19^ ^]^ enabling continuous desalination. By continuously replenishing uncharged carbon suspensions into the cell, the CDI cell can effectively improve the capacitance available for desalination, and thus allow for the treatment of higher salinity streams compared to static CDI cells.^[^ ^19^, ^32^ ^]^ So far, various continuous flow‐electrode CDI configurations have been developed, such as a single‐module configuration,^[^ ^73^ ^]^ a two‐module configuration,^[^ ^74^ ^]^ and a two‐step regeneration configuration with energy recovery.^[^ ^75^ ^]^

On the other hand, CDI architectures utilizing Faradaic electrode materials are an emerging technology that have recently been investigated. Faradaic CDI employs two Faradaic electrodes for salt water desalination, where the two Faradaic electrodes are usually in different types and react exclusively with either the cations or anions (Figure 3c). The most common collocation of Faradaic CDI is to employ a Na^+^ insertion material, such as a sodium transition metal oxide, polyanionic‐type compound, or metal hexacyanometalate, to capture/release Na^+^ via insertion/extraction, while the other electrode that captures Cl^−^ is a conversion‐type material such as Ag/AgCl or Bi/BiOCl.^[^ ^39^, ^47^ ^]^ Since the first scientific demonstration of Faradaic CDI (Na_2_Mn_5_O_10_//AgCl CDI cell) from Pasta et al. in 2012,^[^ ^39^ ^]^ Faradaic CDI cells have shown considerable potential with respect to the minimization of the co‐ion expulsion effect, the improvement of salt removal capacity, and the ability to treat high concentrations of salt solution such as brackish water or even seawater.^[^ ^39^, ^43^ ^]^ Apart from the common collocation using two different Faradaic electrodes, few subsequent Faradaic CDI cells have employed two identical Faradaic electrodes, known as symmetric Faradaic CDI. These electrodes typically are 2D intercalating nanomaterials, such as MXene or transition metal dichalcogenide, which can intercalate both cations and anions at the same time at two parallel cell poles respectively.^[^ ^55^, ^58^ ^]^ However, the applicability of symmetric Faradaic CDI cells is limited by its distinctly lower capacity for Cl^−^ removal compared to Na^+^, due to the intrinsically negative charge of these 2D intercalating host layers.^[^ ^54^, ^55^ ^]^

Hybrid CDI (HCDI) is a CDI architecture that is composed of one Faradaic electrode and one capacitive carbon electrode. The first HCDI system was investigated by Blair and Murphy in 1960.^[^ ^69^ ^]^ They utilized an Ag/AgCl electrode to remove Cl‐ via conversion reaction and tannin acid functionalized‐graphite electrode to remove Na^+^ via electrosorption. Currently, by far the most typical configuration for HCDI is to use a Faradaic electrode for Na^+^ insertion and a porous carbon electrode coupled with an AEM for selective Cl^−^ adsorption (Figure 3d), as reported by Lee et al.^[^ ^46^ ^]^ in 2014. Their HCDI (Na_4_Mn_9_O_18_//AC‐AEM) achieved a high desalination capacity of 31.2 mg g^−1^, more than twice that of a conventional CDI (13.5 mg g^−1^). HCDI is more practical than Faradaic CDI for large‐scale applications since there is no need to incorporate high‐cost Cl^−^ capture Faradaic materials such as the Ag‐based electrode. AEM is not mandatory feature required by HCDI. Some HCDI cells more recently reported still use a Na^+^ insertion Faradaic electrode (such as MnO_2_ and NaTi_2_(PO_4_)_3_) but adopt positively charged carbon electrodes instead of carbon‐AEM suit for selective Cl^−^ adsorption, which also generated good desalination results.^[^ ^76^, ^77^ ^]^ In contrast to the aforementioned familiar configuration for HCDI, a less common configuration inverts configuration by employing a Faradaic electrode for Cl^−^ capture and a capacitive carbon electrode for Na^+^ capture, such as Ag paired with carbon‐cation exchange membrane (CEM),^[^ ^78^ ^]^ or calcined layered double hydroxide matched with carbon electrode (see Section 3.2.3).^[^ ^57^, ^79^, ^80^ ^]^ However, this configuration remains poorly studied as identifying acceptable Cl^−^ storage electrode materials that can meet key criteria (low cost, insolubility, stability, and reversibility within a limited voltage range) remains a research challenge.

The sodium‐ion desalination (NID) can completely circumvent the issue with the lack of available Cl^−^ capture electrodes. As shown in Figure 3e, this architecture employs two cation‐selective Faradaic electrodes and two parallel flow channels separated by an AEM. In this desalination system, one electrode takes in Na^+^ from the feed solution in one flow channel, while the other electrode releases Na^+^ into solution in the opposite channel. At the same time, Cl^−^ migrate from the Na^+^ deficient channel to the Na^+^ enriched channel through the AEM, thus generating desalinated and concentrated effluents simultaneously. The reverse process occurs in these two channels when the external voltage is reversed, avoiding the two‐cycle mode needed for the other three aforementioned CDI architectures, while also achieving desalination during both the charge and discharge steps in a continuous approach. Smith et al.^[^ ^48^, ^49^ ^]^ first introduced this novel NID concept in 2016, and later reported the theoretical basis of a NID cell, demonstrating that the proposed cell has the capability of treating high salinity streams and that the AEM plays a critical role in desalination efficiency. To date, NID typically incorporate metal hexacyanometalates as Na^+^ insertion Faradaic electrodes to achieve salt removal. For instance, a symmetric NID cell with two identical nickel hexacyanoferrate (NiHCF) electrodes exhibited a salt removal capacity of 34 mg g^−1^.^[^ ^51^ ^]^ A symmetric NID cell using CuHCF//CuHCF electrodes^[^ ^52^ ^]^ and an asymmetric NID cell composed of two different electrodes (NaNiHCF//NaFeHCF)^[^ ^50^ ^]^ have been also reported with higher salt removal capacity even up to 100 mg g^−1^. In analogy to NID consisting of cation‐selective electrodes and an AEM, a niche CDI architecture invert the concept by using anion‐selective electrodes and a CEM, which had been previously described by Grygolowicz et al.^[^ ^81^ ^]^ and can be termed as chloride‐ion desalination (CID). The current CID always use two Ag/AgCl electrodes to capture Cl^−^.^[^ ^82^, ^83^ ^]^ Although such cells show good desalination capacity, the high cost of Ag undoubtedly limits their practicality, and hence CID has not been classified into the current main CDI architectures with Faradaic electrode materials.

The CDI architecture with redox electrolyte realizes ion removal through charge compensation which driven by the redox reactions of redox‐active ions (e.g., I^−^, Br^−^, [Fe(CN)_6_]^3−^) dissolved in electrolyte.^[^ ^84^, ^85^, ^86^, ^87^ ^]^ Figure 3f shows the schematic diagram of a bielectrolyte cell architecture, in which the feedwater stream in the middle channel is separated from the two redox electrolytes in the side channels by IEMs. When voltage is applied to the two electrodes, the dissolved redox‐active ions move toward the electrolyte‐electrode interface, take/donate electrons from/to electrode and at the same time change into their corresponding reduction/oxidation state. To maintain the charge neutrality in the redox electrolyte compartments, cations/anions in the saline stream migrate through the IEMs and into the redox catholyte/anolyte electrolyte compartments, thereby realizing desalination. The CDI architecture with redox electrolyte has various configurations. Bielectrolyte as shown in Figure 3f can be applied in one cell, such as the VCl_3_/NaI system,^[^ ^87^ ^]^ using the V^3+^/V^2+^ and I^−^/I_3_^−^ redox couples to remove anions and cations, respectively. Some cells such as ZnCl_2_/K_4_FeCN_6_ system involve a Zn^2+^/Zn redox couple with liquid–solid transition.^[^ ^88^ ^]^ This system achieved a high salt removal of 85% for simulated seawater (35 g L^−1^ NaCl) and 86% for hypersaline brine (100 g L^−1^ NaCl). It also can use a redox catholyte in one side of cell coupled with a carbon or Faradaic material as the other electrode, such as a carbon/NaI system^[^ ^85^ ^]^ and NASICON/NaI system.^[^ ^89^ ^]^ It is also possible to achieve continuous operation by using the same [Fe(CN)_6_]^4−^/[Fe(CN)_6_]^3−^ redox couple as both the catholyte and anolyte.^[^ ^84^ ^]^ In the electrochemical cell with redox electrolyte, IEMs must be used for the separation of electrolyte and feedwater stream. Moreover, the redox‐active ions dissolved in electrolyte must be prevented from diffusing into the effluent stream, which not only causes the loss of redox‐active substances and the degradation of desalination performance, but also leads to the pollution of the effluent.^[^ ^85^, ^90^ ^]^ Therefore, it is critical to develop advanced membranes, such as ceramic IEMs,^[^ ^89^ ^]^ to minimize the leaching of redox‐active ions.

#### 2.1.2 Comparison and Analysis

These different types of CDI architectures have their merits and shortcomings. Even the conventional CDI architecture that employs only capacitive carbon electrode remains attractive for practical applications, since it has the simplest configuration and uses low‐cost porous carbon materials. Conventional CDI is well suited for the rapid desalination of low salinity feedwaters. MCDI and flow‐electrode CDI enables good performance in higher salinity streams and even continuous operation. On the other hand, CDI architectures based on Faradaic electrodes offer great advantages for treating high salinity streams or even seawater with higher salt removal capacity and lower energy consumption. However, CDI architectures based on Faradaic electrodes also have their respective drawbacks. For the popular Faradaic CDI architecture, the development of Cl^−^ capture Faradaic electrodes is currently lacking in progress. With respect to the HCDI, the salt removal capacity of the carbon electrode inevitably limits the overall device performance. Comparatively, the NID architecture fundamentally avoids the issue of anion storage but requires a relatively complex configuration and operation. CDI architecture with redox electrolyte enables continuous operation but the leaching of redox‐active ions into the effluent stream remains an issue. Therefore, optimized designs and more novel architectures are urgently required to make CDI technology more practical and competitive.

#### 2.1.3 Advanced Integration

The integration of CDI systems with other electronic devices will bring the Faradaic electrode‐based CDI architectures closer to achieving practical targeted goals. Some strategies and possible directions are summarized below.

**Figure 4:**
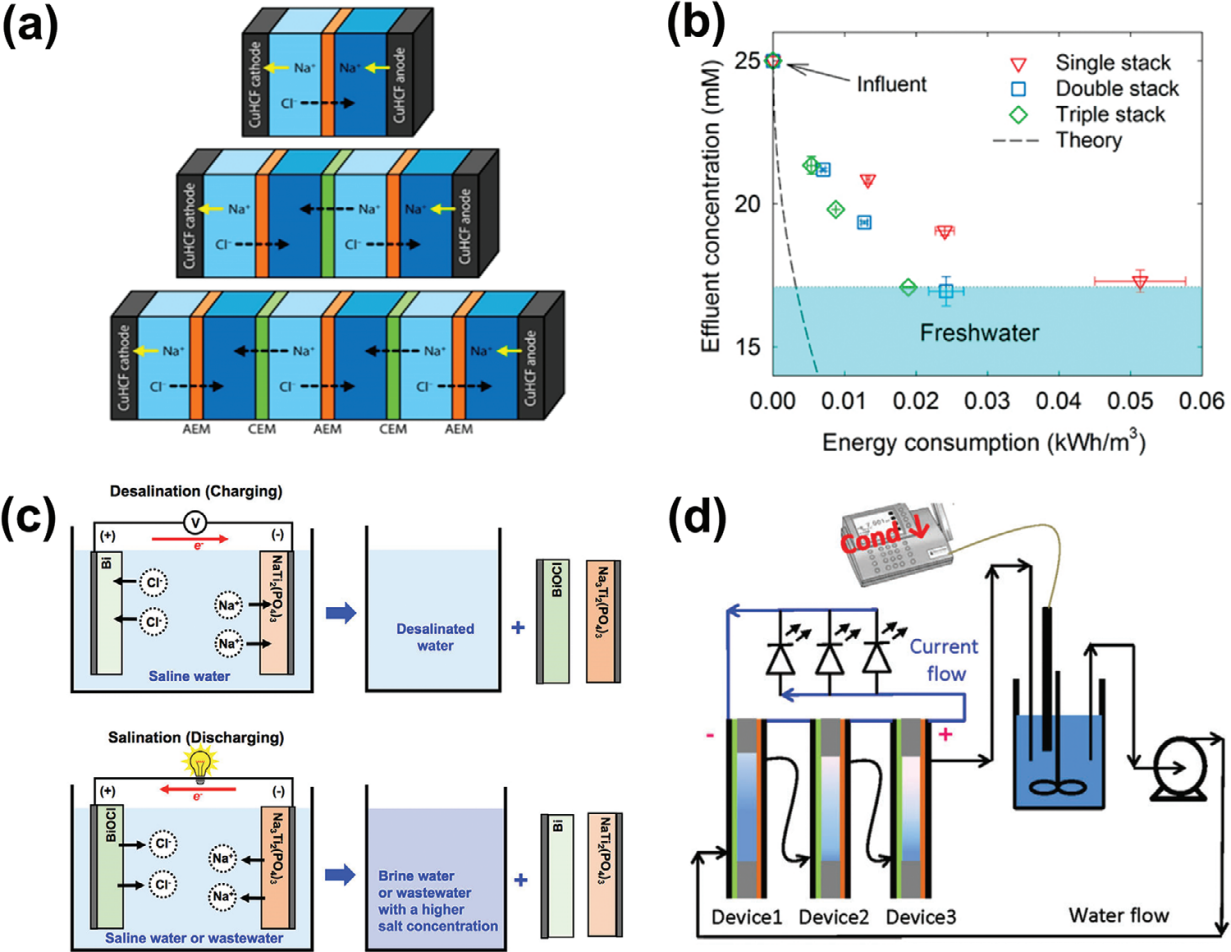
a) Schematic describing the NID cells with one single, double, and triple‐stacked cell, b) effluent concentration versus energy consumption for three different stacked cells (influent: 25 × 10^−3^ m NaCl solution). Reproduced with permission.^[^ ^52^ ^]^ Copyright 2017, American Chemical Society. c) Operational scheme of the NaTi_2_(PO_4_)_3_//Bi CDI cell during desalination process and salination process. Reproduced with permission.^[^ ^43^ ^]^ Copyright 2017, American Chemical Society. d) Schematic representation of the setting‐up with three Na_0.44_MnO_2_//BiOCl devices in series and three LED bulbs connected in parallel. Reproduced with permission.^[^ ^47^ ^]^ Copyright 2017, The Royal Society of Chemistry.

1) Scale‐up. Based on the study of these basic existing CDI architectures, further transformations and improvements toward larger scale using stacked structures can be performed. For example, as shown in **Figure** **4**a, Kim et al.^[^ ^52^ ^]^ transformed the NID from a single cell into a double or triple‐stacked cell by adding the appropriate number of alternating AEMs and CEMs between two CuHCF electrodes. The advanced arrangement of IEMs, feedwater channels, and electrodes improves the degree of desalination significantly. A salt removal capacity of nearly 100 mg g^−1^ can be achieved for the triple‐stacked cell with 50 × 10^−3^ m NaCl solution, and the corresponding energy consumption (≈0.02 kWh m^−3^) fell by 1/3 compared to the single cell (Figure 4b), which is almost 10 times lower than that of the energy reported for the conventional CDI with IEMs (≈0.2 kWh m^−3^).
2) Energy recovery. Desalination and regeneration (salination) are carried out through continuous charge–discharge processes, clearly accompanied with energy input and output. The released energy can potentially be used for work to help recover the consumed energy. With this in mind, Nam et al.^[^ ^43^ ^]^ proposed an ideal operational scheme of the NaTi_2_(PO_4_)_3_//Bi CDI cell connected in series with a bulb as shown in Figure 4c. In this system, the discharge during the salination process generates an energy output that can power the lightbulb. Another example is a proof‐of‐concept closed‐loop system proposed by Chen et al.,^[^ ^47^ ^]^ which was demonstrated for the first time. Specifically, this system consists of three Na_0.44_MnO_2_//BiOCl devices in series and three LED bulbs connected in parallel, as shown in Figure 4d. In this system, salination firstly occurs during charge process, and then the salt removal is achieved during discharge process and the energy out is generated to light the three LED bulbs in the circuit. In summary, this system combines desalination and energy generation at the same time. Energy saving should be considered as a key part of the CDI system, and more effort should be put forth to explore electronic and electrochemical strategies to perform regeneration effectively.
3) Self‐sustainable desalination system. Integrating some energy‐harvesting devices (e.g., solar‐driven devices) with CDI cells to construct self‐sustainable or energy‐efficient desalination systems has also attracted research interest.^[^ ^91^, ^92^, ^93^ ^]^ For example, Ramalingam et al.^[^ ^91^ ^]^ reported a novel desalination system involving internal integration of a dye‐sensitized solar cell and redox‐flow desalination cells with a bifunctional platinized‐graphite‐paper intermediate electrode. By applying visible light illumination, saline solution can be continuously desalinated to freshwater level. Silambarasan et al.^[^ ^93^ ^]^ proposed a new concept of chemical assisted electrochromic desalination system, and demonstrated practical implementation by using metal hexacyanoferrate cathode and an anode consisting of Ag or redox‐active polymer film in brackish water without applying any external electrical energy. In brief, such new device concepts and designs can promote the development of desalination technologies in remote regions with limited electricity.

### 2.2 Key Evaluation Metrics for CDI Performance

Establishing the evaluation metrics for CDI performance is critical to compare various CDI systems and accelerate their development. In terms of the source of effluent water, the salt removal experiment of CDI system can be conducted with two common approaches.^[^ ^40^, ^46^, ^47^ ^]^ One approach is close to practical CDI applications, called the single‐pass (SP) experiment,^[^ ^40^, ^46^ ^]^ where the ionic conductivity of the outlet stream is detected directly at the exit of the CDI cell and the outlet stream is discarded afterwards. Another simpler and emerging approach is the batch‐mode (BM) experiment,^[^ ^47^, ^94^ ^]^ in which the effluent is recycled to the feed water tank to form a water flow circuit, and the ionic conductivity is measured in this tank. As a consequence, the measured desalination performance is quite different between these two experimental approaches. Moreover, the operational mode to drive the CDI system^[^ ^40^, ^46^, ^47^ ^]^ (i.e., constant voltage (CV) mode vs constant current (CC) mode), and the regeneration method in CV mode^[^ ^95^ ^]^ (i.e., zero‐voltage vs reversed‐voltage) will also affect the CDI performance. In addition to the experimental setups described above, other factors could also strongly affect the CDI performance include operational parameters such as initial salt concentration and flow rate, and intrinsic parameters such as such as electrode material types and their properties. Correspondingly, to further enhance the CDI performance by adjusting these factors, related evaluation metrics are indispensable. Key metrics and the recently‐emerged CDI Ragone plot to evaluate CDI performance are described in the following sections.

#### 2.2.1 Salt Removal Capacity

Salt removal capacity (or desalination capacity) is the most intuitive metric to evaluate the CDI performance, which represents the amount of salt removed per unit of electrode material. The salt removal capacity for a single‐step salt removal is calculated by dividing the mass of the removed salt by the total mass of the CDI cell electrodes, as defined in Equations (1) for SP experiment and (2) for BM experiment. From the definition, the salt removal capacity reflects the desalting capability of the electrode itself under certain testing conditions or operational parameters. Therefore, the salt removal capacity metric guides the optimal design of electrode materials

(1) $$\begin{array}{c} \mathrm{SP}:\mathrm{salt}\;\mathrm{removal}\;\mathrm{capacity}=\frac{\Phi \int \Delta \mathrm{Cdt}}{m} \end{array}$$

(2) $$\begin{array}{c} \mathrm{BM}:\mathrm{salt}\;\mathrm{removal}\;\mathrm{capacity}=\left(C_{0}-C_{t}\right)V/m \end{array}$$

where the *Φ* is the flow rate (L min^−1^), Δ*C* is the real‐time salt concentration change of outlet water (mg L^−1^), *m* is the total mass of electrodes (g), *C*_0_ and *C*_t_ are the initial and equilibrated NaCl concentrations of a salt removal step (mg L^−1^), respectively. *V* represents the total volume of the NaCl solution (L). The unit of salt removal capacity is mg g^−1^.

The measured salt removal capacity is mainly influenced by two operational parameters: the influent salt concentration and the current density in CC mode or the voltage in CV mode. For the former, a common trend for CDI cells employing Faradaic electrode materials is that an increase in influent salt concentration increases the salt removal capacity, and the trend remains even when the salinity is as high as seawater in some studies.^[^ ^59^, ^96^ ^]^ This could be due to the following reasons. First, a higher influent salt concentration reduces the ionic resistance of the electrolyte, thus enhancing the electrochemical activity of the electrodes.^[^ ^41^, ^95^ ^]^ Second, a higher influent salt concentration (i.e., higher ionic strength) leads to a reduced effective size of the hydrated ion (i.e., lower number of water molecules in the hydration shell), facilitating a more facile and effective ion insertion.^[^ ^97^, ^98^, ^99^ ^]^ Finally, a higher influent salt concentration enhances the concentration gradient between the flow channel and the electrode, as well as within the electrode, thus improving the diffusion and convection effect.^[^ ^100^ ^]^ In contrast, conventional CDI cells with carbon electrode materials demonstrate the opposite behavior, that is, the co‐ion expulsion effect will be intensified with increasing salt concentrations, leading to ineffective desalination performance at concentrations above 3 g L^−1^.^[^ ^100^ ^]^

As mentioned, the other important influencing factor for salt removal capacity is the magnitude of current density or voltage. In the CC mode, the salt removal capacity is inversely proportional to current density.^[^ ^47^, ^101^, ^102^ ^]^ This trend is ascribed to the increased voltage drop (IR) in the CDI cell, which reduces the charge/discharge time, thus leading to incomplete reactions between the ions and electrode materials.^[^ ^47^, ^94^ ^]^ For the CV mode, applying a higher voltage will enhance the salt removal capacity due to the increased charge build‐up.^[^ ^46^, ^58^, ^103^ ^]^ However, excessively high voltages will also generate some parasitic redox reactions, such as the electrolysis of water (1.23 V)^[^ ^104^ ^]^ and the oxidation of Cl^−^ to Cl_2_ gas (1.36 V).^[^ ^105^ ^]^ Thus most studies adopt a potential with an upper limit of 1.2–1.4 V.^[^ ^40^, ^46^, ^58^, ^106^, ^107^ ^]^ The reason that slightly higher potentials (e.g., 1.4 V) can be applied in some experiments, is that these parasitic redox reactions do not occur due to the resistance in real CDI circuits.^[^ ^106^, ^107^ ^]^

#### 2.2.2 Salt Removal Rate

The salt removal rate (or desalination rate) is another key evaluation metric for a desalination cell,^[^ ^108^, ^109^, ^110^ ^]^ which reflects the salt removal capacity per unit time and can be calculated by dividing salt removal capacity by the salt removal time, as defined in Equation (3)

(3) $$\begin{array}{c} \mathrm{salt}\;\mathrm{removal}\;\mathrm{rate}\;=\frac{\mathrm{salt}\;\mathrm{removal}\;\mathrm{capacity}}{t} \end{array}$$

where the *t* is the operational time of a salt removal step (s). The unit of salt removal rate is mg g^−1^ s^−1^.

Similar factors mentioned in salt removal capacity will also affect the value of salt removal rate. Increasing the influent salt concentration,^[^ ^41^, ^111^ ^]^ current density in CC mode^[^ ^8^, ^94^, ^102^ ^]^ or the applied voltage in CV mode^[^ ^107^, ^111^ ^]^ can contribute positively to the salt removal rate of the desalination cell up to a certain extent. It should be noted that although an increase in current density increases salt removal rate, the improvement of salt removal rate with Faradaic electrodes normally comes at the expense of a sharp loss of salt removal capacity. Previous studies with Faradaic electrodes show that the salt removal capacity drops by 50% or more as the current density increases from 100 to 200 mA g^−1^.^[^ ^47^, ^102^, ^112^ ^]^ This phenomenon limits desalination tests using Faradaic electrodes to a low applied current density of 100 mA g^−1^,^[^ ^47^, ^102^ ^]^ which is an order of magnitude lower than that for capacitive carbon electrodes based on facile electrosorption mechanism.^[^ ^51^, ^110^, ^113^, ^114^ ^]^ Regarding this issue, improvements in the salt removal rate of Faradaic electrodes typically involve designed materials with advanced structures, such as porous 3D structures or nanostructures to shorten the diffusion length of salt ions, or incorporating highly conductive carbon into Faradaic electrodes to provide facile electron conduction paths.^[^ ^43^, ^58^, ^94^, ^115^, ^116^ ^]^ In addition, it is evident that charge time is also an important factor that affects the value of salt removal rate. In general, salt removal rate rises rapidly at the beginning of desalination and peaks at a maximum value, and then decreases gradually until the system reach equilibrium when salt ions can no longer be removed.^[^ ^46^, ^78^, ^111^ ^]^ Hence shortening the charge time intentionally will lead to higher salt removal rate. Moreover, operating the system with thinner electrodes can facilitate convection effect and thus result in higher salt removal rate as well.^[^ ^114^, ^116^ ^]^

#### 2.2.3 Salt Removal Efficiency

Salt removal efficiency (*η*), or desalination efficiency, is the percentage of salt removed from the saline solution in a closed‐loop system (generally refers to BM experiment),^[^ ^9^, ^117^ ^]^ as defined in Equation (4)

(4) $$\begin{array}{c} \eta =\frac{C_{0}-C_{t}}{C_{0}}\times 100\% \end{array}$$

This metric is directly affected by many operational parameters, such as the flow rate and solution volume. Moreover, some other parameters that significantly affect salt removal capacity (such as initial influent concentration, current density, or applied voltage) also have indirectly influence on salt removal efficiency, resulting in a more complex outcome.^[^ ^118^, ^119^ ^]^ Therefore, salt removal efficiency is commonly used for comparing the salt removal abilities of different electrodes under the exact same CDI experimental conditions. Since Faradaic electrodes can exhibit higher SAC than capacitive carbon electrode, consequently it can achieve higher salt removal efficiency in the same test cell.^[^ ^117^, ^118^, ^120^ ^]^

#### 2.2.4 Flow Efficiency

Flow efficiency is an appropriate metric that reflects the ion flow in a destination device.^[^ ^68^, ^121^ ^]^ This metric depends on the residence time for an ion to pass through the device and the half‐cycle time for a given desalination, as calculated by the Equation (5)

(5) $$\begin{array}{c} \mathrm{flow}\;\mathrm{efficiency}=\frac{f-2\mathrm{nLp}}{2\mathrm{fT}} \end{array}$$

where *f*, *n*, *L*, *p*, and *T* represent the superficial velocity of the pore solution (cm s^−1^), the number of interfaces between different electrodes in a device stack, the thickness of electrode (cm), the porosity of the electrode, and the half‐cycle time of a given desalination (s), respectively. Flow efficiency is important for regulating the amount of processed water relative to the amount of remediated water. Flow efficiency is also helpful to set the feed rate of water during the cyclic operation of a desalination device in order to flush the immobilized ions from the electrodes.

#### 2.2.5 Charge Efficiency and Energy Consumption

Charge efficiency (Λ) is defined as the ratio of the amount of removed salt to the electric charge passing through the electrode during a given ion removal step, as calculated by the Equations (6) and (7). Another key metric closely related to charge efficiency is the energy consumption. In the CDI community, few studies express the energy consumption as the amount of salt removed for per unit of energy consumption (usually in terms of g Wh^−1^),^[^ ^41^ ^]^ while most other studies represent it as the inverse (usually in terms of Wh g^−1^, Wh L^−1^, J mol^−1^, or kT).^[^ ^39^, ^55^, ^78^, ^102^, ^122^ ^]^ Here, we recommend and base all discussion below on the latter definition, with the corresponding calculation shown in the Equations (8) and (9)

(6) $$\begin{array}{c} SP:\;\Lambda =\frac{\Phi \int \Delta C\mathrm{dt}}{M_{S}\int I\mathrm{dt}/F}\times 100\% \end{array}$$

(7) $$\begin{array}{c} BM:\;\Lambda =\frac{\left(C_{0}-C_{t}\right)V}{M_{S}\int I\mathrm{dt}/F}\times 100\% \end{array}$$

(8) $$\begin{array}{c} SP:\;E_{S}=\frac{M_{S}\int UI\mathrm{dt}}{\Phi \int \Delta C\mathrm{dt}} \end{array}$$

(9) $$\begin{array}{c} BM:\;E_{S}=\frac{M_{S}\int UI\mathrm{dt}}{\left(C_{0}-C_{t}\right)V} \end{array}$$

where the *M*_s_ is the molar mass of NaCl (58.5 g mol^−1^), *I* is the current (A), *U* is the voltage (V), and *F* is Faraday's constant (96 485 C mol^−1^). The unit of *E*_s_ is J mol^−1^, and the J mol^−1^ can also be converted into kT (an energy unit as the product of the absolute temperature and Boltzmann constant) by dividing the calculated energy (J mol^−1^) by the gas constant and absolute temperature RT (2.48 kJ mol^−1^ at 25 °C).^[^ ^55^, ^122^ ^]^

These two metrics are meaningful for comparing different CDI systems and even other desalination technologies.^[^ ^6^ ^]^ It can be seen from the definitions and desalination studies that there is an inverse relationship between charge efficiency and energy consumption. Many operational parameters have significant influence on energy consumption and charge efficiency. Normally, for CDI cells with Faradaic electrode materials, a higher influent salt concentration leads to a higher charge efficiency and lower energy consumption.^[^ ^41^, ^96^ ^]^ The reduced ionic resistance of the solution with higher salinity facilitates ion transport, and thus the electrode requires less electric charge and less energy to remove salt ions. On the other hand, for carbon electrode materials a higher salinity exacerbates co‐ion expulsion, thus requiring an increasing electric charge and drastically increased energy consumption for ion removal.^[^ ^55^ ^]^ For instance, CDI cells with Faradaic electrodes, such as V_2_O_5_‐multiwalled CNT (17.9 kT for 600 × 10^−3^ m NaCl solution)^[^ ^123^ ^]^ and Mo_1.33_C‐CNT (17 kT for 600 × 10^−3^ m NaCl solution),^[^ ^96^ ^]^ exhibit relatively low energy requirements even at seawater concentration of 600 × 10^−3^ m NaCl. These energy consumption values are comparable to CDI systems using carbon electrode with IEMs (20–25 kT for 10 × 10^−3^–200 × 10^−3^ m NaCl solution).^[^ ^25^, ^70^ ^]^ Another case in point is demonstrated by Srimuk et al.,^[^ ^55^ ^]^ who employed MoS_2_/CNT electrodes and reported an energy consumption of 24 kT for 500 × 10^−3^ m NaCl feedwater, which is in distinct contrast with the tremendous energy requirement of 20 000 kT when treating the same solution with activated carbon electrodes. Therefore, CDI with Faradaic electrode materials is not only superior compared to carbon electrodes in salt removal capacity, but is also much less energy intensive.

The applied voltage also has an important impact on energy consumption. For constant voltage mode, an increase in voltage will increase salt removal capacity. However, higher voltage signifies more electric charge on electrode and larger resistance in the system, and subsequently increases the energy consumption. When the increased salt removal capacity does not compensate for the increased energy consumption, it will lead to energy waste. Therefore, we must gain a balanced view of desalination capacity, charge efficiency, and energy consumption to select a suitable operational voltage, in order to achieve the goal of larger salt removal capacity and lower energy consumption. It is also worth noting that current studies about the calculation of the energy consumption only take into account the energy supplied during desalination account, while not considering the pumping energy required to drive liquid flow and the energy that can potentially be recovered during regeneration. Future studies on energy consumption should add the pump energy and deduct the energy recovered from recovery technologies (see Section 2.1) to reflect actual conditions.

#### 2.2.6 Cycling Performance

The cycling performance of CDI cells plays a critical role in practical desalination processes. For a given set of experimental conditions, the cycling performance depends on the CDI cell itself, especially with the regeneration behavior of electrode. To be specific, when the desalination process reaches an equilibrium, if a reverse voltage (or open circuit) in CV mode or a reverse current in CC mode was applied, the electrodes would release the captured ions back into the solution. The completion of this regeneration process represents a CDI cycle process.

Initial CDI studies with Faradaic electrodes were usually limited within 20–60 cycles.^[^ ^47^, ^53^, ^55^, ^58^, ^77^, ^94^, ^96^ ^]^ Large volume changes and low electrical conductivity can significantly affect the electrochemical stability and reversibility of Faradaic electrode materials. Therefore, some researches have been devoted to enhancing the cycling performance of CDI cell with Faradaic electrode materials.^[^ ^95^, ^115^, ^116^, ^124^ ^]^ Building 3D frameworks or nanostructures with high dispersion, and introducing carbon materials with intrinsically excellent electrical conductivity into Faradaic electrode materials, can suppress volume change and enhance the electrical conductivity. Consequently, the lifetime of desalination cell can be effectively extended. For instance, the employing of NaTi_2_(PO_4_)_3_/rGO electrode in CDI cell can extend the lifetime to 100 cycles.^[^ ^95^, ^116^ ^]^ Moreover, other electrode materials of Na_3_V_2_(PO_4_)_3_@C^[^ ^115^ ^]^ or FeFe(CN)_6_ nanocube@nanoporous graphene^[^ ^124^ ^]^ can further prolong the lifetime of CDI cell to 500 cycles and 600 cycles, respectively.

#### 2.2.7 CDI Ragone Plot

The CDI Ragone plot (or Kim‐Yoon plot) has recently been proposed and is widely used to represent and evaluate performance,^[^ ^77^, ^107^, ^111^, ^125^ ^]^ in which salt removal rate is plotted against salt removal capacity, similar to the Ragone plot of power density versus energy density in the energy storage field. This plot visually represents both the capacity and rate performance in one diagram, from which researchers can directly judge whether these two key metrics meet the expected goals or not. Furthermore, the CDI Ragone plot can be used to visually reflect the impact of different operational parameters on salt removal capacity and salt removal rate, allowing for optimization of the appropriate conditions.^[^ ^77^, ^107^, ^111^ ^]^

The relationship between cell system design and desalination performance is complicated. Although we have already introduced some typical influencing factors (for example, electrode material type, the current density in CC mode or the voltage in CV mode, and influent salt concentration) on the above‐mentioned performance metrics, in fact, the influencing factors for these performance metrics are far more than these. Specifically, the performance metrics are also influenced by electrode engineering (such as packing density, mass loading, thickness, and the type and amount of conductive additive), cell engineering (such as cell architectures, with/without IEMs, the balance between cathode and anode), and operational parameters (such as SP/BM experiment approach, CV/CD mode, zero‐voltage/reversed‐voltage regeneration method, and flow rate). For example, increasing the flow rate within a certain range will increase the salt removal capacity, whereas too high flow rate results in the flushing out of electrode materials and, thus, low salt removal capacity.^[^ ^101^, ^116^, ^124^ ^]^ Another example, increasing the thickness and the mass loading of the electrode will lead to lower salt removal rate.^[^ ^114^, ^116^ ^]^ Therefore, it is difficult to compare the pros and cons of different CDI cell as well as different electrode materials. To make a valuable comparison of desalination performances in CDI community, all the critical CDI system design parameters should be explicitly mentioned. More importantly, it is urgent to draw a set of standard system parameters to enable a fair comparison between different cells.

### 2.3 Ion Capture Mechanisms

**Figure 5:**
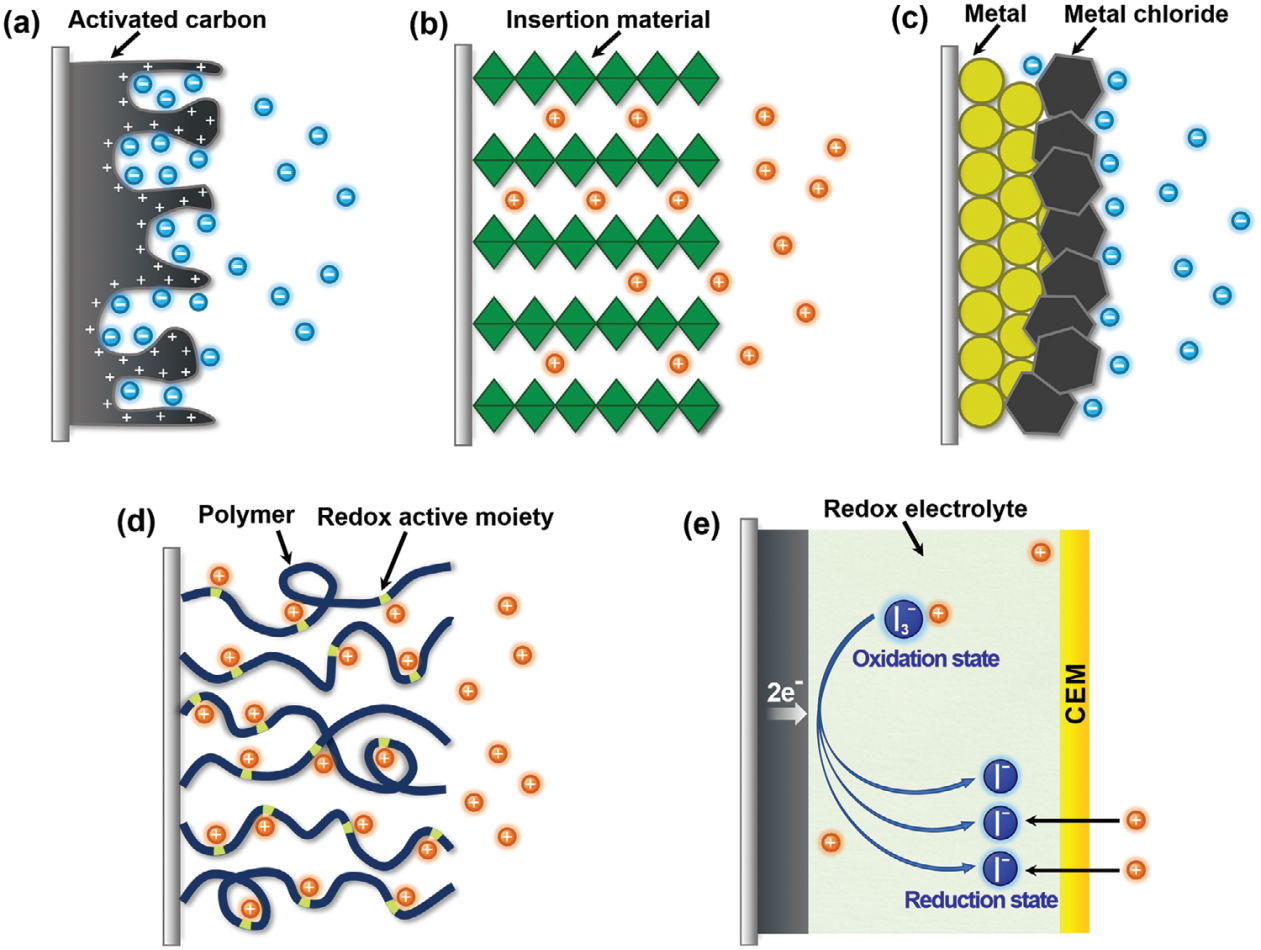
Different types of ion capture mechanisms: a) electrosorption, b) insertion reaction, c) conversion reaction, d) ion‐redox active moiety interaction, and e) charge compensation with redox‐active electrolyte.

Among the above‐mentioned factors affecting CDI performance, the electrode material clearly plays a critical role. To further understand the roles of electrode materials in desalination, ion capture mechanisms are emphasized in this section. As shown in **Figure** **5**, the ion capture mechanisms of CDI cell with various electrodes can be classified to two main types: including electrosorption and Faradaic reaction. The former occurs in conventional CDI cells with carbon electrodes as displayed in Figure 5a, in which the adsorption of oppositely charged salt ions from the solution onto the surface of carbon materials is attributed to the potential difference.^[^ ^8^, ^12^ ^]^ The latter mechanism occurs in Faradaic materials as shown in Figure 5b–e, which includes 1) insertion reaction, 2) conversion reaction, 3) ion‐redox active moiety interaction, and 4) charge compensation with redox‐active electrolyte. In comparison, Faradaic electrode materials capture ions by Faradaic reactions that occur throughout the bulk material and are not limited to the surface.

The insertion reaction is based on the insertion of ions into interstitial sites of the electrode material through a Faradaic charge‐transfer process, as displayed in Figure 5b. In the CDI community, most insertion materials are capable of cation insertion, and few are capable of anion insertion (e.g., calcined layered double hydroxides). Some are capable of both cation and anion insertion (e.g., MXenes, and transition metal dichalcogenides). According to different spatial dimensions of the channels for ion transport, insertion materials can be further subdivided into three categories: 1D, 2D, and 3D insertion materials. 1D insertion materials have tunnel structures for ion diffusion and storage, such as Na_0.44_MnO_2_ with large S‐shaped tunnels,^[^ ^47^, ^126^ ^]^ or tunnel structured manganese/titanium oxides with tailorable tunnel shapes and sizes.^[^ ^109^, ^127^ ^]^ These tunnels provide space for ion diffusion and storage. 2D insertion materials involve layered materials, such as layered manganese/titanium/vanadium oxide compounds,^[^ ^128^, ^129^, ^130^ ^]^ calcined layered double hydroxides,^[^ ^57^ ^]^ MXenes,^[^ ^58^ ^]^ or transition metal dichalcogenides.^[^ ^131^ ^]^ In these materials, ions can be inserted within the interlayer space. 3D insertion materials have open 3D framework structures, such as NASICON‐type phosphates^[^ ^41^, ^132^ ^]^ or metal hexacyanometalates.^[^ ^59^, ^133^ ^]^ These materials provide 3D transport channels for ion diffusion, and accommodate inserted ions into specific crystal structural sites of 3D framework structures. The structural features and corresponding insertion mechanism of each insertion materials are detailed in Section 3. Ion insertion/extraction does not form chemical changes, but tends to be accompanied by structural change, which often occurs in 2D insertion materials.^[^ ^55^, ^128^, ^134^ ^]^ The insertion of ions typically causes an increase in interlayer distance of materials,^[^ ^128^, ^134^, ^135^ ^]^ and may even trigger phase transformations. One classic example is how the insertion of Na^+^ into MoS_2_ with 2H phase causes an expansion of interlayer distance, and further insertion is accompanied by a phase change from 2H phase to 1T phase.^[^ ^55^ ^]^ These structural changes will aggravate gradually over continuous ion capture/release cycles, leading to capacity fading. Thus, some strategies, like pretreatment through pre‐insertion^[^ ^134^ ^]^ and pre‐exfoliation,^[^ ^136^ ^]^ or designing porous 3D structures^[^ ^58^ ^]^ are required to better accommodate Na^+^ and mitigate volume change issue.

The conversion reaction mechanism involves a chemical transformation to form a new compound, as displayed in Figure 5c. To date, conversion materials for water desalination mainly involve Cl^−^ capture materials, including Ag/AgCl^[^ ^39^, ^42^ ^]^ and Bi/BiOCl.^[^ ^43^, ^47^ ^]^ Take Ag/AgCl for example. Upon oxidation, the Cl^−^ in solution reacts with Ag, resulting in the breaking of the Ag‐Ag bond and the formation of AgCl. Conversion reactions allow electrode materials to generate high theoretical specific capacities. However, the conversion reaction is accompanied by tremendous volume expansion and contraction of electrode (>150%), and repetitive chemical transformation leads to particle coarsening, resulting in poor electrode stability. In addition, the conversion reaction is kinetically sluggish, which poses a challenge to realize the theoretical capacity. Therefore, further advanced modifications, such as nanostructuring and methods to form carbon composite, should be explored to address these issues.

The third mechanism is the ion‐redox active moiety interaction as displayed in Figure 5d, which typically exists in polymers with redox active moieties. According to the position of the redox active moiety in the polymer chain, redox‐active polymers can be broadly classified into two categories: 1) redox‐active moiety‐suspended polymers, which have redox‐active moieties on their nonconductive polymer backbones; and 2) redox‐active moiety‐embedded polymers, which are mainly conjugated conductive polymers with redox‐active backbones consisting of active monomers. Redox‐active polymers show selective affinity toward either cations or anions depending on their specific redox active moieties. For example, as typical redox‐active moiety‐suspended polymers, polymers with carbonyl groups (i.e., polyimides and polyquinones) can generally undergo redox reactions by interacting with cations such as Na^+^.^[^ ^56^, ^137^ ^]^ As typical redox‐active moiety‐embedded polymers, p‐type conducting polymers such as polypyrrole and polyaniline can undergo p‐doping/dedoping reactions to capture/release Cl^−^.^[^ ^138^, ^139^ ^]^ Moreover, polymers can also be endowed with electrochemical activity by integrating redox‐active moieties on polymer backbones, such as by grafting electron donor/withdrawing groups (such as —SO_3_^−^ group for Na^+^ capture),^[^ ^140^ ^]^ or doping with reactive inorganic ions (such as [Fe(CN)_6_]^4−^ for Cl^−^ capture).^[^ ^44^ ^]^ Details for the ion‐redox active moiety interaction of polymers will be specified in Section 3.3.3. Comparatively, this mechanism is more kinetically facile than the conversion mechanism, because no new compound forms and structural evolution does not occur in the ion‐redox active moiety interaction. However, polymers with this mechanism still have some limitations to be overcome, such as the poor intrinsic electronic conductivity within nonconductive polymer backbones, and deficient redox active moieties along with comparatively low ion capture capacity. With the deepening of research on polymers, some reports of high desalination performance have also appeared,^[^ ^118^, ^141^, ^142^ ^]^ creating new opportunities for the development of polymer‐based electrochemical cells with high desalination performance.

In contrast to the above‐mentioned ion capture mechanisms of Faradaic materials where the removed ions directly participate in the Faradaic reactions, ions can be removed as a consequence of charge compensation for redox‐active ions dissolved in electrolyte.^[^ ^84^, ^86^, ^88^ ^]^ Take the I^−^/I_3_^−^ redox couple in Figure 5e as an example. Once charge buildup in the electrode is occurs, the I_3_^−^ moves close to the electrolyte‐electrode interface, takes two electrons from electrode and becomes I^−^ with more negative charges (I_3_^−^ + 2e^−^ ↔ 3I^−^). To balance the charge buildup in the redox electrolyte solution, two Na^+^ ions move through the CEM into the electrolyte. Upon oxidation of I^−^ to I_3_^−^, Na^+^ ions are ejected through the CEM. Many possible catholyte redox couples (e.g., I^−^/I_3_^−^, Br^−^/Br_3_^−^) and anolyte redox couples (e.g., V^3+^/V^2+^, Zn^2+^/Zn) have can be used to capture ions.^[^ ^68^ ^]^ These redox couples may enable high charge‐storage capacities (e.g., 168±50 mAh·g^−1^ for 1 m NaI^[^ ^85^ ^]^) when multielectron redox reactions are involved. Redox couples are not always in ionic form, and may also involve liquid–solid transitions, such as the Zn^2+^/Zn couple. In the system consisting of a Zn^2+^/Zn anolyte and a bromide‐containing catholyte, cations and anions can migrate from feedwater stream to the catholyte and anolyte compartments respectively through charge compensation driven by the redox reactions (Br_3_^−^ → 3Br^−^ + 2e^−^ and Zn + 2e^−^ → Zn^2+^)^[^ ^86^ ^]^ The [Fe(CN)_6_]^4−^/[Fe(CN)_6_]^3−^ redox couple can even be used as both the catholyte and anolyte.^[^ ^84^ ^]^ The sustainable redox reaction allows continuous desalination operation (i.e., without discharging steps) and is capable of desalinating high salinity streams up to seawater level. However, when employing redox electrolyte, it is important to prevent redox‐active ions from entering the middle feedwater compartment to avoid contamination and performance degradation.^[^ ^85^, ^90^ ^]^ Thus, it is necessary to assemble the cell with advanced IEMs.

### 2.4 Basic Requirements for High Performance Faradaic Electrode Materials

An ideal CDI cell with Faradaic electrodes should demonstrate favorable performance metrics, such as a high salt removal capacity, fast salt removal rate, and long cycle life with low energy consumption as summarized earlier. To achieve this goal, the Faradaic electrode materials suitable for CDI application should meet the requirements as follows:

Permselectivity: The Faradaic electrode materials should have permselectivity for either the cations or anions, which is a critical point of difference from capacitive carbon electrodes that are significantly affected by co‐ion expulsion.

Stability: The electrodes should be chemically stable from dissolution to avoid secondary pollution on the effluent water, and be structural stability with low volume changes to ensure a good cycling performance. As for electrochemical stability, the redox potentials of Faradaic electrode materials should be located between the oxygen and hydrogen evolution reaction to avoid water decomposition during the electrochemical charge–discharge processes.

Conductivity: Electronic conductivity is important for electrode materials. A low electron conductivity is a common shortcoming of Faradaic electrode materials, as it increases the polarization of electrochemical reactions and decreases the rate of electron transfer, leading to sluggish charge‐transfer kinetics. Adding a conductive agent during the electrode preparation process, and combining carbon and Faradaic materials to obtain hybrid electrode materials are effective strategies to improve charge percolation throughout the electrode,^[^ ^94^, ^115^, ^116^ ^]^ thereby resulting in optimized charge capacity, enhanced rate performance and reduced energy dissipation.

Compatibility: There should be a good match between the ion donor and the ion acceptor in Faradaic electrode‐based CDI cells. To be specific, both electrodes in Faradaic CDI cells, such as Na_0.44_MnO_2_//BiOCl cell,^[^ ^47^ ^]^ Na_3_V_2_(PO_4_)_3_//AgCl cell,^[^ ^94^ ^]^ and NaTi_2_(PO_4_)_3_//Bi cell,^[^ ^43^ ^]^ should be either ion donors (Na‐rich and Cl‐rich materials) or ion acceptors (Na‐poor and Cl‐poor materials) to ensure the normal running of CDI system.^[^ ^43^, ^47^, ^59^, ^94^, ^95^ ^]^ While for NID cells, one electrode should be Na^+^ donor and another be Na^+^ acceptor to ensure an optimal CDI process.^[^ ^50^, ^52^ ^]^

Practicability: Both operational/economic feasibility and safety should be optimal for practical CDI applications. For the former, the CDI cells with Faradaic electrode materials should have low cost and good scalability for practical use. For the latter, safety aspects such as the toxicity of electrodes should be taken into consideration. This is because the desalinated water from CDI may be used for cleaning, raising livestock, irrigation, personal hygiene, and even drinking.

## 3 Faradaic Electrode Materials for CDI

Given that the main salt composition in seawater is NaCl, we divide the Faradaic electrode materials into three categories: Na^+^ capture Faradaic electrode materials, Cl^−^ capture Faradaic electrode materials, and Faradaic electrode materials with special architectures for both Na^+^ and Cl^−^ capture. Specifically, the crystal structural features, physicochemical characteristics, ion capture mechanism, desalination performance and corresponding challenges for these materials will be discussed in detail below.

### 3.1 Na^+^ Capture Faradaic Electrode Materials

#### 3.1.1 Manganese Oxide Compounds

Manganese oxide compounds have various crystal structures with Mn in multiple valence states.^[^ ^34^, ^143^, ^144^, ^145^ ^]^ These compounds generally possess an open crystal structure suitable for large ion insertion and extraction without distinct volume expansion. Moreover, due to the cost efficiency and nontoxicity of Mn, facile synthesis methods, and well‐controlled morphology by regulating reaction parameters, manganese oxide compounds have been extensively used as Na^+^ host structures for Na‐ion batteries and other electrochemical energy storage and conversion devices.^[^ ^34^, ^35^, ^143^ ^]^ Moreover, the structural stability and hydrophilic nature of manganese oxide compounds allow for extended and rapid redox reactions in aqueous environments. Such a combination of characteristics therefore makes these materials increasingly popular in CDI electrodes for water desalination, and here we will focus on two main categories: manganese dioxide with different polymorphs and sodium manganese oxides.

##### Manganese Dioxide

**Figure 6:**
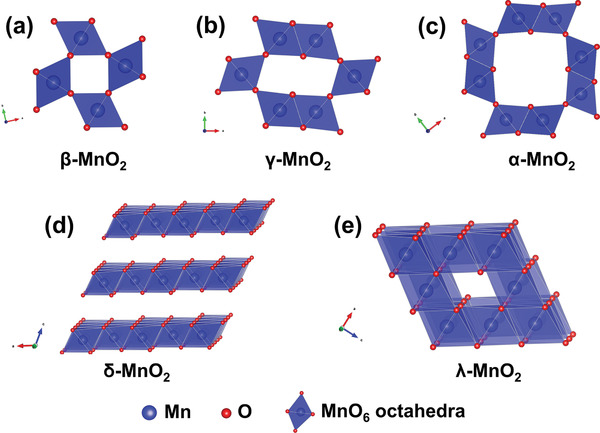
The crystal structure schematic of manganese oxides: a) *β*‐MnO_2_, b) *γ*‐MnO_2_, c) *α*‐MnO_2_, d) *δ*‐MnO_2_, and e) *λ*‐MnO_2_.

Crystallized MnO_2_ materials form a versatile structural family with materials exhibiting 1D‐, 2D‐, or 3D‐type tunnel structures built on MnO_6_ octahedra assemblies.^[^ ^145^, ^146^ ^]^ As shown in **Figure** **6**, 1D tunnel structured MnO_2_ are built up with MnO_6_ octahedra sharing edges and corners, and thus form 1D structural tunnels with different shapes and sizes based on the number of octahedral subunits T(n × m),^[^ ^145^, ^147^ ^]^ such as *β*‐MnO_2_ (1 × 1) (Figure 6a), *γ*‐MnO_2_ (1 × 2) (Figure 6b), and *α*‐MnO_2_ (2 × 2) (Figure 6c). 2D *δ*‐MnO_2_ possesses a layered structure composed of 2D atomic layers of edge‐shared MnO_6_ octahedra, as shown in Figure 6d. 3D *λ*‐MnO_2_ configuration has the cubic Fd$\bar{3}$m unit cell with 3D interconnected tunnels as shown in Figure 6e. These MnO_2_ materials can store alkali metal cations via two types of electrochemical approaches:^[^ ^148^, ^149^, ^150^ ^]^ surface‐dependent Faradaic reaction (pseudocapacitance) and insertion into the bulk material. Both follow the same Faradaic reaction

(10) $$\begin{array}{c} \mathrm{Mn}O_{2}+xA^{+}+xe^{-}↔A_{x}\mathrm{Mn}O_{2} \end{array}$$

in which A^+^ is an alkali metal cation. To date, 1D tunnel structured MnO_2_, 2D *δ*‐MnO_2_, 3D *δ*‐MnO_2_, and amorphous MnO_2_ have been explored as Faradaic electrodes in CDI, and their crystallographic microstructure heterogeneity coupled with the various valence states of Mn in MnO_2_ significantly affect the ion insertion/extraction behavior and corresponding desalination performance.^[^ ^76^, ^103^, ^109^, ^128^ ^]^

1) 1D tunnel structured MnO_2_ (TuMOs)

In addition to the pristine structure of TuMOs as shown in Figure 6a–c, TuMOs with relatively larger tunnels, such as *α*‐MnO_2_ (2 × 2) and todorokite (Tod)‐MnO_2_ (3 × 3), often are filled with foreign cations or water molecules.^[^ ^147^ ^]^ These foreign cations (also called stabilizing cations) are mainly metal ions such as Na^+^, K^+^, Mg^2+^, Ni^2+^, etc., which can force the manganese oxidation state from Mn^4+^ down to Mn^3+^ for charge balance and stabilize the whole crystal structure.

TuMOs with various tunnel sizes and stabilizing ions have been widely explored in ion insertion/extraction. In general, the size of the tunnel cavity in TuMOs, specifically the “free space” in the cavity, is an important factor that influences the ion storage performance. On one hand, the original size of the cavity can directly influence the ion diffusion and storage. TuMOs with larger tunnel cavities supposedly deliver higher capacities. Devaraj et al.^[^ ^149^ ^]^ found that the specific capacitance of MnO_2_ measured in 0.1 m Na_2_SO_4_ solution decreases in the order of *α*‐MnO_2_ (2 × 2) > *γ*‐MnO_2_ (1 × 2) > *β*‐MnO_2_ (1 × 1). A large tunnel size of (4.6 Å) *α*‐MnO_2_ facilitates the insertion of Na^+^ into tunnels, while a narrow tunnel size of (2.3 Å) *β*‐MnO_2_ limits ion insertion resulting in a low capacity, and *γ*‐MnO_2_ displays a capacity between the above two. Another example is proposed by Ghodbane et al.,^[^ ^151^ ^]^ who tested the electrochemical performance of a series of TuMOs and also demonstrated that a larger cavity results in an enhanced capacity for TuMOs as shown in the following order: *β*‐MnO_2_ (1 × 1) < *γ*‐MnO_2_ (1 × 2) < *α*‐MnO_2_ (2 × 2) < 2 × 4‐MnO_2_ (2 × 4).

On the other hand, the presence of stabilizing ions in the tunnel cavity will impede ion diffusion and storage, thus influencing the electrochemical performance. There are two main explanations in this regard: i) One is that the stabilizing ions will reduce the “free space” of the cavity. A classic example is the electrochemical performance of Tod‐MnO_2_ (3 × 3) with hydrated nickel as stabilizing ion inside. Ion insertion/extraction was hindered by the hydrated nickel and the strong hydrogen bonds between the oxygen atoms of the nickel hydration shell and the oxygen atoms of MnO_6_ octahedra, thus resulting in a comparatively sluggish capacitance even compared to *γ*‐MnO_2_ with a smaller tunnel space (1 × 2).^[^ ^151^, ^152^ ^]^ (ii) The other aspect is that the presence of stabilizing ions could reduce the manganese oxidation state value and further influence the ion storage performance. For example, Byles et al.^[^ ^109^ ^]^ prepared a series of TuMOs and explored their ion removal performance using HCDI cells in 15 × 10^−3^ m KCl solutions, in which the *α*‐MnO_2_ (2 × 2) with K^+^ stabilizing ions (evaluated chemical composition: K_0.11_MnO_2_) exhibited a higher ion removal capacity (32.2 mg g^−1^) than Tod‐MnO_2_ (3 × 3) with Mg^2+^ stabilizing ions (27.7 mg g^−1^, evaluated chemical composition: Mg_0.20_MnO_2_). The comparatively lower manganese oxidation state (+3.6) of Tod‐MnO_2_ compared to *α*‐MnO_2_ (+3.89) was expected to decrease its ion removal capacity according to the Faradaic reaction Equation (10), despite the large cavity size.

2) 2D *δ*‐MnO_2_

Similar to 1D TuMOs, 2D *δ*‐MnO_2_ often possess stabilizing ions and water molecules within the interlayer region, providing the possibility of ion insertion and extraction.^[^ ^34^, ^146^ ^]^

**Figure 7:**
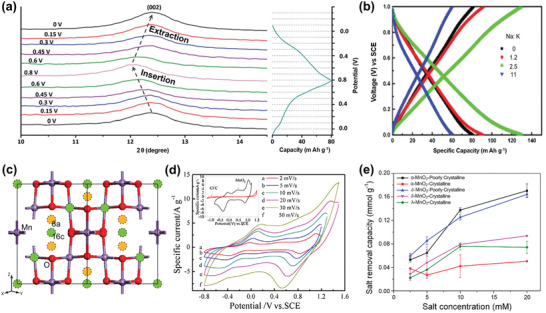
a) Ex situ XRD patterns of Na‐birnessite during a charge–discharge process at 0.5 C in 1 m Na_2_SO_4_ solution. Reproduced with permission.^[^ ^135^ ^]^ Copyright 2016, The Royal Society of Chemistry. b) Charge/discharge curves of *δ*‐MnO_2_ with different ratios of Na: K at a rate of 1.5 C in 1 Na_2_SO_4_ solution. Reproduced with permission.^[^ ^154^ ^]^ Copyright 2017, Springer Nature. c) Unit cell of the *λ*‐MnO_2_, in which orange and olive circles represent the 8a tetrahedral and 16c octahedral sites, respectively. Reproduced with permission.^[^ ^155^ ^]^ Copyright 2018, American Chemical Society. d) Cyclic voltammogram (CV) curves of *λ*‐MnO_2_ electrode at various scan rates in 1 m Na_2_SO_4_ solution. Reproduced with permission.^[^ ^156^ ^]^ Copyright 2014, Elsevier. e) Salt removal capacity against salt concentration of different MnO_2_ electrodes. Reproduced with permission.^[^ ^103^ ^]^ Copyright 2018, American Chemical Society.

Studies of *δ*‐MnO_2_ demonstrate that both water molecules and stabilizing ions could influence the capacity for Na^+^ insertion/extraction. In one aspect, the ion storage properties of *δ*‐MnO_2_ are largely attributed to the interlayer structural water.^[^ ^135^, ^153^ ^]^ For instance, Zhang et al.^[^ ^135^ ^]^ investigated the Na^+^ insertion/extraction of the *δ*‐MnO_2_ with Na^+^ stabilizing ions (Na‐birnessite) electrode via ex situ XRD analysis, as shown in **Figure** **7**a. During the charge process, the (002) peak shifted from 12.43° to 12.04°, corresponding to a minor expansion of the interlayer distance from 7.12 to 7.34 Å. Then the (002) peak shifted back to 12.45° after discharge process. This symmetric peak shift implies that structural water molecules act as pillars to release the structural strain and preserve the layered structure. Hence, the Na‐birnessite electrode displayed a high charge capacity of 80 mA h g^−1^ at 1C without significant decay after 150 cycles in 1 m Na_2_SO_4_ solution, while the heat‐treated sample without structural water with a interlayer distance of 5.66 Å retained only 60% capacity under identical testing conditions.

In another aspect, synthesizing *δ*‐MnO_2_ with different stabilizing cations can also change the interlayer distance and influence the ion insertion capacity. For instance, Byles et al.^[^ ^128^ ^]^ investigated two *δ*‐MnO_2_ materials stabilized by Na^+^ (Na‐birnessite) and Mg^2+^ (Mg‐buserite) as Na^+^ Faradaic electrodes of HCDI for desalination in 15 × 10^−3^ m NaCl solution. They found that the Mg‐buserite with a larger interlayer distance of 9.70 Å compared to Na‐birnessite (7.17 Å) allows for a greater amount of Na^+^ insertion, thus showing relatively higher ion removal capacities (37.2 mg g^−1^) than Na‐birnessite (31.5 mg g^−1^). Moreover, Feng et al.^[^ ^154^ ^]^ synthesized a series of *δ*‐MnO_2_ with dual stabilizing Na^+^ and K^+^ ions. By tuning the mole ratio of Na: K, the 2.5 Na: K material delivered the highest capacity (≈134 mA h g^−1^) at 1.5C in 1 m Na_2_SO_4_ solution as shown in Figure 7b, much higher than the *δ*‐MnO_2_ with other Na: K ratios.

3) 3D *λ*‐MnO_2_

The Na^+^ insertion/extraction behavior of *λ*‐MnO_2_ has been investigated by some researchers. Some earlier work showed that the electrochemical sodiation of the *λ*‐MnO_2_ could generate topological deformations, resulting in a local phase transition from the spinel to O′3 layered structure. On the other hand, more recent studies^[^ ^155^, ^156^, ^157^ ^]^ reported that reversible Na^+^ insertion/extraction could be achieved by initially filling the interstitial 8a tetrahedral sites and 16c octahedral sites of *λ*‐MnO_2_ as displayed in Figure 7c. The CV curves in 1 m Na_2_SO_4_ solution (Figure 7d) show that there are two pairs of oxidation/reduction peaks emerged, which could be ascribed to the insertion/extraction of Na^+^ in the 8a and 16c sites of *λ*‐MnO_2_, respectively. Regarding the performance, Whitacre et al.^[^ ^157^ ^]^ reported that the *λ*‐MnO_2_ electrode delivered double the specific capacity (≈80 mA h g^−1^) over the extensively studied Na_0.44_MnO_2_ electrode in 1 m Na_2_SO_4_ solution.

We have introduced some different polymorphs of MnO_2_, which display varying electrochemical performance, and thus some work has focused on their comparisons. Brousse et al.^[^ ^158^ ^]^ first studied the electrochemical performance comparison among *λ*‐MnO_2_ and other polymorphs in 2006, revealing that *λ*‐MnO_2_ (3D) shows intermediate electrochemical performance between *δ*‐MnO_2_ (2D) and 1D MnO_2_ with small cavities, precisely in the following order: *α* > *δ* > *λ* > *γ* > *β*. Another systematic study found similar results with specific capacitance decreasing in the following order: *α* > *δ* > *λ* > *γ* > *β*.^[^ ^149^ ^]^ However, a study conducted by Ghodbane et al.^[^ ^151^ ^]^ drew a completely different conclusion. They found that *λ*‐MnO_2_ (3D) exhibited the highest electrochemical performance followed by *δ*‐MnO_2_ (2D), and finally the 1D MnO_2_. They ascribed the high performance of *λ*‐MnO_2_ to its 3D interconnected tunnel structure, which provides more pathways for Na^+^ diffusion. These results reveal that the electrochemical performance of MnO_2_‐based electrodes is also significantly correlated with the ionic conductivity, which depends significantly on the crystallographic microstructure. Very recently, Leong et al.^[^ ^103^ ^]^ investigated the desalination performances of various crystalline MnO_2_ polymorphs in an HCDI system. From the desalination result shown in Figure 7e, it can be seen that the ion removal capacity of crystalline MnO_2_‐based HCDI cells decreased in the order of *δ*‐MnO_2_ (2D) > *λ*‐MnO_2_ (3D) > *α*‐MnO_2_ (1D), while two poorly crystalline forms of *α*‐MnO_2_ and *δ*‐MnO_2_ delivered higher salt removal capacities of 0.17 and 0.16 mmol g^−1^ (9.93 and 9.35 mg g^−1^), respectively. This is rather different from the results in the cases above. The ion storage property of MnO_2_ with different polymorphs is highly variable from one study to another, even for the same crystalline phase of MnO_2_, thus further systematic research is needed to clarify this.

4) Amorphous MnO_2_

Apart from manganese oxides with unique crystalline structures, various amorphous manganese oxides have been applied as Faradaic electrodes for CDI as well.^[^ ^76^, ^108^, ^159^, ^160^, ^161^, ^162^ ^]^ For instance, Wu et al.^[^ ^76^ ^]^ employed an amorphous MnO_2_ as a Faradaic electrode for a HCDI cell, which exhibited an ion removal capacity of 14.9 mg g^−1^ in 500 mg L^−1^ NaCl solution and maintained 95.4% of the initial capacity after 350 cycles. Moreover, considering that the electronic conductivity of carbon materials (about 50 S cm^−1^)^[^ ^163^ ^]^ is much higher than that of manganese oxides (10^−7^–10^−3^ S cm^−1^),^[^ ^152^, ^164^ ^]^ carbon materials have been incorporated into manganese oxides to further improve their Faradaic charge‐transfer.^[^ ^108^, ^159^, ^160^, ^162^ ^]^ For example, Liu et al. successively developed a manganese dioxide/activated carbon (MnO_2_/AC) composite^[^ ^159^ ^]^ and manganese dioxide/carbon nanotube‐chitosan composite (MnO_2_/CNT‐CS)^[^ ^160^ ^]^ as Na^+^ insertion Faradaic electrodes for CDI. Compared to either the pure carbon or MnO_2_ electrode, the use of these two composites exhibited enhanced desalination performances of 9.3 mg g^−1^ at 1.0 V in a 10 × 10^−3^ m NaCl solution, and 6.01 mg g^−1^ at 1.0 V in 1 × 10^−3^ m NaCl solution, respectively. These enhancements can be attributed to the combination of two charge storage mechanisms, which includes the electrosorption on the carbonaceous surface and the Faradaic reaction of MnO_2_.

In summary, the various studies outlined above indicate that MnO_2_‐based electrodes hold immense potential for CDI application. However, the ion capture performance is highly variable from one study to another, and the precise relationship between the structural features and ion capture performance has still not been delineated. In addition, Mn dissolution is a another issue for MnO_2_ materials,^[^ ^135^, ^165^, ^166^ ^]^ although this can be partly relieved by incorporating the crystallized water inside the MnO_2_ structure to release the structure strain during Na^+^ insertion/extraction process.^[^ ^135^, ^167^ ^]^ Therefore, establishing a structure‐property relationship of MnO_2_ and addressing the issue of Mn dissolution are both of interest for desalination performance optimization.

##### Sodium Manganese Oxides

Sodium manganese oxides (Na*_x_*MnO_2_, abbreviated as NMO), such as Na_0.2_MnO_2_, Na_0.4_MnO_2_, Na_0.44_MnO_2_, Na_0.66_MnO_2_, Na_0.7_MnO_2_, and *α*‐/*β*‐NaMnO_2_ have been intensively investigated since first generalized by Parant et al. in 1971.^[^ ^168^ ^]^ These materials generally show tunnel structures for 0 < *x* ≤ 0.44, a mixed tunnel and layered structures for 0.44 < *x* ≤ 0.66, and a fully layered structure for 0.66 < *x* ≤ 1.^[^ ^126^, ^169^ ^]^ Among these materials, tunnel‐structured Na_0.4_MnO_2_ and Na_0.44_MnO_2_ have been reported as Faradaic electrodes for CDI application so far, which will be introduced as follows.

1) Na_0.4_MnO_2_ (or Na_2_Mn_5_O_10_)

**Figure 8:**
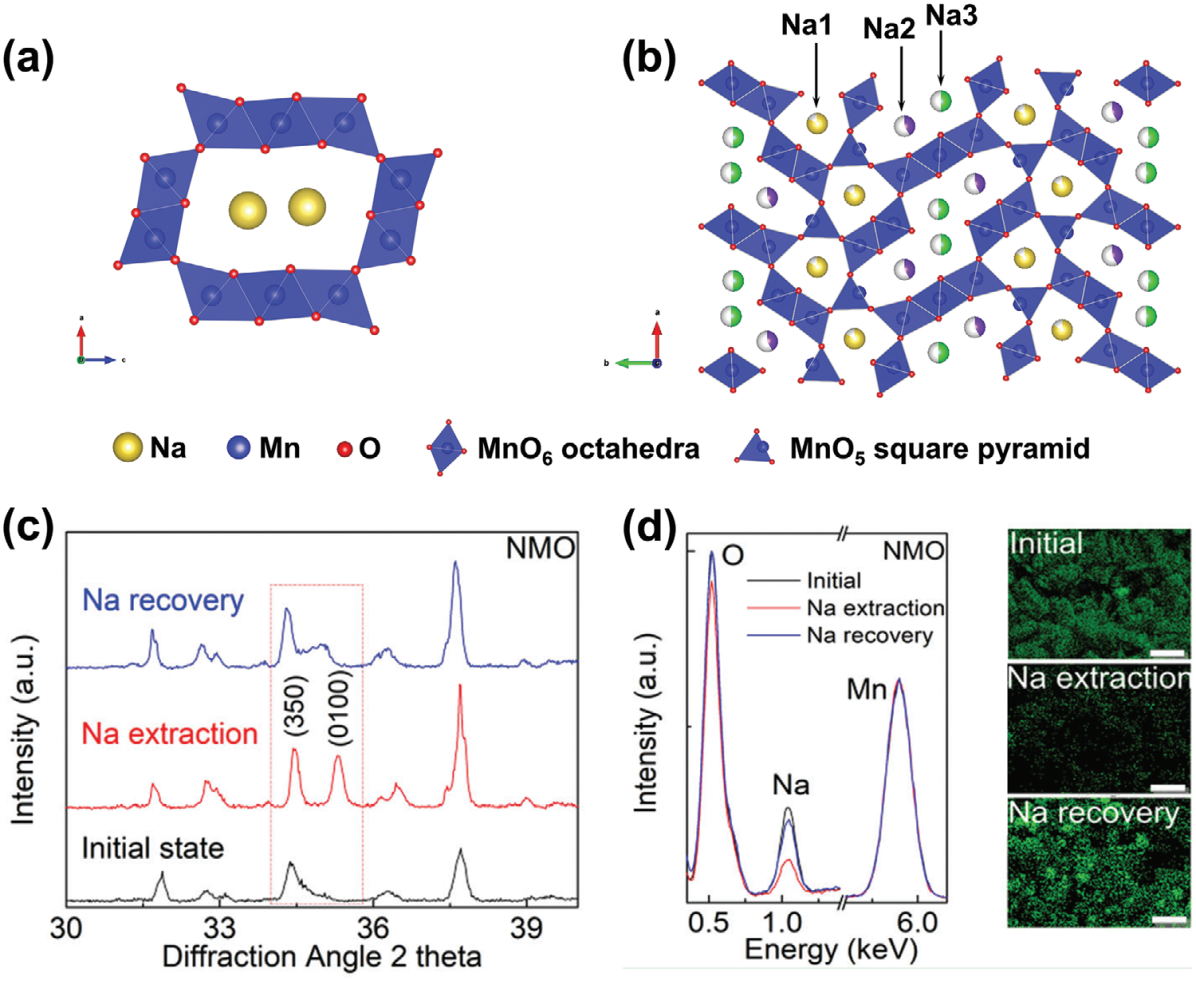
Crystal structure schematic of a) Na_0.4_MnO_2_ and b) Na_0.44_MnO_2_. c) XRD patterns and d) SEM‐EDX spectra of the initial Na_0.44_MnO_2_, its state of Na^+^ extraction, and state of Na^+^ recovery. (scale bar: 5 mm). Reproduced with permission.^[^ ^47^ ^]^ Copyright 2017, The Royal Society of Chemistry.

Na_0.4_MnO_2_, also referred to Na_2_Mn_5_O_10_, is isostructural to romanechite,^[^ ^109^, ^170^ ^]^ which consists of three MnO_6_ octahedra in length and two MnO_6_ octahedra in width with two equivalent stabilizing Na^+^ inside, as displayed in **Figure** **8**a. It is obvious that Na_0.4_MnO_2_ is a typical member (2 × 3) of the TuMOs family. Although the mechanism of Na^+^ insertion into Na_0.4_MnO_2_ is not fully understood and the exact site of Na^+^ within the tunnels remains unclear, at least 0.3 mol of Na^+^ could be reversibly extracted into/from the large open tunnel structure.^[^ ^169^ ^]^ Density functional theory (DFT) calculations imply that the energy barrier for Na diffusion along this rectangular channel is only 18 meV,^[^ ^171^ ^]^ which is much lower than that of S‐type tunnel structural Na_0.44_MnO_2_ (126–289 meV), indicating the ease of ion migration and storage.

Na_0.4_MnO_2_ was the first Na^+^ capture Faradaic electrode material to be introduced into water desalination by Pasta et al.^[^ ^39^ ^]^ in 2012. Coupled with an AgCl as Cl^−^ capture electrode, the as prepared Na_0.4_MnO_2_//AgCl cell showed an energy consumption of 0.29 Wh L^−1^ for 25% salt removal of seawater. Nevertheless, some additional phases like Na_2_Mn_3_O_7_, Mn_2_O_3_, and other electrochemically inert unknown impurities could be generated during the synthesis of Na_0.4_MnO_2_,^[^ ^39^, ^171^ ^]^ thus the annealing temperature of the precursor should be optimized to achieve the minimum impurity in the target product. Moreover, the structural characteristics and Na^+^ capture performance of this material need to be thoroughly investigated for further understanding.

2) Na_0.44_MnO_2_ (or Na_4_Mn_9_O_18_)

Na_0.44_MnO_2_, also referred to Na_4_Mn_9_O_18_, is another known tunnel‐structured Na^+^ insertion materials with a theoretical capacity of 121 mA h g^−1^.^[^ ^126^, ^172^ ^]^ It is isostructural with Na_4_Mn_4_Ti_5_O_18_,^[^ ^173^ ^]^ having an orthorhombic (Pbam space group) crystal symmetry.^[^ ^126^, ^168^ ^]^ The crystal structure contains two basic units: MnO_6_ octahedra in which sites have all Mn^4+^ ions and half of Mn^3+^ ions, while MnO_5_ square pyramid in which sites have half of Mn^3+^ ions. MnO_6_ octahedra and MnO_5_ square pyramid connect with each other by sharing their corners and edges, forming two types of tunnels as shown in Figure 8b. The Na1 sites in the small tunnel are almost fully filled, whereas the Na2 and Na3 sites in the large S‐shaped tunnels are only half occupied, which provides a fluent path for Na^+^ diffusion and thus contributes to capacity. Thus, Na_0.44_MnO_2_ have been widely applied as Na^+^ capture electrode in CDI systems.^[^ ^42^, ^46^, ^47^, ^49^, ^62^, ^112^ ^]^

Chen et al.^[^ ^47^ ^]^ reported a Faradaic CDI composed of Na_0.44_MnO_2_ as Na^+^ cathode and BiOCl as Cl^−^ anode. They investigated the electrochemical reaction mechanisms of Na_0.44_MnO_2_ and proposed its related redox reaction during Na^+^ insertion/extraction as shown below:

(11) $$\begin{array}{c} Na_{0.44-x}\mathrm{Mn}O_{2}+\mathrm{xN}a^{+}+xe^{-}↔Na_{0.44}\mathrm{Mn}O_{2} \end{array}$$

where the Na_0.44−_ *_x_*MnO_2_ with Na^+^ vacancies was transformed from the Na_0.44_MnO_2_ after pre‐charging and Na^+^ releasing. Besides, the evolution of crystal structure of Na_0.44_MnO_2_ during Na^+^ extraction/insertion processes had been elaborately tracked. As shown the zoomed‐in XRD patterns (Figure 8c), the red shift of peak (350) and its split into (350) and (0100) reveal the shrinking of lattice parameters upon sodium extraction, and the peaks shrink back after Na^+^ re‐insertion. Regarding chemical composition, as shown in the SEM‐EDX spectra (Figure 8d), the Na/Mn ratio decreases from 0.42 to 0.18, and recovers to 0.35, further demonstrating the reversible Na^+^ extraction/insertion process. With respect to the performance, the Na_0.44_MnO_2_//BiOCl cell achieved a high salt removal capacity of 68.5 mg g^−1^ after 50 cycles under a current density of 100 mA g^−1^ in 760 mg L^−1^ NaCl solution, and delivered an excellent charge efficiency of 0.958 in salt capture and 0.977 in salt release process. Another Faradaic CDI also reported by Chen et al.^[^ ^112^ ^]^ was consisting of Na_0.44_MnO_2_ and AgCl, which also delivered a high salt removal capacity of 57.4 mg g^−1^ after 100 cycles in 890 mg L^−1^ NaCl solution. In addition, Yoon et al.^[^ ^8^ ^]^ employed Na_0.44_MnO_2_ as Na^+^ capture electrode in a HCDI system, which showed a salt removal capacity of 31.2 mg g^−1^ and a maximum ion removal rate of 0.065 mg g^−1^ in 10 × 10^−3^ m NaCl solution. In a word, the present CDI cells with Na_0.44_MnO_2_ indicate good desalination performance, however, it is noticeable that the Mn^3+^ ions at the MnO_5_ sites of Na_0.44_MnO_2_ cannot be oxidized to Mn^4+^ ions,^[^ ^174^ ^]^ and thus about 20% of the Na^+^ cannot be extracted from the framework structure. The intrinsic capacity limitation of this material remains a challenge for its application in CDI.

#### 3.1.2 Titanium Oxide Compounds

Titanium oxide compounds, including titanium dioxide with various polymorphs, sodium titanate, and lithium titanate, are a group of Faradaic electrodes that have been widely investigated for CDI applications. These titanium oxide compounds are electrochemically featured by low redox potential based on the Ti^4+^/Ti^3+^ redox for reversible Na^+^ insertion/extraction. More details are introduced below.

##### Titanium Dioxide

**Figure 9:**
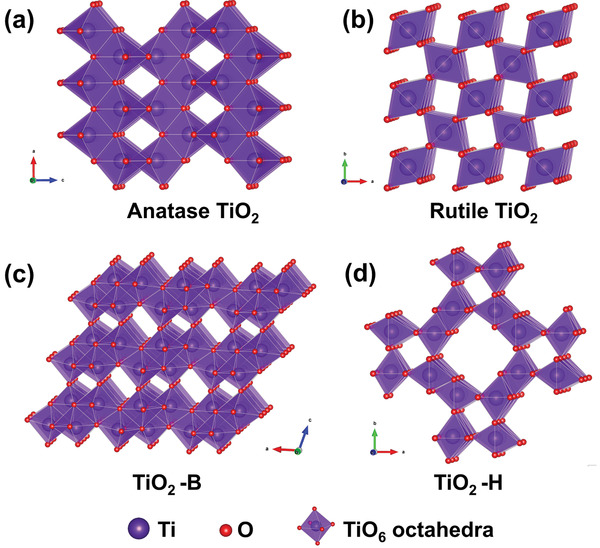
Crystal structure schematic of titanium oxides: a) anatase TiO_2_, b) rutile TiO_2_, c) TiO_2_‐B, and d) TiO_2_‐H.

Titanium dioxide (TiO_2_) exhibits versatile polymorphs according to the spatial arrangement of the TiO_6_ octahedra,^[^ ^175^ ^]^ with Ti^4+^ bonded to six equivalent O^2−^. Common TiO_2_ polymorphs include anatase TiO_2_, rutile TiO_2_, TiO_2_‐B, and TiO_2_‐H. As shown in Figure 9, i) anatase TiO_2_ belongs to the tetragonal I41/amd space group, in which TiO_6_ octahedra link with each other by sharing four edges, leading to a 3D structure with a distorted cubic dense‐stacked oxygen lattice (**Figure** **9**a). ii) Rutile TiO_2_ presents a tetragonal P42/mnm structure, comprised of corner‐sharing TiO_6_ octahedra (Figure 9b), which is considered as the most thermodynamically stable phase of TiO_2_.^[^ ^176^ ^]^ iii) TiO_2_‐B exhibits a monoclinic C2/m structure, consisting of both edge‐ and corner‐sharing TiO_6_ octahedra, forming a relatively wide framework compared to anatase and rutile structures (Figure 9c). (iv) TiO_2_‐H has a tetragonal I4/m structure, in which two chains of edge‐sharing TiO_6_ octahedra are connected together at their corners to create 2 × 2 channels, resulting in a more open structure than the above described polymorphs (Figure 9d). Among them, anatase TiO_2_ and rutile TiO_2_ have been extensively investigated as Faradic electrode for CDI application.

There are many researches about anatase TiO_2_ in CDI.^[^ ^119^, ^120^, ^127^, ^177^, ^178^, ^179^ ^]^ To enhance the desalination performance of TiO_2_, some shortcomings including the low electronic conductivity and sluggish ion diffusion should be relieved. In this regard, several studies have fabricated nanosized TiO_2_^[^ ^177^, ^179^ ^]^ and combined TiO_2_ with carbon‐based substrates, such as hollow carbon fiber,^[^ ^179^ ^]^ activated carbon,^[^ ^178^ ^]^ graphene,^[^ ^177^ ^]^ and multiwalled carbon nanotubes.^[^ ^120^ ^]^ Ramadan et al.^[^ ^120^ ^]^ reported a TiO_2_ nanotubes/multiwalled carbon nanotubes composite electrode that delivers a salt removal capacity of 13.2 mg g^−1^ in 50 mg L^−1^ NaCl solution. Barakat et al.^[^ ^119^ ^]^ fabricated a hybrid network electrode consisting of TiO_2_ nanofibers and activated carbon, achieving a salt removal capacity of 17 mg g^−1^ and an excellent salt removal efficiency of 89.6% in 292 mg L^−1^ NaCl solution.

**Figure 10:**
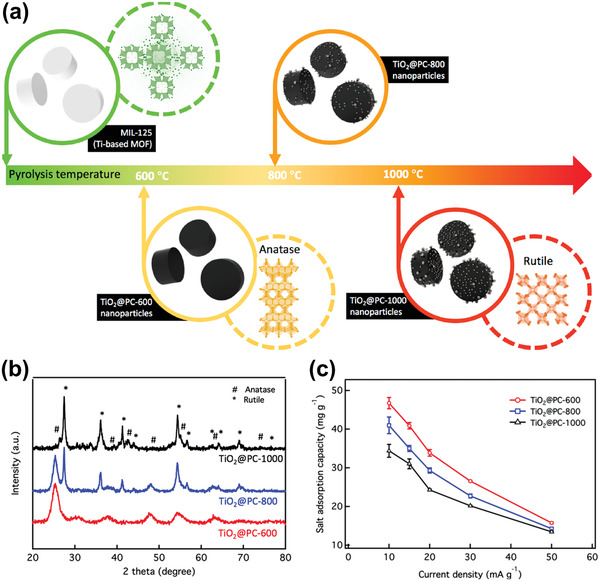
a) Schematic illustration of the synthesis process of different TiO_2_@PC samples. b) XRD patterns and c) salt removal capacity versus current densities of TiO_2_@PC samples (The number 600, 800, and 1000 denote the annealing temperatures). Reproduced with permission.^[^ ^127^ ^]^ Copyright 2019, American Chemical Society.

In addition to the study of anatase TiO_2_ with carbon composites, rutile TiO_2_ synthesized as nanotubes with carbon imbedded was also tested for CDI application,^[^ ^117^ ^]^ which exhibited a salt removal capacity of 13.11 mg g^−1^ and an excellent salt removal efficiency of 89.6% at 500 mg L^−1^ NaCl solution. The TiO_2_ polymorphs with different open structures tend to exhibit various electrochemical performance that influences the desalination results. DFT study^[^ ^180^ ^]^ shows that anatase TiO_2_ forms 2D ion diffusion pathways along both a‐ and b‐axes, with a relatively lower sodiation energy barrier, while rutile TiO_2_ provides only one ion diffusion pathway along c‐axis. Moreover, a comparative experiment was conducted by Ding et al.^[^ ^127^ ^]^ By adjusting the annealing temperature, a series of TiO_2_@porous carbon (TiO_2_@PC) composites with different phases (anatase, rutile, and their mixed phases) were obtained as shown in **Figure** **10**a,b. The salt removal capacity was enhanced for composites produced with a lower thermal treatment temperature as compared in Figure 10c. Thus, the anatase TiO_2_@PC‐600 (the number denotes the annealing temperature) electrode demonstrated a higher salt removal capacity (46.7 mg g^−1^) compared to the mixed‐phase TiO_2_@PC‐800 electrode (41 mg g^−1^) and rutile TiO_2_@PC‐1000 electrode (34.4 mg g^−1^) at 10 mA g^−1^ in 1000 mg L^−1^ NaCl solution, which further reveals the facile Na^+^ migration in anatase compared to the rutile phase.

Besides these two typical TiO_2_ phases, other polymorphs, such as TiO_2_‐B and TiO_2_‐H that have wider and more open frameworks can potentially be applied as Faradic electrodes for CDI. In addition, the relationship among the polymorphs, ion insertion, diffusion paths, and desalination properties needs to be studied further.

##### Sodium Titanates

Sodium titanates, characterized by their high ionic exchange ability and low sodium insertion potential, and large theoretical capacity (>200 mA h g^−1^).^[^ ^107^, ^181^ ^]^ Sodium titanates mainly exist as either of the following series:^[^ ^181^ ^]^ Na_2_Ti*_n_*O_2_ *_n_*_+1_ (*n* = 1–9) or Na_4_Ti_n_O_2_ *_n_*_+2_ (*n* = 1, 3, 5, or 9). Most of these sodium titanates exhibit a layered structure. Among them, Na_2_Ti_3_O_7_ and Na_4_Ti_9_O_20_ have been investigated as Faradaic electrode to capture Na^+^ in CDI.

1) Na_2_Ti_3_O_7_

**Figure 11:**
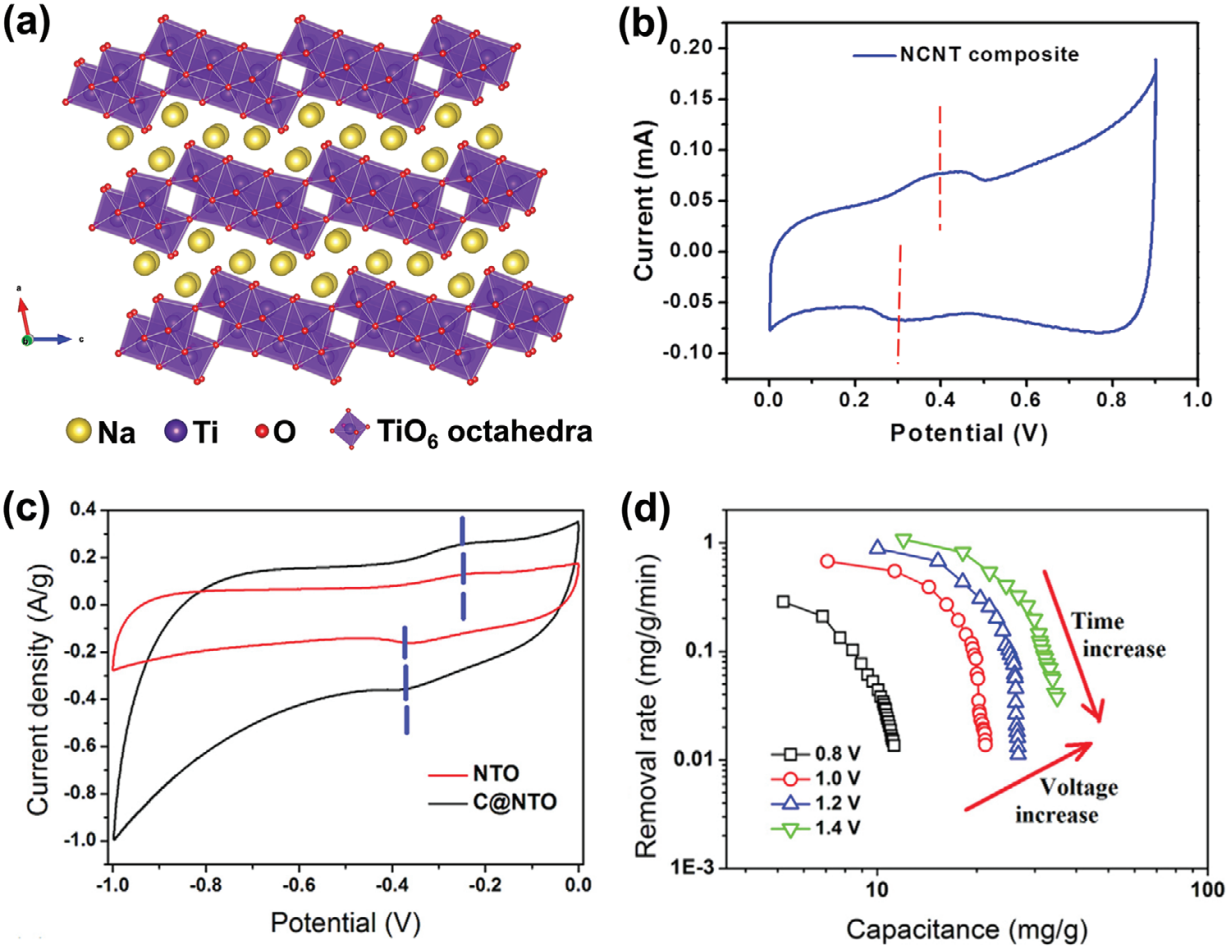
a) Crystal structure schematic of Na_2_Ti_3_O_7_. b) CV curves in a voltage range of 0.0–0.9 V for NCNT electrode at 1 mV s^−1^ in 3 m NaCl solution. Reproduced with permission.^[^ ^102^ ^]^ Copyright 2019, The Royal Society of Chemistry. c) CV curves of NTO and C@NTO electrodes at 5 mV s^−1^ in 1 m NaCl solution. d) CDI Ragone plots of C@NTO//AC HCDI at various operational voltages. Reproduced with permission.^[^ ^107^ ^]^ Copyright 2019, Elsevier.

Na_2_Ti_3_O_7_ is a member of the Na_2_Ti*_n_*O_2_ *_n_*_+1_ (*n* = 3) series. As shown in **Figure** **11**a, the structure is composed of zig‐zag shaped [Ti_3_O_7_]^2−^ layers with edge‐sharing triple TiO_6_ octahedral chains, while Na^+^ occupies the interlayer positions between these sheets.^[^ ^129^ ^]^ This architecture creates accessible tunnels along the b axis, where Na^+^ diffusion can occur. To be specific, a Na_2_Ti_3_O_7_–CNT (NCNT) composite supported on reduced graphene oxide has been explored as a binder‐free HCDI electrode.^[^ ^102^ ^]^ As shown in Figure 11b, the redox peak at 0.3 and 0.4 V corresponds to the Na^+^ insertion/extraction based on the Ti^4+^/T^i3+^ redox, as shown in the following Equation (12):

(12) $$\begin{array}{c} Na_{2}Ti_{3}O_{7}+\left(x-2\right)Na^{+}+\left(x-2\right)e^{-}↔Na_{x}Ti_{3}O_{7} \end{array}$$

Regarding the desalination performance, a remarkable salt removal capacity of 129 mg g^−1^ at 3000 mg L^−1^ salt concentration can be achieved with low energy consumption of 0.4 W h g^−1^.

2) Na_4_Ti_9_O_20_

Na_4_Ti_9_O_20_ is the most typical Na_4_Ti_n_O_2n+2_ (n = 9) with a layered structure consisting of TiO_6_ octahedral chains, although the exact crystal structure is difficult to determine due to its low crystallinity.^[^ ^182^, ^183^ ^]^ Yue et al.^[^ ^107^ ^]^ prepared carbon@Na_4_Ti_9_O_20_ (C@NTO) core‐shell nanotube composites as electrodes for CDI application. As displayed in Figure 11c, two distinct peaks (−0.22 and −0.38 V) confirm the reaction of NTO with Na^+^ in solution, which correspond to the Ti^4+^/Ti^3+^ redox in accordance with the following equation

(13) $$\begin{array}{c} Na_{4}Ti_{9}O_{20}+9Na^{+}+9e^{-}↔Na_{13}Ti_{9}O_{20} \end{array}$$

Thus, the C@NTO‐based HCDI cell provided a high desalination capacity of 80.56 mg g^−1^ at 1.4 V as shown in Figure 11d. Another case in point is a reduced graphene oxide@Na_4_Ti_9_O_20_ composite,^[^ ^106^ ^]^ which was synthesized through a hydrothermal process and also delivered a good desalination capacity of 42 mg g^−1^ in a HCDI system.

##### Spinel Li_4_Ti_5_O_12_

**Figure 12:**
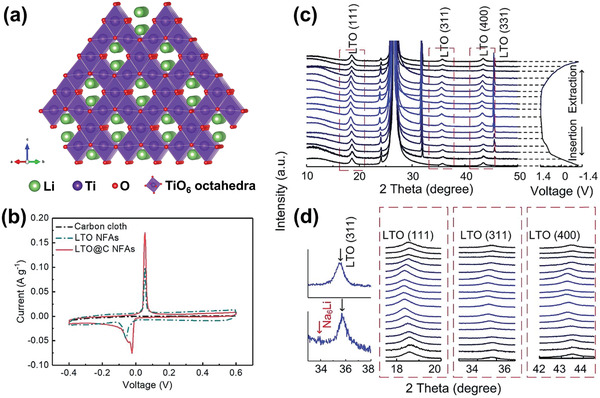
a) Crystal structure schematic of spinel Li_4_Ti_5_O_12_. b) CV curves of carbon cloth, LTO NFA, and LTO@C NFA electrodes at 5 mV s^−1^, c) ex situ XRD patterns of the LTO@C NFAs obtained during the Na^+^ insertion/extraction stage in 1 m NaCl solution, and d) enlarged versions for the marked XRD patterns in (c). Reproduced with permission.^[^ ^189^ ^]^ Copyright 2019, The Royal Society of Chemistry.

Spinel Li_4_Ti_5_O_12_ (LTO) belongs to the Fd$\bar{3}$m space group, which is built up with TiO_6_ octahedra sharing edges with other adjacent TiO_6_ octahedra, forming a 3D framework structure with rhombic long tunnels to accommodate lithium ions as displayed in **Figure** **12**a. Characterized by its remarkable electrochemical properties and zero strain feature,^[^ ^184^ ^]^ LTO has been extensively studied in both the lithium and sodium ion batteries.^[^ ^185^, ^186^, ^187^, ^188^ ^]^ Very recently, Guo et al.^[^ ^189^ ^]^ investigated the ion insertion mechanism and desalination performance of a Li_4_Ti_5_O_12_ nanoflake array with carbon shell (LTO@C NFA) electrode in a HCDI system. A good salt removal capacity of 25 mg g^−1^ over 30 cycles in 2500 mg L^−1^ NaCl solution was achieved. The CV redox peaks at −0.02 and 0.06 V as shown in Figure 12b correspond to the following ion insertion mechanism:

(14) $$\begin{array}{c} 2Li_{4}Ti_{5}O_{12}+6Na^{+}+6e^{-}\to Li_{7}Ti_{5}O_{12}+Na_{6}\mathrm{LiT}i_{5}O_{12} \end{array}$$

which involves three phases during the reversible insertion/extraction process. The Faradaic insertion mechanism was further verified by ex situ XRD analysis as shown in Figure 12c,d. It has been proven that during the Na^+^ insertion process, the Na^+^ ions will occupy the 16c sites instead of the tetrahedral 8a sites of Li_4_Ti_5_O_12_, and simultaneously the Li^+^ ions at 8a sites will be driven to the 16c sites by coulombic repulsion.^[^ ^188^, ^190^ ^]^ A new peak at 33.8° is emerged after Na^+^ insertion, as revealed in Figure 12d, corresponding to the Na_6_LiTi_5_O_12_(Na6Li) phase. In addition, the peaks of (111), (311) and (400) corresponding to Li_4_Ti_5_O_12_ shift to smaller angles, demonstrating the transformation of Li_4_Ti_5_O_12_ to Li_7_Ti_5_O_12_ (Li7Ti) phase. During the Na^+^ extraction process, Na^+^ ions as well as Li^+^ ions are extracted from the 16c sites, and simultaneously the vacancies left at the 8a sites will be re‐filled by the extracted Li^+^ ions to re‐form the Li_4_Ti_5_O_12_ phase. As revealed in Figure 12d, the peaks of the Li7Ti phase shifts back to the initial Li_4_Ti_5_O_12_ position, showcasing the high reversibility of the ion insertion mechanism. However, the large size of Na^+^ and the volume expansion of the Na6Li phase cause sluggish electrochemical kinetics of the Li_4_Ti_5_O_12_ electrode for water desalination via CDI.

#### 3.1.3 Vanadium Oxide Compounds

In recent years, vanadium oxide compounds with layered structure possess advantages of large interlayer distance, satisfied theoretical capacity, and have shown thier excellent capacity for reversible Na^+^ insertion/extraction.^[^ ^130^, ^191^ ^]^ Among them, the representative layered vanadium oxide *α*‐V_2_O_5_^[^ ^123^ ^]^ and the representative sodium vanadium oxides Na_1.1_V_3_O_7.9_^[^ ^192^ ^]^ have been investigated as Na^+^ capture cathodes in CDI applications.

##### *α*‐V_2_O_5_

**Figure 13:**
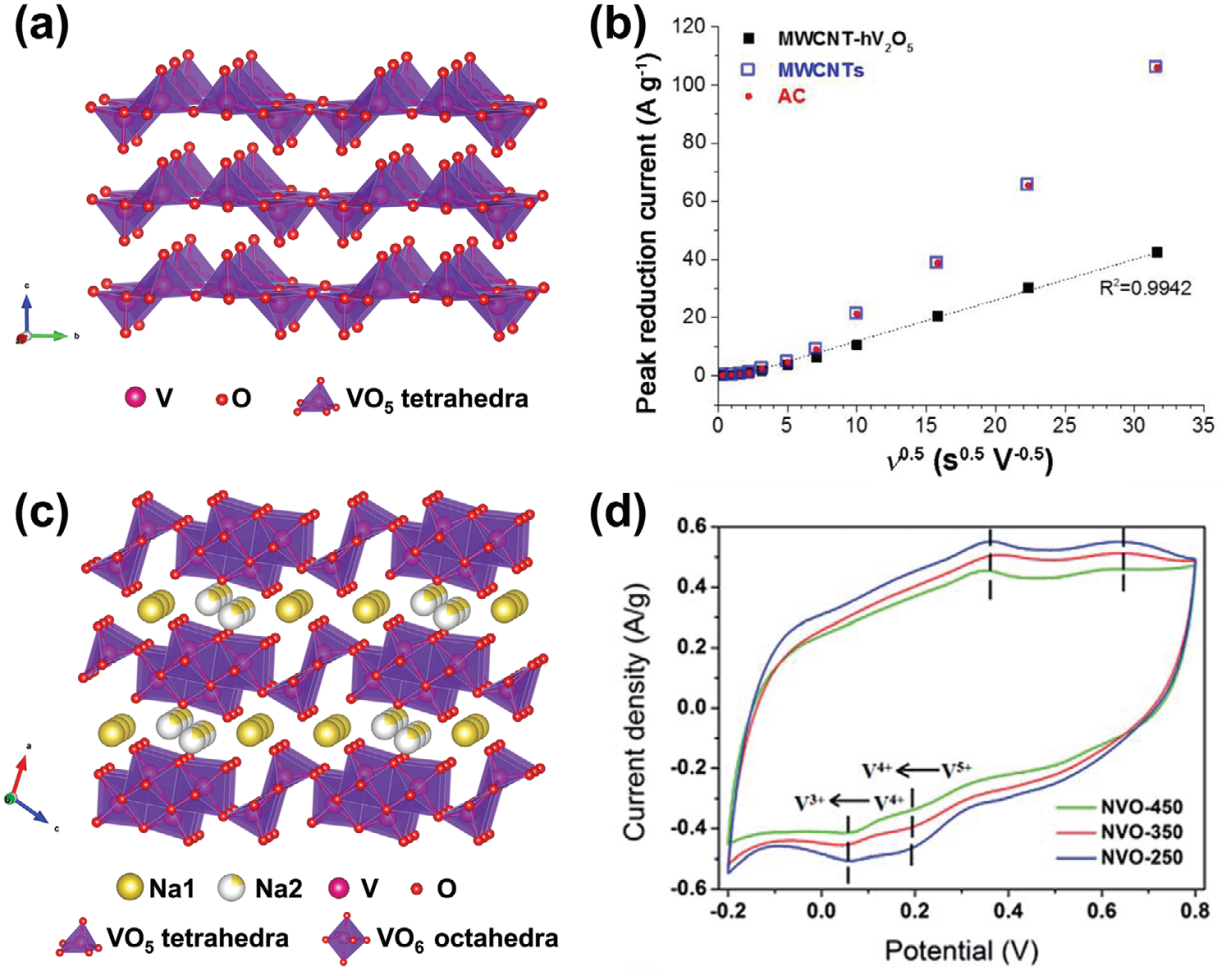
a) Crystal structure schematic of *α*‐V_2_O_5_. b) The peak reduction current (at 0.2 V vs Ag/AgCl) versus the square route of scan rate (*ν* ^0.5^) in 1 m NaCl solution. Reproduced with permission.^[^ ^123^ ^]^ Copyright 2017, WILEY‐VCH. c) Crystal structure schematic of Na_1.1_V_3_O_7.9_. d) CV curves of as prepared NVO samples at 5 mV s^−1^ in 1 m NaCl solution. Reproduced with permission.^[^ ^192^ ^]^ Copyright 2019, The Royal Society of Chemistry.

*α*‐V_2_O_5_ is one of the most extensively studied metal oxides in the vanadium oxide group since it is the most stable phase with the highest oxidation state of vanadium.^[^ ^193^ ^]^ As shown in **Figure** **13**a, orthorhombic *α*‐V_2_O_5_ possesses a typical layered structure with a space group of Pmmn (*a* = 11.52 Å, *b* = 3.57 Å, and *c* = 4.37 Å), where layers consist of alternating pairs of VO_5_ square pyramids sharing corners and edges. These layers stack together through weak van der Waals forces along the [001] crystallographic direction. Such a unique structure allows V_2_O_5_ to accommodate guest cations (e.g., Li^+^, Na^+^ and K^+^) in six‐coordinated sites between the layers in accordance with the following equation

(15) $$\begin{array}{c} V_{2}O_{5}+\mathrm{xN}a^{+}+xe^{-}↔Na_{x}V_{2}O_{5} \end{array}$$

In addition, the multiple oxidation states of vanadium (V^5+^, V^4+^ and V^3+^) with high theoretical capacity render it an attractive electrode material for alkali metal ion batteries and CDI applications.^[^ ^123^, ^191^, ^194^, ^195^ ^]^

Given that low intrinsic electronic conductivity (≈1 × 10^−6^ S cm^−1^)^[^ ^196^ ^]^ of V_2_O_5_ leads to sluggish ion diffusion behavior, similar to the strategy mentioned above, the formation of composites with high conductive carbon can mitigate the poor conductivity. For example, an intertwined network composed of V_2_O_5_ nanowires and carbon nanotubes achieves an enhanced electrical conductivity (≈3.0 S cm^−1^).^[^ ^191^ ^]^ Recently, Lee and Presser^[^ ^123^ ^]^ electrochemically deposited V_2_O_5_ on multiwalled carbon nanotube (MWCNT) to synthesize a MWCNT‐V_2_O_5_ as Faradaic electrode for water desalination. Figure 13b plots the peak reduction current as a function of the square route of scan rate (*ν* ^0.5^). The data obtained for AC and MWCNT electrodes have an exponential trend, whereas the MWCNT–V_2_O_5_ fits well to a linear function (R^2^ = 0.9942), implying a charge storage mechanism which followed the Randles‐Sevcik equation (*i*∝*ν* ^0.5^) typical for redox insertion with vanadium pentoxides.^[^ ^197^, ^198^ ^]^ Regarding the performance, the corresponding MWCNT‐V_2_O_5_//AC cell delivered an appreciable salt removal capacity of 23.6 mg g^−1^ in 600 × 10^−3^ m NaCl solution. However, the compact interlayer distance (4.37 Å) of *α*‐V_2_O_5_ restricts the insertion/extraction and diffusion of ions. Therefore, strategies such as reconstructing the V‐O polyhedral^[^ ^199^, ^200^ ^]^ should be developed to enlarge the interlayer distance of *α*‐V_2_O_5_ and increase its desalination performance.

##### Na_1.1_V_3_O_7.9_

Sodium vanadium oxides are envisioned as good candidates for Na^+^ insertion due to their large interlayer distance, abundant reactive sites and huge specific capacity (>200 mA h g^−1^).^[^ ^130^, ^201^ ^]^ As a representative example of sodium vanadium oxides, Na_1.1_V_3_O_7.9_ presents a layered structure, as shown in Figure 13c. The layers are composed of alternate VO_6_ octahedra and VO_5_ tetrahedra linked by corner‐shared O atoms. The Na^+^ occupies in two independent positions. Na1 is located at the octahedral sites with full occupancy, Na2 is located at the tetrahedral sites with part occupancy. The larger interlayer distance (7.08 Å) compared to *α*‐V_2_O_5_ (4.37 Å) has been proved to be more favorable for Na^+^ insertion/extraction.^[^ ^202^ ^]^ Yue et al.^[^ ^192^ ^]^ synthesized a ribbon structured Na_1.1_V_3_O_7.9_@reduced graphene oxide (NVO@rGO) composite as Na^+^ capture cathode for a Faradaic CDI system. As shown in Figure 13d, all the CV curves shows two characteristic reduction peaks situated at 0.06 and 0.21 V, corresponding to the valence state changes of Na_1.1_V_3_O_7.9_ from V^5+^ to V^4+^ and V^4+^ to V^3+^ respectively, which are due to the insertion of Na^+^. The oxidation peaks are situated at 0.35 and 0.62 V, accompanied by the de‐intercalation of Na^+^. The relevant behavior is depicted as the following equation

(16) $$\begin{array}{c} Na_{1.1}V_{3}O_{7.9}+\mathrm{xN}a^{+}+xe^{-}↔Na_{1.1+x}V_{3}O_{7.9} \end{array}$$

Coupled with Ag@rGO as Cl^−^ capture anode, the assembled NVO@rGO//Ag@rGO cell delivered a desalination capacity of 39.9 mg g^−1^ and desalination rate of 0.037 mg g^−1^ min^−1^ at 1.4 V in 250 mg L^−1^ NaCl solution, and even achieved a higher desalination capacity of 82.2 mg g^−1^ in 2000 mg L^−1^ NaCl solution.

In addition to the manganese/titanium/vanadium oxide compounds, some other metal oxides have also been used as Na^+^ capture cathodes for CDI system. For example, Ma et al.^[^ ^203^ ^]^ electrodeposited RuO_2_ onto activated carbon (RuO_2_‐AC) as cathode to remove Na^+^ by the redox reactions of Ru^4+^, and the corresponding HCDI cell showed a desalination capacity of 11.26 mg g^−1^ at 1.2 V in 5 × 10^−3^ m NaCl solution. A rGO/Co_3_O_4_ electrode fabricated by Divyapriya et al.^[^ ^204^ ^]^ presented a reversible sodination/desodination reaction, and the corresponding HCDI cell showed a desalination capacity of 20.21 mg g^−1^ at 1.6 V in 500 mg L^−1^ NaCl solution. The ion insertion mechanism and charge transfer of these metal oxide compounds still need to be further explored, and thus guide their structural design to further strengthen the corresponding desalination performances.

#### 3.1.4 Polyanionic‐Type Compounds

**Figure 14:**
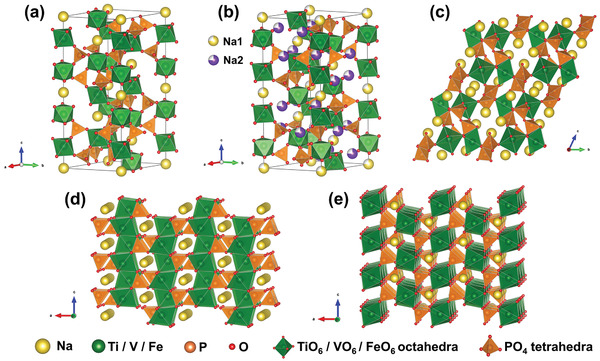
Crystal structure schematic of a) NaTi_2_(PO_4_)_3_, b) Na_3_V_2_(PO_4_)_3_, c) Na_2_FeP_2_O_7_, and NaFePO_4_ in d) olivine phase and e) maricite phase.

Polyanionic‐type compounds are a promising group of as Na^+^ insertion materials due to their diverse morphology and structure, excellent thermal and oxidative stability, and the ability to adjust the specific redox voltage along with the local circumstances of polyanions.^[^ ^205^, ^206^ ^]^ The polyanionic open framework provide appreciable channels for Na^+^ diffusion and migration. In this section, we review several popular polyanionic‐type compounds used as electrodes for CDI application, including the sodium superionic conductor NASICON‐type phosphates (NaTi_2_(PO_4_)_3_ and Na_3_V_2_(PO_4_)_3_), sodium ferric pyrophosphate (Na_2_FeP_2_O_7_), and ferric phosphate (FePO_4_). All of these structures are displayed in **Figure** **14**.

##### NASICON‐Type Phosphates

NASICON‐type materials are well known for remarkable ionic conductivity and structural stability.^[^ ^132^, ^207^ ^]^ Their chemical formula can be expressed as Na*_x_*M_2_(XO_4_)_3_ (M is Ti, Fe, V, or Nb etc.; X is P, S, Mo, W, or As etc.; *x* is 0–4), with the structure consisting of corner‐shared MO_6_ octahedra and XO_4_ tetrahedra, creating prominent Na^+^ diffusion paths. To date, NASICON‐type phosphates NaTi_2_(PO_4_)_3_ and Na_3_V_2_(PO_4_)_3_ have been intensively studied for CDI application.^[^ ^41^, ^77^, ^94^, ^95^, ^116^ ^]^

1) NaTi_2_(PO_4_)_3_

NaTi_2_(PO_4_)_3_ (NTP) offers an open 3D framework structure as shown in Figure 14a, in which TiO_6_ octahedra groups are connected with PO_4_ tetrahedra by corner‐shared oxygen atoms, forming large spacious interstices.^[^ ^208^ ^]^ NTP exhibits a high theoretical capacity of 133 mA h g^−1^ based on the redox reaction of Ti^3+^/Ti^4+^.^[^ ^209^ ^]^

**Figure 15:**
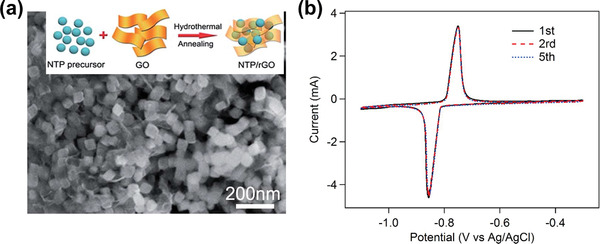
a) SEM image and Synthesis process of NTP/rGO nanocomposite. b) CV curves of NTP/rGO electrode at 0.2 mV s^−1^ in 1 m NaCl solution. Reproduced with permission.^[^ ^116^ ^]^ Copyright 2017, The Royal Society of Chemistry.

The insertion/extraction activity of Na^+^ in pristine NTP was explored,^[^ ^210^ ^]^ where a large difference in the large charge/discharge voltage plateaus was observed, which indicates significant polarization, partially due to its poor electronic conductivity. To solve this problem, a typical strategy involves forming composites with highly conductive carbon materials. For instance, Huang et al.^[^ ^116^ ^]^ prepared a NTP/rGO nanocomposite by combining NTP precursor with graphene oxide (GO) through a hydrothermal method followed by annealing treatment, as shown in **Figure** **15**a. Moreover, the CV curves of NTP/rGO electrode in Figure 15b shows a pair of well‐defined redox peak at −0.75/−0.85 V, which corresponds to the redox reaction of Ti^4+^/Ti^3+^ via a two‐phase mechanism according to the equation below

(17) $$\begin{array}{c} {\mathrm{NaTi}}_{2}{\left({\mathrm{PO}}_{4}\right)}_{3}+2{\mathrm{Na}}^{+}+2e^{-}↔{\mathrm{Na}}_{3}{\mathrm{Ti}}_{2}{\left({\mathrm{PO}}_{4}\right)}_{3} \end{array}$$

Regarding to the desalination performance, the NTP/rGO//AC HCDI achieved a high salt removal capacity of 140 mg g^−1^, and remained 86% of the capacity after 100 cycles in 1000 mg L^−1^ NaCl feed solution, whereas the pure NTP only showed an initial capacity of 112 mg g^−1^ with retention of 63%. They also designed a Faradaic CDI consisting of NaTi_2_(PO_4_)_3_/reduced graphene oxide (NTP/rGO) and Ag /reduced graphene oxide, which exhibited a reversible desalination capacity of 105 mg g^−1^ after 50 cycles in 2500 ppm NaCl solution.^[^ ^95^ ^]^ Most recently, Wang et al.^[^ ^77^ ^]^ prepared a metal–organic‐framework derived NaTi_2_(PO_4_)_3_ with carbon (NTP/C) hybrid, and the assembled NTP/C//AC HCDI achieved a remarkable electrochemical performance with a super‐high salt removal capacity of 167.4 mg g^−1^ and a salt removal rate of 11 mg g^−1^ min^−1^ in 1000 mg L^−1^ NaCl solution.

2) Na_3_V_2_(PO_4_)_3_

**Figure 16:**
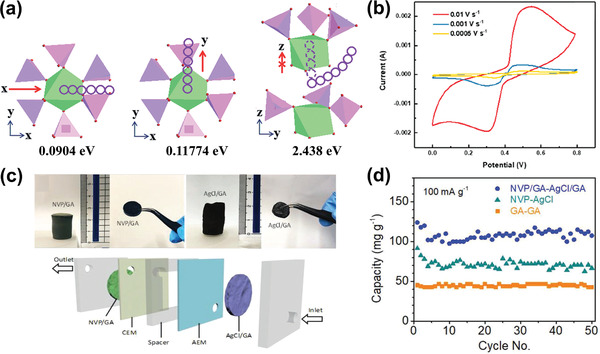
a) Possible Na^+^ migration paths within Na_3_V_2_(PO_4_)_3_ along the *x*, *y*, and curved *z* directions. Reproduced with permission.^[^ ^214^ ^]^ Copyright 2014, The Royal Society of Chemistry. b) CV curves of NVP@C electrode at different scan rates. Reproduced with permission.^[^ ^41^ ^]^ Copyright 2019, American Chemical Society. c) Photos of NVP/GA and AgCl/GA and the schematic of the NVP/GA//AgCl/GA Faradaic CDI cell, and d) cycling performance of the NVP/GA//AgCl/GA cell with a current density of 100 mA g^−1^ in 1000 mg L^−1^ NaCl solution. Reproduced with permission.^[^ ^94^ ^]^ Copyright 2019, WILEY‐VCH.

Na_3_V_2_(PO_4_)_3_ (NVP) is another attractive NASICON material that can be used for CDI electrodes with a theoretical capacity of 117 mA h g^−1^ based on its V^4+^/V^3+^ redox couple.^[^ ^132^, ^211^ ^]^ The rhombohedral NVP structure as shown in Figure 14b belongs to the R3c space group with a 3D framework built of [V_2_(PO_4_)_3_] units, which consist of corner‐sharing VO_6_ octahedra and PO_4_ tetrahedra units.^[^ ^212^ ^]^ In this polyhedral framework, Na^+^ locates at two different sites. Na1 is occupied in [V_2_(PO_4_)_3_] units along the c direction with coordinates of 6b (0, 0, 0), while Na2 is distributed analogously but along b direction in 8‐coordinate sites, whose coordinates can be denoted as 18e (*x*, 0, 1/4).The Na2 sites with 2/3 occupancy are easier to achieve Na^+^ extraction than Na1 sites with 1 occupancy because of the weaker bonding of Na‐O.^[^ ^213^ ^]^ According to first‐principles calculations and related experiments, the potential Na^+^ migration mechanism is depicted in **Figure** **16**a.^[^ ^214^ ^]^ There are two pathways along *x* and *y* axes with comparatively low migration energies and one possible curved path with a slightly higher migration energy for ion migration, which implies a 3D transport characteristic of NVP.

Similar to NTP, NVP also suffers from low electronic conductivity. Most reports on NVP for CDI application adopt a conductive carbon support or coating. Cao et al.^[^ ^41^ ^]^ synthesized Na_3_V_2_(PO_4_)_3_@C (NVP@C) via a sol‐gel process. The CV curves of NVP@C at various scan rates display a pair of redox peaks at +0.53/+0.32 V as shown in Figure 16b, which correspond to the redox reaction presented below:

(18) $$\begin{array}{c} Na_{3}V_{2}{\left(PO_{4}\right)}_{3}↔Na_{3-x}V_{2}{\left(PO_{4}\right)}_{3}+\mathrm{xN}a^{+}+xe^{-} \end{array}$$

The assembled NVP@C//AC HCDI delivered an outstanding salt removal capacity of 137.30 mg NaCl g^−1^ NVP@C, salt removal rate of 0.076 mg NaCl g^−1^ NVP@C s^−1^, and a low energy consumption of 2.16 kg‐NaCl kWh^−1^ in 100 × 10^−3^ m NaCl solution. Zhao et al.^[^ ^115^ ^]^ further investigated the Na^+^ insertion/extraction in NVP by XRD, in which the characteristic peaks of NVP red shift after Na^+^ extraction and shift back to initial positions after re‐insertion, indicating the high reversibility of Na^+^ insertion/extraction. Moreover, Zhao et al.^[^ ^94^ ^]^ fabricated a binder‐free deionization system with a free‐standing Na_3_V_2_(PO_4_)_3_/rGO aerogel (NVP/GA) as the Na^+^ capture Faradaic electrode and a free‐standing AgCl/rGO aerogel (AgCl/GA) as the Cl^−^ capture Faradaic electrode, as shown in Figure 16c. Due to the 3D open porous structure of aerogel and highly conductive graphene, the desalination capacity of the system reached 107.5 mg g^−1^ after 50 cycles at 100 mA g^−1^ in 1000 mg L^−1^ NaCl solution as shown in Figure 16d.

Although NASICON‐type phosphates have high ionic conductivity and coupling to carbon improves the electronic conductivity, more studies are needed to optimize the electrochemical performance of NASICON‐type phosphates using strategies, such as regulating the particle size and doping with other metals, which are effective in enhancing the intrinsic electronic conductivity in sodium ion batteries.

##### Na_2_FeP_2_O_7_

**Figure 17:**
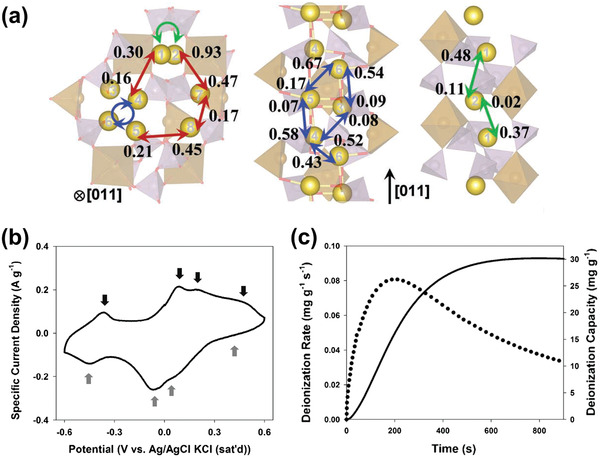
a) Calculated Na^+^ migration barriers (in eV) for the NFP, in which the red arrows represent 2D migration paths, while green and blue arrows represent the outward channels. Reproduced with permission.^[^ ^216^ ^]^ Copyright 2013, WILEY‐VCH. b) The CV of NFP based on the Ag/AgCl KCl saturated electrode in 2 m NaCl solution at 0.5 mV s^−1^ and c) salt removal capacity and corresponding rate of the NFP//AC HCDI cell during the charge step. Reproduced with permission.^[^ ^111^ ^]^ Copyright 2016, Elsevier.

Sodium ferric pyrophosphate (Na_2_FeP_2_O_7_; abbreviated as NFP) is a representative Na‐based pyrophosphate. As shown in Figure 14c, NFP has a the triclinic structure (P1), comprised of corner‐sharing FeO_6_−FeO_6_ units, which are linked with PO_4_−PO_4_ [P_2_O_7_] groups by edge and corner‐sharing, forming 3D crooked paths for Na^+^ migration.^[^ ^215^ ^]^ NFP displays a theoretical capacity of 97 mAh g^−1^ involving a Na_2_Fe^II^P_2_O_7_/NaFe^III^P_2_O_7_ (desodiated state) redox transformation.^[^ ^215^, ^216^ ^]^ DFT calculations along with synchrotron X‐ray diffraction (SXRD) patterns suggests that 8 Na crystallographic sites are occupied in the unit cells.^[^ ^216^ ^]^ As shown in **Figure** **17**a, the migration barrier of Na1 extraction (0.48 eV) along the [011] direction is lower than the Na2‐Na8 extraction channels (0.54–0.67 eV), indicating that Na1 is the most easily accessible site for insertion/extraction.

Kim et al.^[^ ^111^ ^]^ implemented NFP electrodes into a HCDI system. In the CV curves showed in Figure 17b, the characteristic peaks refer to the extraction and insertion of Na^+^ ions at −0.36, 0.07, 0.18, and 0.43 V (marked by black arrows) and −0.45, −0.07, 0.06, and 0.41 V (marked by gray arrows) respectively. The corresponding HCDI system as shown in Figure 17c exhibited a high rate capability of 0.081 mg g^−1^ s^−1^ and a salt removal capacity of 30.2 mg g^−1^. Since the reactivity of <1 Na^+^ insertion/extraction per stoichiometric unit of NFP leads to unsatisfactory desalination capacity, future studies should focus on enhancing ion insertion by optimizing the structure.

##### FePO_4_

Ferric phosphate (FePO_4_) has been considered a feasible host insertion material for Na^+^ through the following reaction formula:^[^ ^101^, ^217^, ^218^ ^]^

(19) $$\begin{array}{c} \mathrm{FeP}O_{4}+Na^{+}+e^{-}\to \mathrm{NaFeP}O_{4} \end{array}$$

The corresponding sodium ferric phosphate (NaFePO_4_) structure has two crystalline phases:^[^ ^219^ ^]^ olivine and maricite as illustrated in Figure 14d,e. In olivine NaFePO_4_, [FeO_6_] octahedral groups are linked with [PO_4_] tetrahedra sharing corners and edges, forming 1D Na^+^ migration paths parallel to the b direction. However, olivine structure is not very thermodynamically stable, and is generally prepared from olivine LiFePO_4_ using cumbersome ion exchange methods,^[^ ^220^ ^]^ thus limiting its application. Unfortunately, the thermally stable maricite NaFePO_4_ displays no electrochemical activity as it lacks available channels for Na^+^ migration.^[^ ^221^ ^]^ On the other hand, amorphous FePO_4_ can be synthesized via a facile chemical precipitation process or structural transformation, and has a high theoretic capacity of 178 mA h g^−1^.^[^ ^217^ ^]^ Its isotropy with short‐range ordering can ensure reliable and continuous ions diffusion pathways, resulting in enhanced redox kinetic performance and structural stability.^[^ ^222^ ^]^

**Figure 18:**
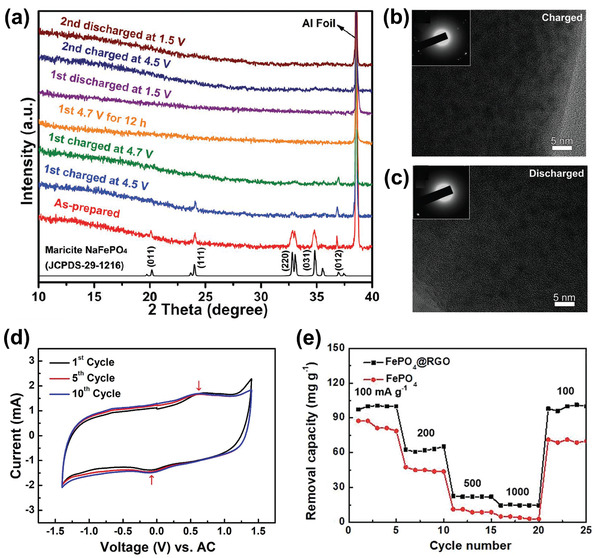
a) Ex situ XRD patterns of NaFePO_4_@C at different charged/discharged stages during the first two cycles and HRTEM images (insets: SAED patterns) of the NaFePO_4_@C at fully b) charged (4.7 V for 12 h) and c) discharged (1.5 V) states in the first cycle. Reproduced with permission.^[^ ^223^ ^]^ Copyright 2018, WILEY‐VCH. d) CV curve of the FePO_4_ electrode measured at 10 mV s^−1^ paired with an AC electrode 1 m NaCl solution and e) rate performance of FePO_4_//AC CDI cell and FePO_4_@RGO//AC CDI cell. Reproduced with permission.^[^ ^101^ ^]^ Copyright 2018, The Royal Society of Chemistry.

In general, an amorphous phase forms after complete desodiation of maricite NaFePO_4_.^[^ ^223^, ^224^ ^]^ A case in point is shown in **Figure** **18**a, where characteristic peaks of maricite NaFePO_4_ gradually decreases with charging until it disappears completely. During the discharge process, Na^+^ re‐insert into the structure with no detected characteristic peaks, demonstrating that the amorphous phase can be well maintained. And the HRTEM images as presented in Figure 18b,c also showcase the amorphous phase in both desodiation and sodiation products. Moreover, the amorphous NaFePO_4_ displayed an impressive electrochemical performance with a high capacity of 145 mA h g^−1^. Recently, Guo et al.^[^ ^101^ ^]^ prepared an amorphous FePO_4_ supported with reduced graphene oxide (FePO_4_@RGO) by chemical precipitation. As shown in Figure 18d, the pair of redox peaks located at 0.60 and 0.02 V imply the existence of the insertion mechanism. The fabricated HCDI cell delivered a decent salt removal capacity of 100 mg g^−1^ at 100 mA g^−1^ and a superior rate performance of 0.117 mg g^−1^ s^−1^ at 1000 mA g^−1^ as shown in Figure 18e. Although amorphous NaFePO_4_ shows improved structural stability and redox kinetics than the crystalline phases, while the deeper studies about the Na^+^ insertion/extraction mechanism is still insufficient, thus more related works are needed in further research.

#### 3.1.5 Metal Hexacyanometalates

**Figure 19:**
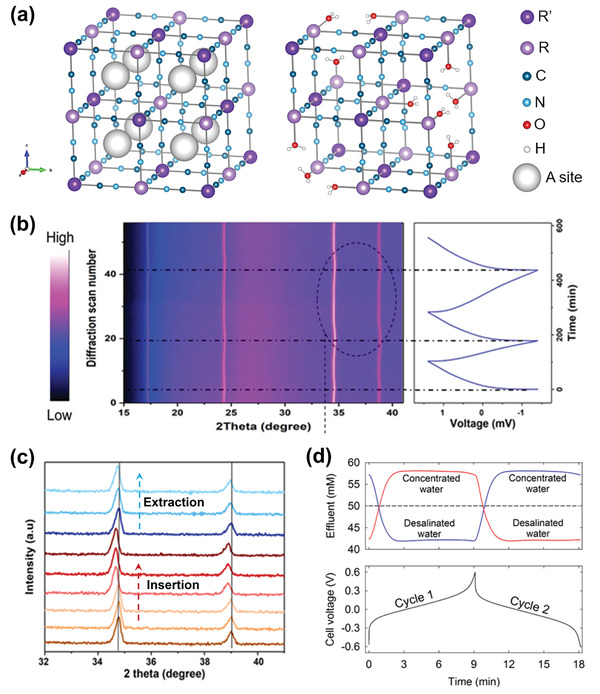
a) Crystal structure schematic of metal hexacyanometalates: nominal perfect metal hexacyanometalate framework without structural defects (left, *x* = 2) and defective metal hexacyanometalate framework with [R′(CN)_6_] vacancies (right, *x* = 0). b) In situ XRD patterns of FeFe(CN)_6_/rGA electrode at 30 mA g^−1^ during charge and discharge processes within −1.4 to 1.4 V and c) the selected XRD patterns for the highlighted oval. Reproduced with permission.^[^ ^229^ ^]^ Copyright 2019, American Chemical Society. d) Representative effluent concentration and cell voltage profiles of CuHCF//CuHCF CDI cell. Reproduced with permission.^[^ ^52^ ^]^ Copyright 2017, American Chemical Society.

Metal hexacyanometalates (MHCF), also denoted as Prussian blue analogues,^[^ ^133^, ^225^, ^226^ ^]^ have a formula of A*_x_*R[R′(CN)_6_] (A = Na, K; R and R′ = Fe, Co, Ni, Mn; *x* = 0–2) and possess a face‐centered cubic structure. **Figure** **19**a (left) shows a schematic illustration of a metal hexacyanometalate with a fully occupied A site (*x* = 2). Low‐spin R′(III) is located at the site coordinated to C of the C≡N ligand, while high‐spin R (II) is coordinated to N of the C≡N ligand, generating a 3D polymeric framework with adequate interstitial spaces. Cations and zeolitic water molecules occupy the body‐centered A sites of the subcell. The large channels along the [001] directions allow for fast insertion/extraction of various cations, such as Na^+^, K^+^, Ca^2+^, and Mg^2+^, etc., which are the major alkali metal ions in seawater.^[^ ^227^, ^228^ ^]^

In addition, metal hexacyanometalates are nontoxic, inexpensive, and insoluble in water. Hence, metal hexacyanometalates are considered as promising candidates for CDI electrodes.^[^ ^53^, ^59^, ^230^ ^]^ Since metal hexacyanometalates are generally synthesized through low‐temperature solution‐phase methods, the prepared products tends to contain crystal water occupying [R′(CN)_6_] vacancies as shown in Figure 19a (right), forming a defective compound that can be expressed as A*_x_*R[R′(CN)_6_]_1−_ *_y_*·nH_2_O (0 < *y* < 1). The water content varies with the preparation conditions, but it is difficult to achieve completely final dehydrated states due to the rapid kinetics of the precipitation process. The presence of crystal water molecules increases the molecular weight of the crystal materials and reduce the number of available interstitial A sites for Na ions,^[^ ^231^ ^]^ limiting the overall capacity of the material.

The electrochemical properties of metal hexacyanometalates for Na^+^ insertion/extraction within an aqueous environment have been widely explored. In the A*_x_*R[R′(CN)_6_], R′ site is generally occupied by low‐spin Fe(III) and R is generally divalent transition metal element such as Fe(II), Cu, or Ni. Thus, take Na*_x_*Cu[Fe(CN)_6_] as an example, the insertion/extraction processes can be shown in the following redox reaction:

(20) $$\begin{array}{ccc} & & \mathrm{NaC}u^{\mathrm{II}}\left[Fe^{\mathrm{III}}{\left(\mathrm{CN}\right)}_{6}\right]+\mathrm{xN}a^{+}+xe^{-}\to \\ & & \;Na_{1+x}Cu^{\mathrm{II}}{\left[Fe^{\mathrm{II}}{\left(\mathrm{CN}\right)}_{6}]{}_{x}[Fe^{\mathrm{III}}{\left(\mathrm{CN}\right)}_{6}\right]}_{1-x} \end{array}$$

During the insertion/extraction of a Na^+^ into/from the interstitial site, the Fe(III) undergoes a corresponding Fe(III)/Fe(II) redox transformation, ensuring the electroneutral environment for the lattice. When R and R′ are Fe(II) and Fe(III) respectively, the corresponding theoretical capacity of the single crystal FeFe(CN)_6_ is 180 mA h g^−1^.^[^ ^124^ ^]^

Guo et al.^[^ ^124^ ^]^ synthesized a FeFe(CN)_6_@nanopore‐reduced graphene oxide (FeFe(CN)_6_@NPG) composite as the electrode of CDI, and the corresponding FeFe(CN)_6_@NPG//AC CDI reached a high capacity of 120.0 mg g^−1^ and a salt removal rate of 0.5430 mg g^−1^ s^−1^. Choi et al.^[^ ^40^ ^]^ constructed a HCDI cell consisting of a K_0.03_Cu[Fe(CN)_6_]_0.65_·0.43H_2_O electrode and an AC electrode, which exhibited an “IEM‐like” effect with higher salt removal capacity of 23.2 mg g^−1^ and salt removal rate of 0.24 mg g^−1^ s^−1^ than these of a traditional symmetric cell (6.9 mg g^−1^ and 0.05 mg g^−1^ s^−1^, respectively).

To further study the ion capture mechanism, Vafakhah et al.^[^ ^229^ ^]^ conducted in situ XRD measurement for a FeFe(CN)_6_/reduced graphene oxide aerogel (FeFe(CN)_6_/rGA) electrode, which provides insights into the real‐time lattice changes during the charge–discharge process, as shown in Figure 19b. The selected range of XRD pattern from Figure 19b is magnified and specified in Figure 19c, in which the characteristic peaks of FeFe(CN)_6_ for (400) and (420) crystal planes shift to lower angles during the charge process, illustrating the insertion of Na^+^ into the host framework and the enlargement of the lattice parameters. Meanwhile, these characteristic peaks return to the initial position during the discharge process, indicating the high reversibility of the electrode. Moreover, the developed FeFe(CN)_6_/rGA//rGA HCDI cell exhibited a remarkable salt removal capacity of 130 mg g^−1^ at 100 mA g^−1^.

Furthermore, based on metal hexacyanometalates, researchers developed the novel NID cell^[^ ^49^, ^50^, ^51^, ^52^ ^]^ composed of two Na^+^ capture Faradaic electrodes in two channels that are separated by an AEM. For instance, A CuHCF//CuHCF CDI cell constructed by Kim et al.^[^ ^52^ ^]^ produced both desalinated and concentrated water simultaneously in two separate channels during both the charge and discharge steps as shown in Figure 19d. The maximum desalination capacity was up to 100 mg g^−1^ in an aqueous 50 × 10^−3^ m NaCl solution with an energy consumption of only 0.02 kWh m^−3^. Another novel NID cell is proposed by Lee et al.,^[^ ^50^ ^]^ who reported a CDI cell consisting of NaNiHCF and NaFeHCF asymmetric electrodes, which exhibited a salt removal capacity of 59.9 mg g^−1^ at 40% ion removal efficiency with a low energy consumption of 0.34 Wh L^−1^. To further improve the desalination performance, Zhao et al.^[^ ^53^ ^]^ designed a core@shell heterostructured CuHCF@NiHCF. Attributed to the high‐capacity CuHCF core and the stable NiHCF shell, the corresponding CuHCF@NiHCF//CuHCF@NiHCF cell delivered a higher desalination capacity (71.8 mg g^−1^) and improved capacity retention (92% after 50 cycles) than the sole core material‐based CuFe PBA//CuFe PBA CDI (54.3 mg g^−1^; 74% after 50 cycles).

In brief, the 3D framework structure of metal hexacyanometalates allows rapid Na^+^ insertion/extraction, thus various material compositions and composite architectures are widely explored. Currently, high salt removal capacity and low energy consumption make metal hexacyanometalates attractive materials for CDI electrodes. Moreover, their performance can be further improved through some other strategies, such as decreasing crystallized water in the structures of metal hexacyanometalates, which still remains a challenge and need further investigations.

### 3.2 Cl^−^ Capture Faradaic Electrode Materials

According to current research and development status, some traditional methods for Cl^−^ capture have remaining technical challenges. For instance, in the typical chemical method (i.e., the oxidation of Cl^−^ to Cl_2_ gas), the produced Cl_2_ is highly toxic and the oxidation requires a high voltage of 1.36 V, which is higher than that of water electrolysis (1.2 V).^[^ ^232^, ^233^ ^]^ Another traditional method involves physical separation (i.e., salt separation through filtration or evaporation (e.g., RO and MSF)), which is limited by high energy consumption. In comparison, the use of specific electrode materials that can selectively intercalate or react with Cl^−^ in an aqueous system using a facile CDI system is more promising, as it is more eco‐friendly and with lower energy consumption. However, identifying acceptable Cl^−^ storage electrode materials which simultaneously possess high capacity, stability, insolubility, and reversibility within a limited voltage range (between H_2_ and O_2_ evolution) still remains a challenge.

To date, there are only a few materials have been used as Cl^−^ capture CDI electrodes. Specifically, Ag^[^ ^39^, ^42^, ^62^, ^94^, ^95^, ^112^ ^]^ and Bi^[^ ^43^, ^47^ ^]^ are the two mostly commonly reported Cl^−^ storage materials, which both operate via a conversion reaction. Additionally, some studies have reported that calcined LDH^[^ ^57^, ^63^, ^79^, ^80^ ^]^ can also be used for Cl^−^ capture via ion insertion. We will detailly introduce these three electrode materials in detail.

#### 3.2.1 Ag/AgCl

Ag/AgCl is the first^[^ ^38^ ^]^ and by far the most common materials^[^ ^78^, ^82^, ^94^, ^95^, ^112^, ^115^ ^]^ explored for Cl^−^ removal. Ag has a high theoretic capacity (248 mA h g^−1^)^[^ ^95^, ^234^, ^235^ ^]^ for Cl^−^ capture according to the charge transfer reaction:

(21) $$\begin{array}{c} \mathrm{Ag}+Cl^{-}⇆\mathrm{AgCl}+e^{-} \end{array}$$

**Figure 20:**
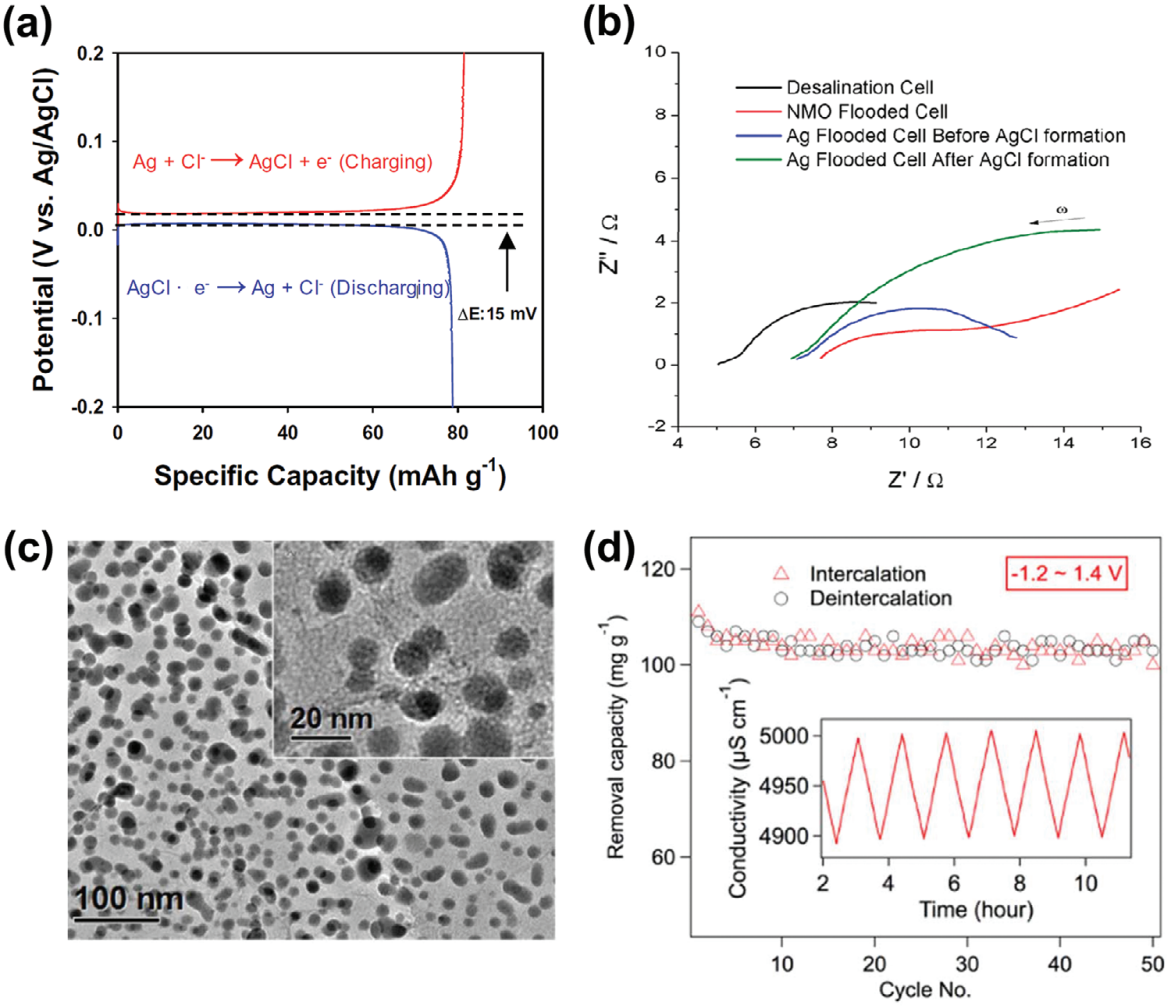
a) Galvanostatic charge/discharge curves of the Ag/AgCl electrode at 1 mA cm^−2^ in 1 m NaCl solution. Reproduced with permission.^[^ ^82^ ^]^ Copyright 2020, Elsevier B.V. b) Nyquist plots of Ag electrode before (blue curve) and after (green curve) AgCl formation in a seawater electrolyte. Reproduced with permission.^[^ ^39^ ^]^ Copyright 2012, American Chemical Society. c) TEM images of AgNPs/rGO and d) salt removal capacity of NTP/rGO//AgNPs/rGO CDI cell during insertion and extraction progress of 50 cycles at 100 mA g^−1^, inset is the curves of conductivity change as a function of time. Reproduced with permission.^[^ ^95^ ^]^ Copyright 2019, Elsevier.

The wide potential stability and corrosion resistance make Ag/AgCl favorable toward Cl^−^ capture. Yoon et al.^[^ ^82^ ^]^ investigated the Cl^−^ capture process of Ag/AgCl electrode. As shown in **Figure** **20**a, each voltage profile showed one constant voltage plateau, demonstrating the two‐phase conversion reaction. Furthermore, the small gap between the oxidation and reduction potential of 15 mV allowed Ag/AgCl system to operate at a low cell voltage of 0.2 V. The assembled anion desalination cell consisting of Ag/AgCl electrodes combined with a CEM exhibited a desalination capacity of 85 mg g^−1^ at 1 mA cm^−2^ in 500 × 10^−3^ m NaCl solution.

Notably, AgCl is an insulator, which distinctly increases the impedance of the cell system as shown in Figure 20b. Thereby, some measures have been adopted to relieve the kinetic limitation induced by AgCl, such as designing Ag/AgCl nanoparticles and combining Ag/AgCl with highly conductive materials. In addition, Huang et al.^[^ ^95^ ^]^ fabricated Ag nanoparticles/reduced graphene‐oxide (AgNPs/rGO) through a one‐pot synthesis process. The nanosized Ag and the integration with graphene as shown in Figure 20c, which can effectively facilitated the chloride ion reaction between electrode and electrolyte and further improve the conductivity. Coupled with NaTi_2_(PO_4_)_3_/reduced graphene‐oxide (NTP/rGO) as the Na^+^ capture Faradaic electrode, the dual‐ions CDI exhibits a reversible desalination capacity of 105 mg g^−1^ after 50 cycles in 2500 ppm NaCl solution as displayed in Figure 20d.

However, the excessive cost of silver and its dissolution in seawater severely hinders its practical application.^[^ ^42^ ^]^ In seawater, a series of soluble Ag chloride complexes exit in equilibrium via a related reversible chemical reaction. Since the Cl^−^ concentration is as high as 600 × 10^−3^ m, the equilibrium concentration of soluble silver can reach up to 8.9 ppm, nearly 100 times the U.S. EPA secondary drinking water criteria of 0.1 ppm. The dissolution of silver is not only a stability issue, but can also causes serious health defects such as argyria and kidney damage.^[^ ^236^ ^]^ In addition, the 150% volume expansion of Ag to AgCl and the 40% volume shrinkage in the reverse reaction can pulverize the electrode and decrease the cycling performance,^[^ ^237^ ^]^ which must be addressed.

#### 3.2.2 Bi/BiOCl

**Figure 21:**
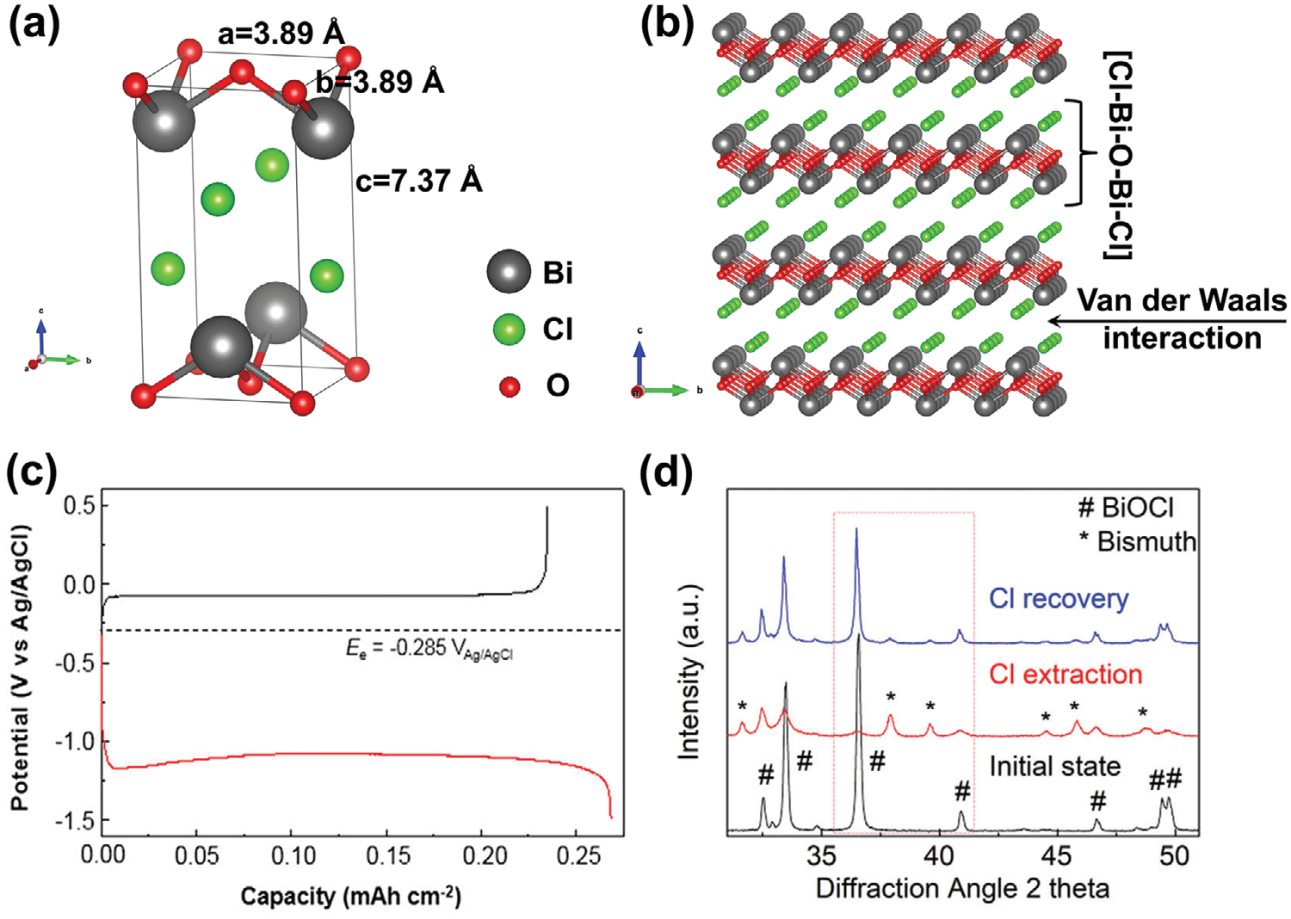
Crystal structure schematic of BiOCl: a) a unit cell with lattice constants and b) a larger field of vision with stacked [Cl–Bi–O–Bi–Cl] sheets. c) Potential curves of Bi electrode during the galvanostatic oxidation process (black) and reduction process (red) at ±1 mA cm^−2^ in 0.6 m NaCl solution. Reproduced with permission.^[^ ^43^ ^]^ Copyright 2017, American Chemical Society. d) XRD patterns of the initial BiOCl electrode, its state after Cl extraction, and state after Cl recovery. Reproduced with permission.^[^ ^47^ ^]^ Copyright 2017, The Royal Society of Chemistry.

Bismuth (Bi) is another viable Cl^−^ removal electrode material that is more cost effective than Ag. Bi was demonstrated to capture Cl^−^ via oxidation to BiOCl in 2017 for the first time.^[^ ^43^, ^47^ ^]^ BiOCl presents a tetragonal layered structure with lattice constants of *a* = *b* = 3.890 Å and *c* = 7.370 Å^[^ ^238^, ^239^, ^240^ ^]^ as shown in **Figure** **21**a. In an expanded view as shown in Figure 21b, we can see that BiOCl consists of [Cl–Bi–O–Bi–Cl] sheets stacked together via weak van der Waals interactions between the Cl atoms along the c‐axis.^[^ ^238^, ^239^, ^240^ ^]^ The [110] direction is the fastest growth direction during the forming process, while the [001] direction is the slowest because of weak c‐axis bonding, resulting in the common flake‐like morphology of BiOCl.

Nam et al.^[^ ^43^ ^]^ reported that Bi captures Cl^−^ by the reversible chemical reaction of Bi + Cl^−^ + H_2_O ⇆ BiOCl+ 2H^+^+3e^−^, which presents the conversion between Bi and BiOCl with corresponding oxidation and reduction plateau (−0.08 and −1.27 V) as shown in Figure 21c. This BiOCl system has a theoretical charge capacity of 103 mA h g^−1^.^[^ ^43^, ^68^ ^]^ Whereas Chen et al.^[^ ^47^ ^]^ presented a different opinion, in which the chloride capture electrode occurs the reversible reaction of 3BiOCl + 3e^−^ ⇆ Bi + Bi_2_O_3_ + 3Cl^−^. Instead of two existing substances of Bi and BiOCl, this reaction involves three substances of Bi, BiOCl, and additional Bi_2_O_3_. XRD was conducted to track the phase transformation of the electrode materials as shown in Figure 21d. It is found that only the BiOCl phase exists initially, and then the BiOCl should be supposed to convert into Bi and Bi_2_O_3_ after Cl^−^ extraction. However, the postulated Bi_2_O_3_ phase was not definitely detected, as only Bi peaks could be clearly observed. The reason proposed in this work and other related studies^[^ ^238^, ^241^, ^242^ ^]^ was that the Bi_2_O_3_ may exists in amorphous state, which seems unconvincing without any direct evidence and requires in‐depth exploration.

For seawater desalination via CDI, Nam et al.^[^ ^43^ ^]^ fabricated a Bi foam electrode with a high surface area nanocrystalline morphology to facilitate Cl^−^ diffusion into the Bi lattice, along with NaTi_2_(PO_4_)_3_ as the Na^+^ capture electrode. It was reported that ≈49% of the Bi in the electrode was electrochemically active, 82.96 mg of Cl^−^ in 600 × 10^−3^ m NaCl solution can be captured with per 1 g of Bi, almost 5 times than that of porous carbon electrode (16.38 mg of Cl^−^ per 1 g of carbon). The NaTi_2_(PO_4_)_3_//Bi CDI cell achieved the desalination/salination at ±1 mA cm^−2^ powered by a net voltage input of only 0.2 V.

Despite its high desalination performance, Bi/BiOCl as Cl^−^ capture electrode suffers certain drawbacks: (i) Sluggish reduction kinetics of BiOCl to Bi, this results in increased energy consumption; (ii) imbalance removal of Cl^−^ and Na^+^, the Cl^−^ capture behavior of Bi/BiOCl consumes three electrons per Cl^−^, while Na^+^ capture of many electrode materials as mentioned above just require one electron per Na^+^; (iii) high volume expansion of 158% accompanies the conversion of Bi and BiOCl. All of the above impede the development of the Bi/BiOCl electrode for CDI and require optimization in further research and development.

#### 3.2.3 Calcined Layered Double Hydroxides

**Figure 22:**
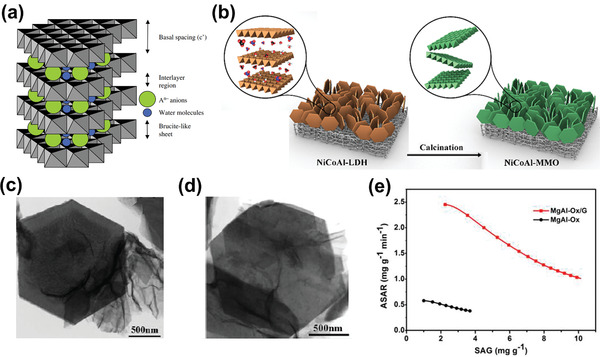
a) Schematic representation of the LDH structure. Reproduced with permission.^[^ ^243^ ^]^ Copyright 2008, Elsevier. b) Schematic illustration of the process of the calcination of NiCoAl‐LDHs to NiCoAl‐MMOs. Reproduced with permission.^[^ ^79^ ^]^ Copyright 2018, Elsevier. TEM images of c) MgAl‐LDHs/G, d) MgAl‐O*_x_*/G, and e) CDI Ragone plot of MgAl‐O*_x_*/G//AC‐HNO_3_ HCDI cell. Reproduced with permission.^[^ ^57^ ^]^ Copyright 2018, American Chemical Society.

Some studies also used calcined layered double hydroxides to capture CI^−^ ions.^[^ ^57^, ^63^, ^79^, ^80^ ^]^ Layered double hydroxides (LDHs), also referred as hydrotalcite‐like materials, are a class of layered materials consisting of positively charged layers and some interlayer charge‐balancing anions and water molecules^[^ ^243^ ^]^ as displayed in **Figure** **22**a. They can be expressed by the chemical formula [M_1−_ *_x_* ^II^M*_x_* ^III^(OH)_2_]*^x^* ^+^[(A^n−^)*_x_*_/n_]*^x^* ^−^·mH_2_O,^[^ ^243^, ^244^, ^245^ ^]^ in which M^II^ and M^III^ represent divalent and trivalent metal cations, A^n−^ denotes anion, *x* refers to the molar ratio of M^III^/(M^II^ + M^III^), and m represents the molar quantity of water molecule. With higher capacity from pseudocapacitance compared to capacitive carbon materials, LDHs have been widely used for supercapacitors.^[^ ^246^, ^247^, ^248^ ^]^ In addition, owing to their positive charged layers, large interlayer distance, and high ion‐exchange ability, LDHs can also be adopted to adsorb various anionic pollutants such as B(OH)^4−^, HAsO_4_^2−^, Cl^−^ in water treatment.^[^ ^249^, ^250^, ^251^ ^]^

Calcined layered double hydroxides (CLDHs), also known as mixed metal oxides (MMOs), are prepared by the calcination of the LDHs at intermediate temperature. With an increase of temperature, the interlayer water, anions, and hydroxyl groups of the host layer are gradually eliminated.^[^ ^57^ ^]^ These MMOs can regenerate the initial layered LDH structure by anion insertion, and this recovery process is the so‐called “memory effect”.^[^ ^252^, ^253^ ^]^ Therefore, calcined LDHs are of interest as Cl^−^ capture electrodes for water desalination. Hu et al.^[^ ^79^ ^]^ synthesized NiCoAl‐mixed metal oxides (NiCoAl‐MMOs) by calcination of NiCoAl‐LDHs at 500 °C as depicted in Figure 22b, and used the NiCoAl‐MMOs as the Cl^−^ capture electrode for the HCDI cell in combination with carbon fiber as the Na^+^ capture electrode. An ultrahigh salt removal capacity of 108.8 mg g^−1^ was realized by this system based on the concomitant reconstruction of the layered structure by intercalating anion. Ren et al.^[^ ^57^ ^]^ prepared MgAl‐O*_x_* nanosheets combined with graphene (MgAl‐O*_x_*/G) by calcination of MgAl‐LDHs/G at 400 °C, and used the MgAl‐O*_x_*/G nanohybrids as the Cl^−^ capture electrode for the HCDI cell. The calcined MgAl‐O*_x_* nanosheets retained the thin hexagonal structure of MgAl‐LDHs well, as shown in Figure 22c,d, and the corresponding HCDI cell exhibited a salt removal capacity of 13.6 mg g^−1^ and a salt removal rate of 1 mg g^−1^ min^−1^ as shown in Figure 22e.

It is noticeable, however, that reconstruction only occurs within a limited calcination temperature range. Since the interlayer water, anions and hydroxyl groups of the host layer are eliminated gradually as the temperature increases, the calcinated temperature should be high enough to eliminate most of the interlayer water and anions. Meanwhile, if the temperature is too high, some stable phases such as the M^II^M^III^_2_O_4_ or M^II^O phases will appear and the hydrotalcite‐like structure will not be reconstructed by memory effect.^[^ ^254^, ^255^ ^]^ So far, no study has investigated the relationship between calcination temperature and retention of the structure. In addition, the reconstruction mechanism and the charge transfer between ions the calcined LDHs layers have not been explained in detail. These critical issues need to be further investigated for utilization of these advanced electrode materials.

### 3.3 Emerging Materials for Both Na^+^ and Cl^−^ Capture

Inspired by the unique properties of graphene, many other 2D materials have also been widely studied and explored for their potential applications in various fields such as catalysis, solar cells, batteries and supercapacitors.^[^ ^244^, ^256^ ^]^ Recently, MXenes and transition metal dichalcogenides have emerged as promising electrode materials for both Na^+^ and Cl^−^ capture in CDI application because of their unique interlayer insertion properties. Redox‐active polymers also have shown their potential as CDI electrodes for both Na^+^ and Cl^−^ capture due to the tunability and multiformity of their redox active moieties.

#### 3.3.1 MXenes

MXenes are a class of 2D materials first reported in 2011.^[^ ^257^ ^]^ MXene with formula of M_n+1_X_n_T*_x_* (n = 1, 2, or 3; M stands for transition metal such as Mo, Ti or V, etc; X denotes C and/or N, and T represents surface terminal group such as ‐OH or ‐F) can be prepared through selective etching of the A atomic layers of raw MAX phase (M_n+1_AX_n_, A represents the element of IIIA or IVA group such as Si or Al).^[^ ^258^ ^]^ Apart from their highly hydrophilicity due to the surface terminal groups and metallical electronic conductivity,^[^ ^259^ ^]^ MXenes are also capable of highly reversible interlayer insertion/extraction of ions and show satisfactory pseudocapacitance in aqueous solution.^[^ ^260^, ^261^ ^]^ These combined properties make MXenes a promising host for desalination, which have facile transport channels for both ions and electrons to realize appreciable salt removal capacity.

**Figure 23:**
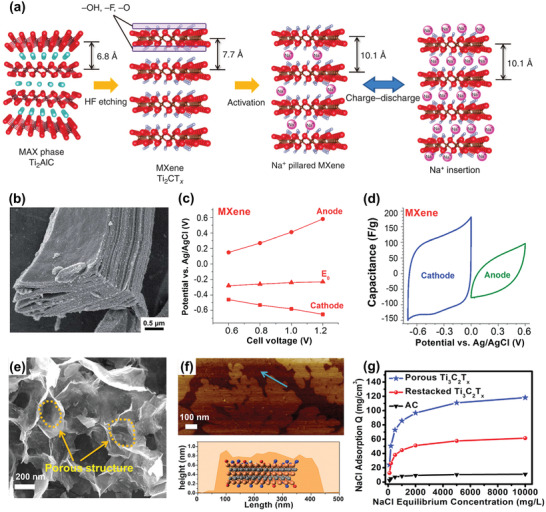
a) Schematic illustration of the Na^+^ insertion/extraction of Ti_2_CT*_x_*‐MXene. Reproduced with permission.^[^ ^134^ ^]^ Copyright 2015, Springer Nature. b) SEM image of Ti_3_C_2_‐MXene, c) electrode potential at different cell voltages, and d) cyclic voltammograms at 5 mV s^−1^ for anode and cathode of Ti_3_C_2_‐MXene in 1 m NaCl solution. Reproduced with permission.^[^ ^54^ ^]^ Copyright 2016, The Royal Society of Chemistry. e) FESEM image, f) (up) AFM image and f) (bottom) thickness profiles along the arrow of Ti_3_C_2_T*_x_*‐MXene, and g) salt removal capacity of porous Ti_3_C_2_T*_x_*, restacked Ti_3_C_2_T*_x_*, and AC electrodes in different equilibrium NaCl concentration. Reproduced with permission. Reproduced with permission.^[^ ^58^ ^]^ Copyright 2018, Elsevier.

The Na^+^ insertion/extraction of MXene was proved by Wang et al.^[^ ^134^ ^]^ They found that the interlayer distance of Ti_2_CT*_x_* broadens from 0.68 to 0.77 nm after etching the Al atomic layers and further expands to 1.01 nm after the first Na^+^ insertion, and that the Na^+^ insertion/extraction process occurs reversibly without obvious interlayer distance change, as displayed in **Figure** **23**a. In 2016, Srimuk et al.^[^ ^54^ ^]^ assembled a symmetric CDI using Ti_3_C_2_‐MXene with delaminated structure as both the cathode and anode for the first time as shown in Figure 23b, which exhibited a stable desalination capacity of 13 ± 2 mg g^−1^ over 30 cycles. However, the surface terminal groups of MXene, such as ‐OH, = O and ‐F, led to a negative charge of host layers.^[^ ^134^, ^262^ ^]^ As a result, the potential of the MXene electrode was shifted to negative values (E_0_) by 250 mV versus Ag/AgCl as compared in Figure 23c. Consequently, the electrode within positive polarization delivered a higher specific capacitance of 176 F g^−1^ at 0.1 A g^−1^ than that within negative polarization (84 F g^−1^) as shown in Figure 23d. The unbalance between two electrode capacities inevitably leads to dissatisfactory desalination performance in symmetric CDI based on two identical electrode materials.

To overcome this unbalance issue, Srimuk et al.^[^ ^96^ ^]^ further introduced a different MXene material, i.e., Mo_1.33_C, whose potential of zero charge is close to 0 V, into symmetric CDI. The positively and negatively polarized electrodes achieved similar specific capacitances of 150 and 155 F g^−1^ at 0.1 A g^−1^, and the removal capacities for Na^+^ and Cl^−^ were ≈0.2 mol g^−1^ in 5 × 10^−3^ m NaCl solution. As a result, the prepared CDI with two identical Mo_1.33_C‐CNT electrodes reached a desalination capacity of 15 mg g^−1^ with an excellent charge efficiency of 95% in 600 × 10^−3^ m NaCl solution.

Furthermore, considering that the interlayer stacking of MXenes severely decreases the exposure of accessible spaces and electrochemical active sites, the design of porous 3D materials is proposed as an effective approach to achieve a more effective and fast response to ion insertion/extraction.^[^ ^58^, ^263^, ^264^ ^]^ Correspondingly, as shown in Figure 23e, Bao et al.^[^ ^58^ ^]^ prepared aerogel‐like Ti_3_C_2_T*_x_* MXenes using a vacuum freeze‐drying method to prevent the restacking of MXene nanosheets. The porous Ti_3_C_2_T*_x_* MXenes possessed a high specific surface area of 293 m^2^ g^−1^, in which the Ti_3_C_2_T*_x_* layers were very thin (≈0.9 nm) as presented in Figure 23f. Such a 3D porous structure with abundant surface groups provides abundant diffusion channels and sufficient active sites for ion insertion. Hence, the CDI consisting of two symmetric Ti_3_C_2_T*_x_*‐MXene electrodes exhibited a drastically higher desalination capacity of 118 mg cm^−3^ (45 mg g^−1^) than that of the restacked Ti_3_C_2_T*_x_* and AC in 10 m NaCl solution as compared in Figure 23g. In addition to the design of porous 3D, there are also some other strategies to increase the interlayer stacking of MXenes. For example, Amiri et al.^[^ ^263^ ^]^ introduced heteroatom nitrogen into MXene structure and thus fabricated a porous nitrogen‐doped Ti_3_C_2_T*_x_* (N–Ti_3_C_2_T*_x_*) as CDI electrode, which exhibited a desalination capacity of 117±4.7 mg cm^−3^ (43.5±1.7 mg g^−1^) in 5000 mg/L NaCl solution. Moreover, Ma et al.^[^ ^264^ ^]^ used the method of LiF/HCl etching to increase the interlayer distance of Ti_3_C_2_T*_x_* to 1.28 nm, much higher than HF‐etched Ti_3_C_2_T*_x_* in other studies (≈0.8 nm).^[^ ^134^ ^]^ As a result, an ultrahigh salt removal capacity of 67.7 mg g^−1^ was realized in 10 × 10^−3^ m NaCl solution at 20 mA g^−1^.

#### 3.3.2 Transition Metal Dichalcogenides

Transition metal dichalcogenides (TMDs) are another class of layered 2D materials with a formula of MX_2_ (M stands for transition metal such as Mo, W, Ti, Ta, Zr, Nb, etc; X denotes chalcogen such as S, Se, or Te).^[^ ^265^, ^266^ ^]^ The laminar TMDs possess strong intralayer covalent bonds of M‐X and weak interlayer van der Waals forces, which could be exfoliated into single/few‐layer 2D TMD nanosheets.^[^ ^267^, ^268^ ^]^ As a result, TMDs can provide an attractive platform to electrochemically intercalate various ions with extraordinary efficiency,^[^ ^266^, ^269^ ^]^ and thus this type of materials have been widely used as electrodes in not only energy storage devices such as batteries,^[^ ^270^, ^271^ ^]^ and supercapacitor,^[^ ^272^ ^]^ but also CDI devices right now, which are highlighted in this paper.

**Figure 24:**
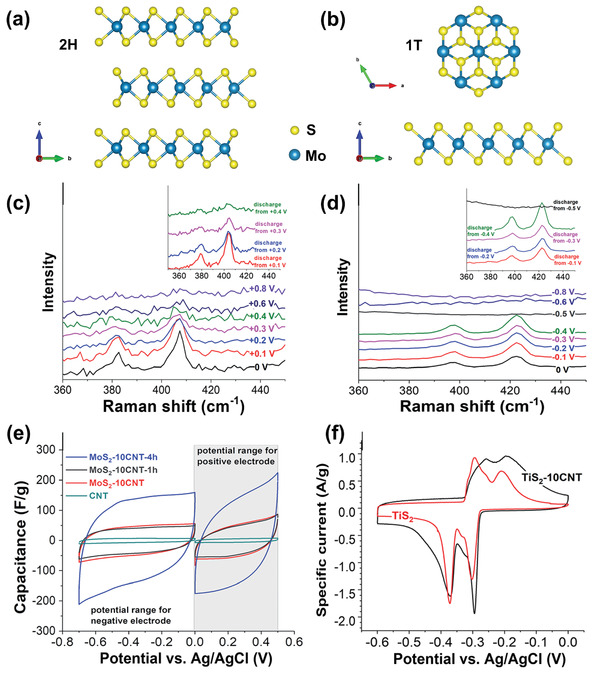
Crystal structures of MoS_2_ with a) 2H phase and b) 1T phase. Electrochemical in situ Raman patterns of MoS_2_/CNT in 1 m NaCl solution for c) positive polarization and d) negative polarization. e) CV curves of the as‐prepared positive and negative electrodes at 5 mV s^−1^. Reproduced with permission.^[^ ^55^ ^]^ Copyright 2017, The Royal Society of Chemistry. f) CV curves of the as‐prepared TiS_2_‐10CNT and TiS_2_ electrodes at 1 mV s^−1^. Reproduced with permission.^[^ ^122^ ^]^ Copyright 2017, American Chemical Society.

Xing et al.^[^ ^136^ ^]^ first investigated these 2D materials for CDI electrodes. They chemically exfoliated 2H phase MoS_2_ to prepare ultrathin MoS_2_ (ce‐MoS_2_) nanosheets with 1T phase. 2H phase and 1T phase have different combinations geometric models between Mo and S atoms as well as different stacking orders between layers as shown in **Figure** **24**a,b. The 2H phase possesses a trigonal prismatic coordination with hexagonal symmetry, and each layer has an atomic stacking sequence of S‐Mo‐S (ABA). This phase is the major form existing in nature due to its thermodynamic stability. As the 2H phase is exfoliated, the metallic 1T phase which has an atomic stacking sequence of S‐Mo‐S′ (ABC) in a tetragonal geometry and octahedral coordination appears gradually. The ce‐MoS_2_ with 1 T phase delivered a higher ion removal capacity (8.81 mg g^−1^) than the bulk MoS_2_ with 2H phase (≈2 mg g^−1^) in 400 × 10^−3^ m NaCl solution. This work proposed that the ion capture mechanism of ce‐MoS_2_ is achieved through electrosorption mechanism. However, this conclusion can't fully explain the fact that the high specific capacitance (110 F g^−1^) is difficult to be achieved just through electrosorption with an extremely low surface area (3.71 m^2^ g^−1^).

One more reasonable explanation is based on the Faradaic intercalation, which is proposed by Srimuk et al.,^[^ ^55^ ^]^ who introduced few‐layered MoS_2_/CNT hybrid electrodes into a symmetric CDI cell, and claimed that both cations and anions can intercalate between MoS_2_ layers by a Faradaic insertion rather than electrosorption as proposed above. Figure 24c,d shows the electrochemical in situ Raman patterns of MoS_2_/CNT in 1 m NaCl solution for positive polarization and negative polarization. The characteristic MoS_2_ peaks in the Raman patterns disappeared after applying a voltage over +0.4 V or below −0.4 V. After reversing the polarity of the electrode to 0 V, no peak recovery was observed. This indicated that the ion insertion caused an increase of interlayer distance, resulting in a lattice of MoS_2_ transformation from 2H to 1T. Regarding to the desalination performance, the CDI cell employing MoS_2_/CNT showed a salt removal capacity of 25 mg g^−1^ in 500 × 10^−3^ m NaCl solution, and achieved a drastically lower energy consumption of 24.6 kT per ion removed at 500 × 10^−3^ m solution compared to the CDI cell with AC (>20000 kT).

Similar to MXenes, with more or less negative charge of host layers,^[^ ^131^, ^266^ ^]^ the unbalance issue also exists in MoS_2_ materials, which are more favor for Na^+^ removal, and delivered a higher specific capacitance under positive polarization than that under negative polarization, as shown in Figure 24e. Therefore, we can develop some strategies to deal with this issue in further research, such as preparing 2D nanosheets with little charge to obtain similar cations and anions removal. Or, to put it another way, we can take their advantage of Na^+^ removal and use another electrode for Cl^−^ removal to achieve the balance. For example, Pattarachai et al.^[^ ^122^ ^]^ assembled a CDI cell of TiS_2_‐CNT//K20‐AC, in which the TiS_2_‐CNT composite is for Na^+^ intercalation, and the K20‐AC was used for the removal of anions through electrosorption. As shown in Figure 24f, the CV of TiS_2_ showed obvious redox peaks, illustrating its ion insertion mechanism. Consequently, the TiS_2_‐10CNT//K20 system revealed a salt removal capacity of 14 mg g^−1^ and a low energy of 29 kT per ion removal in a high salinity solution of 600 × 10^−3^ m.

Despite several recent investigations on the ion insertion/extraction mechanism of TMDs, further research is needed to make clear whether charge transfer occurs between ions and TMDs layer, thus further understanding the ion capture mechanism.

#### 3.3.3 Redox‐Active Polymer

Redox‐active polymers are interesting as ion removal electrode materials for CDI application due to their structural flexibility and diversity, surface functionalities and tenability, and avoidance of heavy metals.^[^ ^273^, ^274^, ^275^ ^]^ Based on the position of the redox active moiety in the structure, redox‐active polymers can be broadly classified into two classes: (i) redox‐active moiety‐suspended polymers, and (ii) redox‐active moiety‐embedded polymers. Due to their different chemical structure, they show different physicochemical properties and redox reactions.

**Figure 25:**
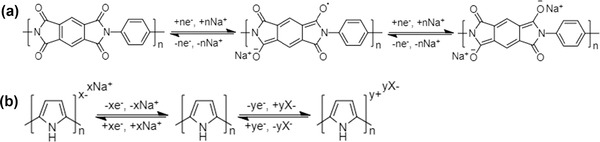
Schematic representation of a) the redox reaction of aromatic polyimide with Na^+^ and b) the p‐doping and n‐doping reactions of polypyrrole. Reproduced with permission.^[^ ^139^ ^]^ Copyright 2017, MDPI.

Redox‐active moiety‐suspended polymers generally possess redox‐active moieties on their nonconductive polymer backbones, including polymers with carbonyl groups such as polyimide,^[^ ^56^ ^]^ polyquinone^[^ ^137^ ^]^ and polymers with radical groups such as nitroxyl,^[^ ^276^ ^]^ and phenoxyl^[^ ^277^ ^]^ radical groups. A representative example of redox reaction of polymers with carbonyl groups with Na^+^ includes aromatic polyimide, which can be expressed as the equation shown in **Figure** **25**a.

**Figure 26:**
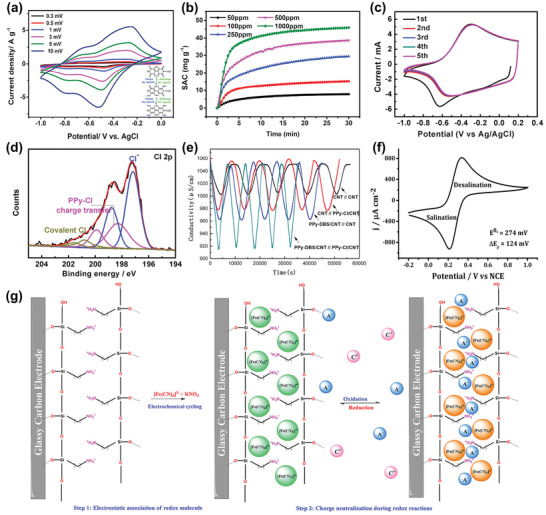
a) CV curves of PNDIE electrode at different scan rates in 1 m NaCl solution. Insert is the schematic of the redox reaction of PNDIE with Na^+^. b) CDI performances of PNDIE//AC HCDI in NaCl solutions with different initial concentrations. Reproduced with permission.^[^ ^56^ ^]^ Copyright 2019, Elsevier. c) CV curves of PPyCl@CNTs electrode at 10 mV s^−1^ in 1 m NaCl solution, d) XPS spectra of Cl 2p of the PPyCl@CNTs. Reproduced with permission.^[^ ^138^ ^]^ Copyright 2019, WILEY‐VCH. e) Comparison of CDI performance of CNT // CNT, CNT // PPy‐Cl/CNT, PPy‐DBS/CNT // CNT, and PPy‐DBS/CNT // PPy‐Cl/CNT CDI cells. Reproduced with permission.^[^ ^118^ ^]^ Copyright 2016, Elsevier. f) CV curve of the [Fe(CN)_6_]^4^‐PSQ electrode in real seawater and g) illustration of the preparation of [Fe(CN)_6_]^4^‐PSQ composite and its charge compensation process. Reproduced with permission.^[^ ^44^ ^]^ Copyright 2019, WILEY‐VCH.

Li et al.^[^ ^138^ ^]^ synthesized a redox‐active polyimide, poly[*N*,*N*′‐(ethane‐1,2‐diyl)‐1,4,5,8‐naphthalenetetracarboxiimide] (PNDIE). As shown in **Figure** **26**a, each CV displays two pair of redox peaks, corresponding to the two‐step redox reaction with Na^+^ along with the charge redistribution within the polyimide chain. The corresponding PNDIE//AC HCDI delivers a desalination capacity of 30.2 mg g^−1^ in 250 ppm NaCl solution, and achieves an increased capacity of 45.9 mg g^−1^ in 1000 ppm NaCl solution (Figure 26b).

Redox‐active moiety‐embedded polymers have redox‐active backbones consisting of active monomers. The most typical ones involve conjugated conductive polymers, which undergo redox reactions through doping/dedoping ions on the *π* ‐conjugated double bonds of polymer backbone.^[^ ^138^, ^278^ ^]^ They can either be p‐doped with counter‐anions, or n‐doped with counter‐cations to maintain electrical neutrality,^[^ ^139^, ^278^ ^]^ as shown in the representative equation (Figure 25b) using polypyrrole as an example.

In general, most of the typical conjugated conductive polymers are more inclined to be p‐doped with counter‐anions, termed as the p‐type polymers, such as polyaniline (PANi) and polypyrrole (PPy). For example, Kong et al.^[^ ^138^ ^]^ reported a p‐type polypyrrole doped with chloride ion (PPyCl) as the Cl^−^ capture anode for desalination, coupled with a Na_0.44_MnO_2_ as the Na^+^ capture cathode. As shown in CV curves (Figure 26c), the redox couple of the PPyCl anode corresponded to the release/capture of Cl^−^ from/into the PPyCl anode. The XPS spectra displayed in Figure 26d further demonstrated that the intermediate Cl 2p3/2 signal at 198.3 eV was related to the anionic chlorides, which are caused by the charge‐transfer interaction between Cl^−^ and the polypyrrole chain.

It is interesting to note that p‐type (oxidized) PPy possesses ion exchange ability, which depends on the dopant species.^[^ ^279^ ^]^ To be specific, PPy with small dopant anions, such as Cl^−^, NO_3_^−^, SO_4_^2−^ and ClO_4_^−^, generally displays anion exchange behavior because of the high mobility of these anions in the PPy chain.^[^ ^279^ ^]^ In contrast, cation exchange activity can occur mainly on PPy doped with large anions, such as dodecyl sulfonate (DS^−^), dodecyl benzene sulfonate (DBS^−^), polyvinylsulfonate (PVS^n−^), and polystyrene sulfonate (PSS^n−^), because of the immobility of these anions in the PPy matrix.^[^ ^279^ ^]^ For example, Wang et al.^[^ ^280^ ^]^ reported a PPy doped with DBS^−^/carbon nanotube (PPy‐DBS@CNT) as the Na^+^ capture cathode for CDI application, and the fabricated PPy‐DBS@CNT//CNT CDI cells showed a salt removal capacity of 43.99 mg g^−1^, which was much higher compared to the CNT//CNT CDI cell (≈11.00 mg g^−1^). Along these lines, the authors also doped Cl^−^ into PPy to fabricate a PPy‐Cl/CNT anode, which was combined with a PPy‐DBS/CNT cathode to construct a PPy‐DBS/CNT//PPy‐Cl/CNT CDI cells.^[^ ^118^ ^]^ As shown in Figure 26e, the CDI cell displayed improved SAC of 72.36 mg g^−1^, much higher than the other three assembled cells (PPy‐DBS/CNT//CNT: 48.96 mg g^−1^; CNT // PPy‐Cl/CNT: 40.80 mg g^−1^; CNT//CNT: 12.6 mg g^−1^).

The ion capture behavior of polymers can be modified by integrating redox‐active moieties on polymer backbones, where the moieties includes functional groups, electronic dopants and metallocenes.^[^ ^140^, ^281^, ^282^, ^283^ ^]^ For instance, by doping —SO_3_^−^ groups onto the PANI chain, the self‐doped PANI/functionalized carbon cloth (SPAN/FC) electrode^[^ ^140^ ^]^ mainly undergoes interactions with Na^+^ rather than p‐doping reactions with anions. Similar strategies were also conducted for self‐doped polypyrrole with ionizable sodium sulfonate^[^ ^282^ ^]^ and polyaniline with self‐doped benzenesulfonic anions.^[^ ^283^ ^]^ Another case was demonstrated by Silambarasan et al.,^[^ ^44^ ^]^ who introduced a redox‐active [Fe(CN)_6_]^4−^ immobilized polysilsesquioxane ([Fe(CN)_6_]^4^‐PSQ) composite film as Cl^−^ capture electrode for sea water desalination, represented in the following reaction of PSQ‐[Fe(II)(CN)_6_]^4−^(film) + Cl^−^ ↔ PSQ‐[Fe(III)(CN) _6_]^3−^ · Cl^−^ (film) (Figure 26f,g). As a result, the [Fe(CN)_6_]^4^‐PSQ based CDI cell, in combination with a NiHCF film as the Na^+^ capture material, achieved a superior desalination performance. About 164 mg L^−1^ of Cl^−^ was removed from real seawater per 7.6 × 10^−8^ mol cm^−2^ surface concentration of the [Fe(CN)_6_]^4^‐PSQ electrode with a high coulombic efficiency of 98%. An advanced electrochemical cell composed of two polymers was reported by Achilleos et al.^[^ ^141^ ^]^ to capture carboxylate salts. In this cell, the poly(vinylferrocene) (PVF)‐based anode presents affinity for carboxylates based on their hydrophobicity and basicity, and the poly(anthraquinone) (PAQ)‐based cathode captures the counterions of these carboxylates by undergoing corresponding electrochemical reductions. As a result, a remarkable electrochemical performance with a super‐high desalination capacity of 122–157 mg_anions_ g^−1^ was achieved, providing new possibilities for the development of polymer‐based electrochemical cells with high desalination performance.

For redox‐active polymer electrode materials, functional groups, electronic dopants and metallocenes can act as redox active moieties for interacting with ions in saline solution. However, this research remains in its infancy. Some issues, such as the low involvement of functional groups in redox reactions, unsatisfactory dopant level (0.3–0.5) of polymers,^[^ ^284^ ^]^ and poor intrinsic electronic conductivity needs to be solved. Therefore, more effort is required to optimize polymer materials as efficient and reversible ion capture electrodes for water desalination.

**Figure 27:**
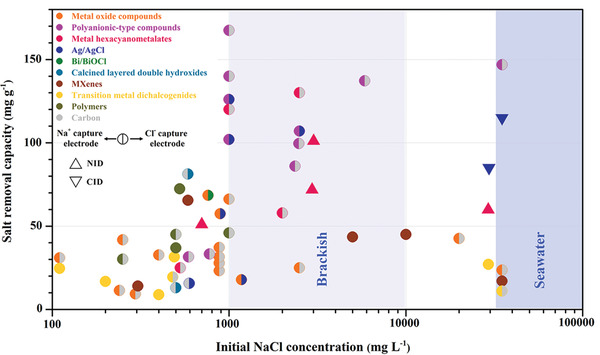
Comparison for salt removal capacity of representative Faradaic electrode‐based CDI cells in different concentration NaCl solution. Specifically, 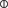 represents a full‐cell configuration, in which the left and right half respectively represents Na^+^ and Cl^−^ capture electrode material. Δ represents a sodium‐ion desalination (NID). ∇ represents a chloride‐ion desalination (CID).

So far, we have discussed various Na^+^ and Cl^−^ capture Faradaic electrode materials from microcosmic crystal structure and ion capture mechanism to macrocosmic electrochemical behaviors. For a better overview about current Faradaic electrode‐based CDI cells, a summary of representative CDI cells with various Na^+^ and Cl^−^ capture electrode materials is presented, as listed in **Table** **1**, which includes the cell configuration with various electrode materials and related performance evaluations. And especially, an intuitive comparison for salt removal capacity of these CDI cells in different concentration NaCl solution is plotted in **Figure** **27**. It can be seen that although most cells operate in concentrations below brackish water with salt removal capacities below 100 mg g^−1^, some cells already exhibit high capacities above 100 mg g^−1^ in high‐concentration brackish and seawater, showcasing their potential in practical water desalination. Specifically, among the Na^+^ capture electrodes, CDI cells based on Mn/Ti/V‐based metal oxide compounds generally exhibit salt removal capacity below 70 mg g^−1^ in relatively low concentrations of brackish water. NASICON‐type phosphates and metal hexacyanometalates electrodes stand out in brackish and seawater, in which metal hexacyanometalates provide salt removal capacity with 60–130 mg g^−1^ while NASICON‐type phosphates are higher with 100–150 mg g^−1^. Among the few Cl^−^ capture electrodes, Ag‐based electrodes have capacities of ≈100 mg g^−1^ at concentrations of brackish and above. Among the electrodes that capture both Na^+^ and Cl^−^, MXenes and transition metal dichalogenides operate at a wide range of salt concentration, albeit with low capacity (<50 mg g^−1^), while polymers are clustered in the below‐brackish concentration with low capacity below (<50 mg g^−1^). It should be noted that, this figure just present the salt removal capacity of different CDI cells based on materials sets as a visual graph, and we should caution against over interpreting the pros and cons of these cells and materials. Due to the complicated relationship between cell system design, operational conditions and desalination performance, and considering the incomplete data of other critical performance metrics as seen in Table 1, it is impossible to comprehensively and rationally compare the desalination performance of these cells, and it is hard to definitely determine the best Faradaic material. For the practical application, we should focus more on the comprehensive desalination performance rather than just the salt removal capacity. Furthermore, more investigation at high salinity such as seawater should be conducted.

**Table 1:** Desalination performances of representative Faradaic electrode‐based CDI cells in NaCl solution

Material types	Na^+^ capture electrode material	Cl^−^ capture electrode material	IEM	BM/SP	Flow Rate [mL min^−1^]	Applied voltage/Current density	Initial TDS [mg L^−1^]	SRC^a)^ [mg g^−1^]	SRR [mg g^−1^ s^−1^]	Charge efficiency	Energy consumption	Capacity retention [%] after *N* cycles	Ref.
Metal oxide compounds	*α*‐MnO_2_	AC	CEM + AEM	BM	20	1.2 V	880	22.1	0.111	–^b)^	–	≈100% after 20 cycles	^[^ ^109^ ^]^
	*α*‐MnO_2_/graphene	graphene	CEM + AEM	BM	10	1.2 V	100	29.5	0.01	–	–	–	^[^ ^285^ ^]^
	Mg‐buserite	carbon	CEM + AEM	BM	20	1.2 V	880	37.2	–	–	–	75% after 200 cycles	^[^ ^128^ ^]^
	Na‐birnessite	carbon	CEM + AEM	BM	20	1.2 V	880	31.5	–	–	–	66% after 200 cycles	^[^ ^128^ ^]^
	CNT/NaMnO_2_	AC	None	BM	–	1.2 V	500	32.7	0.095^c)^	90%^c)^	–	93% after100 cycles at 0.8 V	^[^ ^286^ ^]^
	HC@MnO_2_	PHC	None	BM	9	1.2 V	500	30.7	0.13^c)^	73.90%	–	90.1% after 50 cycles at 0.8 V	^[^ ^287^ ^]^
	2D *δ*‐MnO_2_	AC	None	BM	30	1.0 V	295	9.93	–	–	–	–	^[^ ^103^ ^]^
	3D *λ*‐MnO_2_	AC	None	BM	30	1.0 V	295	9.35	–	–	–	–	^[^ ^103^ ^]^
	MnO_2_	AC‐QPVP	None	SP	–	1.0 V	500	8.4	–	81%	–	90.5% after 350 cycles	^[^ ^76^ ^]^
	MnO_2_@ACC	Ag@ACC	None	SP	–	1.2 V	1175	17.8	0.027^c)^	83%	–	–	^[^ ^108^ ^]^
	Na_0.44_MnO_2_	AgCl	CEM + AEM	BM	100	100 mA g^−1^	890	57.4	0.022	>95%	–	52% after100 cycles	^[^ ^112^ ^]^
	Na_0.44_MnO_2_	BiOCl	CEM + AEM	BM	100	100 mA g^−1^	760	68.5	0.021	>95%	–	68.5% after 100 cycles	^[^ ^47^ ^]^
	Na_4_Mn_9_O_18_	PC	AEM	SP	10	1.2 V	585	31.2	0.065^c)^	–	–	–	^[^ ^46^ ^]^
	carbon@Na_4_Ti_9_O_20_	AC	CEM + AEM	BM	34	1.4 V	1000	66.14	–	–	–	–	^[^ ^107^ ^]^
	Graphene@Na_4_Ti_9_O_20_	AC	AEM	BM	34	1.4 V	250	41.8	–	≈100%	–	–	^[^ ^106^ ^]^
	Na_2_Ti_3_O_7_‐CNT@rGO	AC@rGO	CEM + AEM	BM	50	145 mA g^−1^	3000	129	0.04	–	0.42 Wh g^−1^	–	^[^ ^102^ ^]^
	Li_4_Ti_5_O_12_@carbon	carbon cloth	CEM + AEM	BM	50	0.8 mA	2500	25	0.013^c)^	83%	9.92 × 10^−20^ J per ion	≈100% after 30 cycles	^[^ ^189^ ^]^
	MWCNT–hV_2_O_5_	PC	CEM + AEM	SP	5	0.8 V	35 000	23.6	–	87%	17.9 kT	85% after 15 cycles^c)^	^[^ ^123^ ^]^
	Na_1.1_V_3_O_7.9_@rGO	Ag@rGO	CEM + AEM	BM	40	1.4 V	250	40	0.0006^c)^	–	–	≈100% after 10 cycles	^[^ ^192^ ^]^
	RuO_2_‐AC	AC	None	BM	5	1.2 V	295	11.26	0.0031^c)^	60%	–	86% after 10 cycles	^[^ ^203^ ^]^
	rGO/Co_3_O_4_	AC	None	BM	10	1.6 V	250	18.63	0.048	–	–	71% after 100 cycles at 1.0 V	^[^ ^204^ ^]^
Polyanion‐type compounds	NaTi_2_(PO_4_)_3_/rGO	AC	CEM + AEM	BM	400	100 mA g^−1^	1000	140	0.044	–	–	86% after 100 cycles	^[^ ^116^ ^]^
	NaTi_2_(PO_4_)_3_/rGO	AgNPs/rGO	CEM + AEM	BM	–	100 mA g^−1^	2500	105	–	–	0.127 Wh g^−1^	95% after 50 cycles	^[^ ^95^ ^]^
	NaTi_2_(PO_4_)_3_/carbon	AC	None	BM	100	1.8 V	1000	167.4	0.18^c)^	85%^c)^	–	90% after 30 cycles	^[^ ^77^ ^]^
	NaTi_2_(PO_4_)_3_/rGO	AC	None	BM	20	1.4 V	786	33.25	0.3	–	–	≈100% after 10 cycles	^[^ ^288^ ^]^
	NaTi_2_(PO_4_)_3_@C	AC	None	BM	10	2.0 V	35 000	146.8	0.041	–	–	–	^[^ ^289^ ^]^
	Na_3_V_2_(PO_4_)_3_/GA	AgCl/GA	CEM + AEM	BM	100	100 mA g^−1^	1000	124	0.035	–	–	87% after 50 cycles	^[^ ^94^ ^]^
	Na_3_V_2_(PO_4_)_3_@C	AC	AEM	BM	15	1.0 V	5850	137.2	0.076	–	2.157 kg kWh^−1^	–	^[^ ^41^ ^]^
	Na_3_V_2_(PO_4_)_3_@C	AgCl	CEM + AEM	BM	100	100 mA g^−1^	1000	102	0.04	–	–	96% after 50 cycles	^[^ ^115^ ^]^
	Na_2_FeP_2_O_7_	AC	AEM	SP	2	1.2 V	585	30.2	0.081	–	–	–	^[^ ^111^ ^]^
	FePO_4_@rGO	AC	CEM + AEM	BM	300	100 mA g^−1^	2500	100	–	–	3.57 × 10^−4^ kWh g^−1^	≈100% after 50 cycles	^[^ ^101^ ^]^
	FePO4@rGO	rGO	AEM	BM	200	1.8 V	2360	85.94	0.24	91.40%	9.0 × 10^−4^ kWh g^−1^	≈100% after 10 cycles	^[^ ^218^ ^]^
Metal hexacyanometalates	FeFe(CN)_6_@NPG	AC	CEM + AEM	BM	650	125 mA g^−1^	1000	120	–	–	6.76 kT	≈100% after 600 cycles at 625 mA g^−1^	^[^ ^124^ ^]^
	FeFe(CN)_6_/rGA	rGA	CEM + AEM	BM	50	100 mA g^−1^	2500	130	0.016^c)^	–	0.37 Wh g^−1^	≈100% after 100 cycles	^[^ ^229^ ^]^
	K_0.03_Cu[Fe(CN)_6_]_0.65_·0.43H_2_O	PC	None	SP	360	1.2 V	500	23.2	0.24	75.85%	–	–	^[^ ^40^ ^]^
	NiHCF/rGO	AC	None	BM	–	0.6 V	500	22.8	0.125	–	–	78% for 100 cycles	^[^ ^290^ ^]^
	NiHCF	None	AEM	SP^d)^	–	0.4 V	700	51	–	–	–	–	^[^ ^48^ ^]^
	NaNiHCF / NaFeHCF	None	AEM	SP^d)^	–	0.5 mA cm^−2^	29 250	59.9	–	–	0.34 Wh L^−1^	–	^[^ ^50^ ^]^
	CuHCF	None	CEM + AEM	SP^d)^	0.5	2.8 A m^−2^	2950	100	–	–	0.02 kW h m^−3^ at 1475 mg L^−1^ TDS	–	^[^ ^52^ ^]^
	CuHCF@NiHCF	None	AEM	SP^d)^	–	0.5 mA cm^−2^	2950	71.8	–	–	0.0376 mWh g^−1^	92% for 50 cycles	^[^ ^53^ ^]^
Ag/AgCl	carbon	Ag@carbon	CEM	BM	2	0.7 V	585	15.6	0.078	92.20%	73.3 kJ mole^−1^	–	^[^ ^78^ ^]^
	None	Ag/AgCl	CEM	SP^d)^	5	0.1 V	35 000	115	–	98%	2.5 kT	–	^[^ ^83^ ^]^
	None	Ag/AgCl	CEM	SP^d)^	–	1 mA cm^−2^	29 500	85	0.008	–	10 kJ mole^−1^	–	^[^ ^82^ ^]^
Calcined layered double hydroxides	Carbon fiber	NiCoAl‐MMO	None	BM	–	1.0 V	176	108.8	–	–	–	–	^[^ ^79^ ^]^
	AC‐HNO_3_	MgAl‐O*_x_*/G	None	SP	10	1.0 V	500	13.6	0.017^c)^	–	–	≈100% after 12 cycles	^[^ ^57^ ^]^
	graphite	NiAl‐MMO	None	BM	–	1.2 V	585	81.2	0.015	–	–	≈100% after 15 cycles	^[^ ^80^ ^]^
MXene	Ti_3_C_2_	Ti_3_C_2_	None	SP	22	1.2 V	295	13	0.017	–	–	≈100% after 30 cycles	^[^ ^54^ ^]^
	Porous Ti_3_C_2_T*_x_*	Porous Ti_3_C_2_T*_x_*	None	BM	40	1.2 V	10 000	45	–	–	–	≈100% after 60 cycles at 500 mg L^−1^ TDS	^[^ ^58^ ^]^
	LiF/HCl‐etched Ti_3_C_2_T*_x_*	LiF/HCl‐etched Ti_3_C_2_T*_x_*	None	BM	20	20 mA g^−1^	585	67.7	0.013	–	0.24 kWh kg^−1^	–	^[^ ^264^ ^]^
	Porous nitrogen‐doped Ti_3_C_2_T*_x_*	Porous nitrogen‐doped Ti_3_C_2_T*_x_*	None	BM	30	1.2 V	5000	43.5	0.003^c)^	–	–	≈100% after 25 cycles	^[^ ^263^ ^]^
	Mo_1.33_C‐CNT	Mo_1.33_C‐CNT	None	SP	15	0.8 V	35 000	15	0.006	95%	17.0 kT	≈100% after 40 cycles	^[^ ^96^ ^]^
Transition metal dichalcogenides	ce‐MoS_2_	ce‐MoS_2_	None	BM	–	1.2 V	400	8.81	0.002^c)^	–	–	–	^[^ ^136^ ^]^
	MoS_2_/CNT	MoS_2_/CNT	None	SP	22	0.8 V	29 500	25	0.007	95%	24 kT	≈100% after 25 cycles	^[^ ^55^ ^]^
	Defect‐rich MoS_2_	defect‐rich MoS_2_	None	BM	25	0.8 V	100	24.6	–	–	–	≈100% after 20 cycles	^[^ ^291^ ^]^
	MoS_2_/rGO	MoS_2_/rGO	None	BM	18	1.0 V	200	16.82	–	–	–	–	^[^ ^292^ ^]^
	MoS_2_−graphene	AC	None	BM	60	1.2 V	500	19.4	–	–	–	–	^[^ ^293^ ^]^
	TiS_2_‐CNT	K20	None	SP	22	100 mA g^−1^	35 000	14	–	>85%	29 kT	≈100% after 70 cycles	^[^ ^122^ ^]^
	SnS_2_@GP	SnS_2_@GP	None	BM	40	1.2 V	500	30.32	0.013^c)^	–	–	–	^[^ ^294^ ^]^
Polymers	PPy‐DBS/CNT	CNT	None	BM	–	1.4 V	500	44	0.044^c)^	–	–	–	^[^ ^280^ ^]^
	CNT/PPy‐DBS	CNT/PPy‐Cl	None	BM	25	1.2 V	500	36.1	0.024^c)^	–	–	53% after 100 cycles	^[^ ^295^ ^]^
	PPy‐DBS/CNT	PPy‐Cl/CNT	None	BM	20	1.2 V	525	72.36	0.048^c)^	–	–	–	^[^ ^118^ ^]^
	PNDIE	AC	AEM	BM	–	1.8 V	250	30.2	0.038^c)^	73%	–	57% after 100 cycles	^[^ ^56^ ^]^

^a)^ Note: IEM, ion‐exchange membrane; BM, batch mode; SP, single pass; TDS, total dissolved salt; SRC, salt removal capacity; SRR, salt removal rate; HC, hollow carbon; PHC, positive charged hollow carbon; QPVP, quaternized poly(4‐vinylpyridine); ACC, activated carbon cloth; rGO, reduced graphene oxide; MWCNT, multiwalled carbon nanotube; PC, porous carbon; AgNPs, Ag nanoparticles; GA, reduced graphene oxide aerogel; NPG, reduced graphene oxide with nanopores; rGA, reduced graphene oxide aerogel; ce‐MoS_2_, chemically exfoliated MoS_2_; GP, graphite paper; PNDIE, poly[*N*,*N*′‐(ethane‐1,2‐diyl)‐1,4,5,8‐naphthalenetetracarboxiimide];

^b)^ Some data are not available;

^c)^ Calculated value;

^d)^ Double compartment.

## 4 Tailored Applications

In addition to water desalination, CDI systems based on Faradaic electrodes can also be used towards some tailored special applications, especially for the selective ion removal from a scientific perspective and contaminant removal from a practical perspective. According to the purposes of removal, selective ion removal can be divided into cations or anions removal, and specific ions removal. A particular focus is on the effect of the characteristics of electrode materials, properties of ions, and the interaction between electrodes and ions on the ion selectivity of Faradaic electrode material. While contaminant removal includes water softening, heavy metal removal, and biological nutrient removal.

### 4.1 Selective Ion Removal

#### 4.1.1 Selective Cation/Anion Removal

Generally, Faradaic electrode materials can be properly selected to remove either cations or anions. As discussed in Section 3, most of the electrode materials are selective toward either cation or anion removal. However, some electrodes can remove both cations and anions, such as 2D layered MXenes and transition metal dichalcogenides.^[^ ^54^, ^55^, ^96^ ^]^ These 2D electrode materials possess intrinsic negative charges (Section 3.3.1 and 3.3.2), leading to more favorable removal of cations than anions. Consequently, these 2D electrode materials are mostly used to remove a wide variety of cations.^[^ ^296^ ^]^

**Figure 28:**
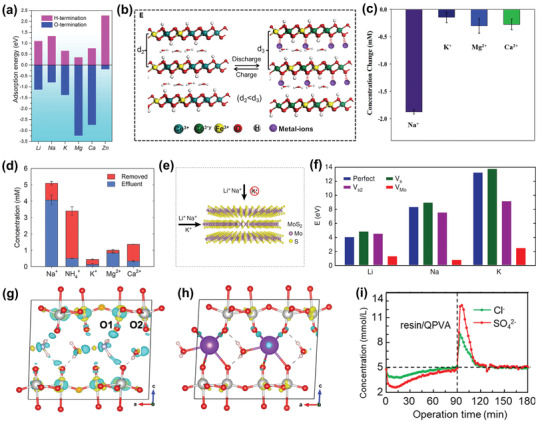
a) DFT calculations for adsorption energies of metal atom with OH‐terminated and O‐terminated LDH and b) schematic illustration for the metal ions insertion/extraction with ECA‐CoFe‐LDH. Reproduced with permission.^[^ ^298^ ^]^ Copyright 2018, Elsevier. c) Concentration changes for the selective separation of Na^+^ in the presence of other cations (K^+^, Mg^2+^, and Ca^2+^) using the Na_0.44−_ *_x_*MnO_2_//Ag system after the capture step in30 × 10^−3^ m NaCl, KCl, MgCl_2_, and CaCl_2_ solutions. Reproduced with permission.^[^ ^62^ ^]^ Copyright 2017, Elsevier. d) Concentration of removed and effluent cations measured by CuHCF//CuHCF CDI cell applied with 0.2 V for 200 s. Reproduced with permission.^[^ ^61^ ^]^ Copyright 2018, American Chemical Society. e) Schematic illustration for the insertion of alkali metal ions (Li^+^, Na^+^, and K^+^) into MoS_2_ through the top or edge channels and f) DFT calculations for the energy barriers for the insertion of alkali metal ions (Li^+^, Na^+^, and K^+^) through perfect MoS_2_, and the MoS_2_ with V_S_, V_S2_, and V_Mo_. Reproduced with permission.^[^ ^299^ ^]^ Copyright 2018, Springer Nature. g,h) Comparison of the charge movements for the insertion of NH_4_^+^ (tetrahedral shape) and K^+^ (spherical shape) in bilayered V_2_O_5_. Reproduced with permission.^[^ ^300^ ^]^ Copyright 2019, Elsevier. i) Concentration change of Cl^−^ and SO_4_^2−^ by resin/QPVA electrode during an adsorption and desorption cycle. Reproduced with permission.^[^ ^60^ ^]^ Copyright 2018, American Chemical Society.

Although the property and mechanism of Faradaic electrode materials determine their permselectivity toward either cations or anions, this can be tailored by modifying the electrochemical characteristic part of the Faradaic electrode materials. For instance, polymers with different characteristic redox active moieties can show preference for cations or anions by grafting an oppositely charged group.^[^ ^44^, ^140^, ^282^, ^297^ ^]^ In another example, LDHs normally prefer anion insertion due to their positively charged laminates. Li et al.^[^ ^298^ ^]^ creatively introduced hydrogen vacancies into LDHs via an electrochemical activation (ECA) strategy, which induces LDHs (repulsive to cations) to undergo a phase transition to hydrogen‐vacancy‐enriched LDHs (attractive to cations). The H‐vacancy‐induced active O termination shows negative adsorption energies toward wide range of metal atoms, as shown in **Figure** **28**a. Hence, the electrode based on hydrogen‐vacancy‐enriched LDHs can be used for the insertion of metal cations as shown in Figure 28b. Therefore, inspired by the above discussions, the enhancement of cation or anion removal through material modification is worth studying, especially for anions removal.

#### 4.1.2 Specific Ion Removal

Recently, investigations on specific ions removal have been developed, especially among ions with the same charge. Two situations for selective ion removal are introduced as follows.

1) Specific cation removal among other cations

As for the specific removal of a particular cations among other cations in solution such as Na^+^, K^+^, Mg^2+^, Ca^2+^, NH_4_^+^, etc., sodium manganese oxides,^[^ ^39^, ^62^ ^]^ NASICON‐type phosphates,^[^ ^301^ ^]^ and metal hexacyanometalates^[^ ^40^, ^51^, ^61^ ^]^ have been investigated.

**Figure 29:**
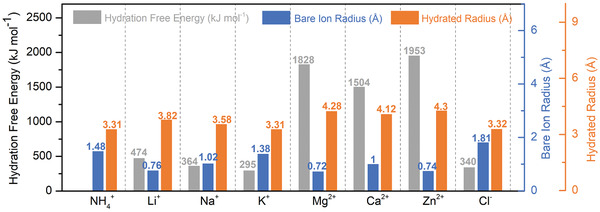
Comparison of three parameters between some typical ions, in which the data of hydration free energy is from ref. ^[^ ^303^ ^]^.

NMO and NTP both show affinity for Na^+^ over K^+^ and some divalent ions (Ca^2+^ and Mg^2+^).^[^ ^39^, ^62^, ^301^ ^]^ For example, Kim et al.^[^ ^62^ ^]^ determined that the NMO electrode is 13 and 6–8 times more selective toward Na^+^ compared to K^+^ and Mg^2+^/Ca^2+^ respectively in a mixed electrolyte containing equal concentrations of each ions (Figure 28c). NTP has been also evaluated,^[^ ^301^ ^]^ where its affinity toward Na^+^ removal is nearly an order of magnitude over K^+^, Ca^2+^, and Al^3+^. Depending on the lattice geometries of NMO and NTP, the favorability of ion insertion into the crystal lattice may be associated with losing their hydration shells. Therefore, the higher removal of Na^+^ over K^+^ can be explained by the smaller ionic radius of Na^+^ (0.102 nm) than K^+^ (0.138 nm) as compared in **Figure** **29**. In addition to the ionic radius, the ability of hydrated ions to hold on to their hydration shells is also very critical.^[^ ^302^ ^]^ Multivalent ions (i.e., Mg^2+^ and Ca^2+^) with relatively smaller crystal radii always possess higher hydration numbers and larger hydrated radii. Also, these ions retain their hydration shells more strongly. On the contrary, the ions with larger crystal radii (i.e., K^+^ and Na^+^) possess weaker hydration shells, and thus they can detach from their hydration layer easily. Therefore, the reason of higher selectivity for Na^+^ than for Mg^2+^ and Ca^2+^ can be explained by the lower hydration free energy of Na^+^ (364 kJ mol^−1^) than Mg^2+^ (1828 kJ mol^−1^) and Ca^2+^ (1504 kJ mol^−1^).

On the other hand, metal hexacyanometalates present a reverse trend with high K^+^ selectivity over Na^+^. Porada and Shrivastava^[^ ^51^ ^]^ found that NiHCF demonstrates threefold selectivity for K^+^ over Na^+^ under identical concentrations. A HCDI cell based on a K_0.03_Cu[Fe(CN)_6_] _0.65_·0.43H_2_O electrode also present a dramatically higher K^+^ removal compared to other cations (Na^+^, Mg^2+^, and Ca^2+^).^[^ ^40^ ^]^ Some studies have reported that NiHCF has an unique ion‐exchange property that, is quite selective among various monovalent ions (Cs^+^ > Rb^+^ > K^+^ > Na^+^ > Li^+^).^[^ ^304^, ^305^, ^306^ ^]^ This may provide a basis for the above experimental results. Metal hexacyanometalates also show preference for NH_4_^+^. A NID cell assembled with two CuHCF electrodes exhibited 9 times higher selectivity towards NH_4_^+^ over Na^+^ when tested with equimolar concentrations (10 × 10^−3^ m).^[^ ^61^ ^]^ When tested with real domestic wastewater, the CuHCF electrodes removed 85% of NH_4_^+^ with a selectivity over Na^+^ of >4 even in the presence of other competing cations (Figure 28d). A closer examination revealed that the K^+^/NH_4_^+^ selectivity of metal hexacyanometalates may also be correlated with the ion solvation conditions.^[^ ^307^, ^308^, ^309^, ^310^ ^]^ Na^+^ with a small bare ionic radius is likely to be co‐inserted with water molecules into the A sites of metal hexacyanometalates. On the contrary, K^+^ and NH_4_^+^ are larger and not only require dehydration prior to insertion, but also extrude some lattice water molecules from the host structure. Therefore, K^+^ and NH_4_^+^ insertion exhibit higher diffusion kinetics than Na^+^.

In the case of the 2D layered MXenes and transition metal dichalcogenides, a wide range of alkali metal ions can be inserted into the interlayer distance.^[^ ^296^, ^311^ ^]^ It is noteworthy that, material modification can generate electrodes with ion selectivity. For example, as reported by Zhang et al.,^[^ ^299^ ^]^ by sealing the edges of MoS_2_ flakes with natural defects, the MoS_2_ shows insertion preference for alkali metal ions with small ionic radius (e.g., Li^+^ and Na^+^) through the top surface, and rejects the ions with a large ionic radius (e.g., K^+^) (Figure 28e). DFT calculations (Figure 28f) indicate that the insertion is enabled by the existence of intrinsic defects in exfoliated MoS_2_ flakes, and the energy barrier for the insertion of K^+^ through these intrinsic defects is much higher than for Li^+^ or Na^+^, resulting in the ion selectivity of sealed‐edge MoS_2_ material. The selective insertion through the top surface was also applied to other similar 2D layered material such as MoSe_2_.^[^ ^299^ ^]^

In addition to the ionic size, charge number, and hydration free energy, the shape of cations also affects the selective insertion processes for different topotactic insertion chemistries.^[^ ^300^ ^]^ Recently, Dong et al.^[^ ^300^ ^]^ investigated the topotactic insertion of NH_4_^+^ and K^+^ in bilayered V_2_O_5_ through comprehensive characterizations and DFT calculations as shown in Figure 28g,h. Although NH_4_^+^ and K^+^ ions have equivalent hydrated radii and similar magnitudes of interaction with V_2_O_5_ (≈−250 eV, DFT computation), the spherical K^+^ does not have any preferred orientations, while NH_4_^+^ possesses a tetrahedral‐shaped multipole. During the migration inside the bilayered V_2_O_5_, NH_4_^+^ can twist and rotate to ensure strong directional hydrogen bonds with neighboring oxygen atoms (oxygen in either crystal water or V_2_O_5_), resulting in ultra‐fast NH_4_^+^ insertion.

2) Specific anion removal among other anions

As for the removal of a specific anion from a mixture of anions, LDHs^[^ ^251^, ^312^ ^]^ and polymers have been investigated. As mentioned in Section 3.2.3, LDHs possess high anion‐exchange capacities. In general, the ion selectivity of LDHs can be improved with higher electric charge and smaller size of the anion, in the sequence of CO_3_^2−^ >SO_4_^2−^ >OH^−^ >F^−^ > Cl^−^ > Br^−^ > NO_3_^−^ > I^−^.^[^ ^313^, ^314^ ^]^ For example, Cl^−^ can be removed from aqueous solution by ZnAl‐NO_3_ LDHs due to the fact that the Cl^−^ can be exchanged with NO_3_^−^.^[^ ^251^ ^]^

Polymers normally possess electrochemically induced anion‐exchange capacities. For example, a PPy film doped with Cl^−^ on CNTs was used for removing ClO_4_^−^ ions from wastewater, which is attributed to the comparative selectivity of different anions from PPy film: ClO_4_^−^ > Br^−^ > Cl^−^ > NO_3_^−^.^[^ ^315^ ^]^ A resin/quaternary amine functionalized poly(vinyl alcohol) (resin/QPVA) showed a selective CDI process for removal of SO_4_^2−^.^[^ ^60^ ^]^ Due to the selectivity towards SO_4_^2−^, the result presented higher removal of SO_4_^2−^ even in a mixture with Cl^−^: SO_4_^2−^ ratios up to 20:1 as shown in Figure 28i. Notably, the anion exchange selectivity of polymers is strongly affected by the incorporated counter‐ion. For a polypyrrole with Cl^−^, the anion selectivity is Br^−^ > SCN^−^ > SO_4_^2−^ > I^−^ > CrO_4_^2−^, and with ClO_4_^−^, the selectivity is SCN^−^ > Br^−^ > I^−^ > SO_4_^2−^ > CrO_4_^2−^.^[^ ^316^ ^]^

Above all, the ion selectivity of Faradaic electrode materials is strongly influenced by properties of material, such as the structural channels (tunnel pathway, interlayer distance, special lattice vacancies in subcells), intrinsic defects, functional terminals, and external material modification. Moreover, the intrinsic properties of ions also play important roles in ion selectivity of Faradaic electrodes, including the bare ion radii/hydrated radii, ionic charge numbers (monovalent, divalent, or trivalent ions), hydration free energies, and even the ionic topological shapes. In addition, the interaction between electrode material and ions is also significant, which has been investigated progressively and will further stimulate some new developments for ion selective removal.

### 4.2 Contaminant Removal

#### 4.2.1 Water Softening

Hard water contains high concentrations of minerals, such as Ca^2+^ or Mg^2+^ ions, resulting in scaling problems in pipelines of boilers and heat exchangers. Therefore, the water softening, i.e., the removal of Ca^2+^ or Mg^2+^, and certain other metal cations in hard water, is necessary and need to be urgently resolved. In this regard, CDI has been recently considered for water softening applications,^[^ ^317^ ^]^ in which the feasibility can be facilitated through the strong interactions between the Faradaic electrodes of CDI and the metals ions in hard water. A proof of concept was proposed by Byles et al.,^[^ ^109^ ^]^ who applied a series of tunnel structured manganese oxide electrodes to remove Mg^2+^, of which the 2 × n‐MnO_2_ exhibited the highest removal capacity of 454 µmol g^−1^ (i.e., 43.1 mg g^−1^) in 15 × 10^−3^ m MgCl_2_ solution. Additionally, they also used a Na‐birnessite electrode^[^ ^128^ ^]^ to remove Mg^2+^ with a removal capacity of 527 µmol g^−1^ (50.2 mg g^−1^) in the same solution. Other than this, Jung et al.^[^ ^318^ ^]^ prepared a zwitterionic polymer@AC composite for Ca^2+^ and Mg^2+^ removal. Due to the binding affinities between the zwitterionic polymer and alkali‐metal ions as calculated by DFT, the CDI cell with this electrode obtained a higher ion removal capacity of 30–35 mg g^−1^ compared to pure AC. However, with regard to wastewater containing other competing ions such as K^+^, Na^+^, NH_4_^+^, the removal of Ca^2+^ or Mg^2+^ is challenging, especially for CDI systems with metal hexacyanometalate electrodes.^[^ ^40^, ^61^ ^]^ Therefore, it is necessary to further develop selective electrode materials for water softening to improve the removal selectivity and capacity.

#### 4.2.2 Lithium Recovery

With the increasing use of lithium‐ion batteries, it has become urgent to develop effective methods for lithium recovery.^[^ ^319^, ^320^ ^]^ However, the most commonly used lithium‐extraction method, the lime‐soda evaporation process, is time‐consuming and strongly depends on weather conditions.^[^ ^321^ ^]^ Recently, electrochemical lithium recovery has been introduced as a possible alternative to extract lithium from Li^+^‐containing solutions like geothermal water or industrial wastewater due to its high efficiency, selectivity, and low energy consumption.^[^ ^319^, ^322^, ^323^ ^]^ This method generally uses lithium‐selective materials to recover lithium, such as LiFePO_4_/FePO_4_,^[^ ^324^, ^325^ ^]^ *λ*‐MnO_2_,^[^ ^326^, ^327^ ^]^ and lithium manganese oxides.^[^ ^328^, ^329^ ^]^ For example, Pasta et al.^[^ ^325^ ^]^ reported a lithium recovery system consisting of LiFePO_4_ as a Li^+^ capture electrode and Ag as a Cl^−^ capture electrode, which efficiently recovered lithium as LiCl from a sodium‐rich solution (mole ratio: Na/Li = 100/1). In order to reduce the cost, Trόcoli et al.^[^ ^324^ ^]^ modified the lithium recovery system by substituting the Cl^−^ capture electrode (Ag) with a low‐cost nickel hexacyanoferrate (KNiFe(CN)_6_) as Li^+^ exclusion electrode. Since this nickel hexacyanoferrate has a higher affinity toward cations of Na^+^ or K^+^ than for Li^+^, the system realized the use of seawater as a recovery solution and reduced the consumption of fresh water. Lee et al.^[^ ^327^ ^]^ developed a *λ*‐MnO_2_//Ag rechargeable cell and achieved a high lithium recovery from a stream that contains various cations with low energy consumption of 1.0 W h mole^−1^_Li_. A HCDI cell composed of a spinel lithium‐manganese‐titanium oxide and activated carbon has also been used to selectively extract Li^+^ from solutions.^[^ ^329^ ^]^ The process was characterized by an ultrahigh lithium salt removal capacity of 800 mg g^−1^ with a total energy consumption on the level of 0.183 W h per 1 g salt. Research into novel lithium‐selective electrodes materials as well as the optimization of system configuration is required for the improvement of lithium recovery performance and the reduction of energy consumption and cost.

#### 4.2.3 Heavy Metal Removal

Heavy metal pollution is often caused by metals such as lead, cadmium, copper, arsenic, and chromium. This has become a major health and environmental hazard across the world.^[^ ^330^ ^]^ After ingestion, toxic heavy metals may lead to serious diseases, such as itai‐itai disease, minamata disease, or even cancer.^[^ ^330^ ^]^ Correspondingly, CDI can be investigated and applied for the removal of these heavy metal ions, and some Faradaic electrode materials that have been used in this regard include manganese oxides,^[^ ^331^, ^332^, ^333^ ^]^ metal hexacyanometalates,^[^ ^334^ ^]^ and polymers.^[^ ^142^, ^335^, ^336^ ^]^ For example, Li et al.^[^ ^331^ ^]^ prepared a nanoneedle structured *α*‐MnO_2_/carbon fiber paper (*α*‐MnO_2_/CFP) hybrid electrode for removal of Ni^2+^ from industrial waste streams. Thanks to the efficient charge transfer via insertion reactions between Ni^2+^ ions and MnO_2_ hosts, the *α*‐MnO_2_/CFP electrode achieved higher removal capacity (16.4 mg Ni^2+^ g^−1^) compared to pure CFP electrode (0.034 mg Ni^2+^ g^−1^) and AC (2.5 mg Ni^2+^ g^−1^). Liu et al.^[^ ^333^ ^]^ studied tunnel‐structured manganese oxides for Cd^2+^ removal, in which the electrodes based on cryptomelane phase manganese oxide delivered an ultrahigh removal capacity of 192.0 mg g^−1^. They also investigated a 2D birnessite‐type manganese oxide/carbon nanotube composite electrode for Zn^2+^ and Ni^2+^ removal,^[^ ^332^ ^]^ and the removal capacities for Zn^2+^ and Ni^2+^ respectively reach up to 155.6 and 158.4 mg g^−1^.

**Figure 30:**
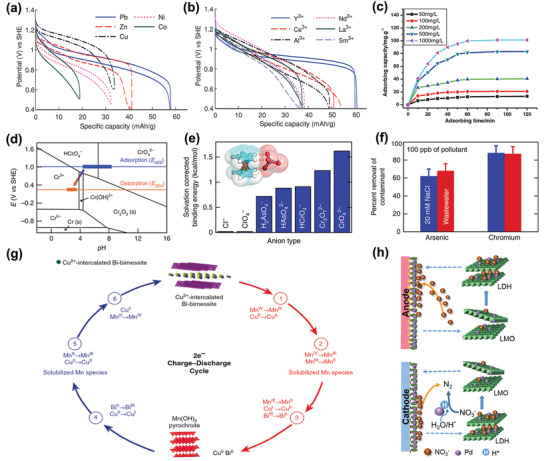
Galvanostatic cycling of a) divalent and b) trivalent ions in CuHCF at 5 C exhibits low voltage hysteresis and retention of specific capacity even at a high charge/discharge rate. Reproduced with permission.^[^ ^334^ ^]^ Copyright 2015, WILEY‐VCH. c) Salt removal capacities of polypyrrole–DBS/chitosan composite electrode over time in CuCl_2_ solutions with different initial concentrations. Reproduced with permission.^[^ ^335^ ^]^ Copyright 2019, SAGE. d) The *E*–pH diagram for chromium speciation predominance. e) Solvation corrected binding energies calculated by DFT between redox‐active ferrocenium and oxyanions, (inset is the electronic structure optimization of CrO_4_^2−^ with ferrocenium) and f) removal efficiency of chromium and arsenic in different water matrices, involving a municipal secondary wastewater spiked with Cr and As (100 ppb) for 120 min of charging. Reproduced with permission.^[^ ^142^ ^]^ Copyright 2018, Springer Nature. g) Schematic diagram of the electrochemical regeneration cycle of Cu^2+^‐intercalated Bibirnessite electrode. Reproduced with permission.^[^ ^337^ ^]^ Copyright 2017, Springer Nature. h) Schematic diagram of nitrate insertion and reduction by Pd/NiAl‐MMO electrode. Reproduced with permission.^[^ ^63^ ^]^ Copyright 2018, Elsevier.

In addition, an exciting results have been obtained by Wang et al.,^[^ ^334^ ^]^ who designed a low‐vacancy CuHCF electrode to achieve reversible insertion of a wide variety of heavy metal ions as shown in **Figure** **30**a,b, including many monovalent, divalent, and even trivalent ions (such as Rb^+^, Co^2+^, Ni^2+^, Nd^3+^, and Ce^3+^) in aqueous solution. A polypyrrole–DBS/chitosan composite was prepared by chemical polymerization and used as a CDI electrode for heavy metal ion removal.^[^ ^335^ ^]^ As can be seen in Figure 30c, the removal capacity for Cu^2+^ removal increased with the increase of initial concentration of CuCl_2_ solution and reached a high removal capacity of 99.67 mg g^−1^, and the removal performance for other heavy metal ions, such as Cd^2+^, Pb^2+^, and Ag^+^, was also good.

It is notable that the prevalent heavy metals chromium and arsenic are always present in their oxyanions (e.g., Cr_2_O_7_^2−^, CrO4^2−^, HCrO4^−^, H_2_AsO_4_^−^, HAsO_4_^2−^) rather than cations as displayed in Figure 30d. Su et al.^[^ ^142^ ^]^ reported a redox‐active metallopolymer composite electrode for the selective electrochemical removal of chromium and arsenic. The calculated binding energies implies decent charge transfers between the ferrocenium and oxyanions as shown in Figure 30e,f, resulting in high removal efficiency of chromium and arsenic in 20 × 10^−3^ m NaCl solution and real wastewater.

In summary, various materials have been demonstrated as versatile electrode materials to strongly interact with heavy metal cations or oxyanions. However, the same type of ion capture material may require different ion capture mechanisms when capturing different ions. For example, the capture process of Cu^2+^ by MnO_2_ is quite unique, especially when coupled with Bi_2_O_3_ as an additive.^[^ ^337^ ^]^ As shown in Figure 30g, the host MnO_2_ was reduced to Mn(OH)_2_ while the intercalant Cu^2+^ was reduced to metallic Cu in the discharge process, which was quite different from typical Faradaic processes via insertion reactions between cations and MnO_2_ hosts. Moreover, actual wastewater contains a variety of cations and anions with shifting pH, making the removal process more complicated and difficult.

#### 4.2.4 Biological Nutrient Removal

Apart from heavy metal contaminants, agricultural and industrial wastewater also contains large amounts of biological nutrients, such as phosphates and nitrates, causing the deterioration of water quality and serious damage to aquatic ecosystems.^[^ ^338^ ^]^ Zhu et al.^[^ ^339^ ^]^ employed a Mg‐Al LDHs/AC electrode to remove phosphate, showing a high phosphate removal capacity (80.43 mg PO_4_^3−^ per g of electrode). Furthermore, a Pd/NiAl‐MMO composite^[^ ^63^ ^]^ was developed as a novel CDI electrode for nitrate removal. As shown in Figure 30h, this Pd/NiAl‐MMO electrode effectively captured nitrate with concomitant reconstruction of the original hydrotalcite structure by the insertion of NO_3_^−^, and then converted nitrate to nitrogen (N_2_) during the regeneration period.

Despite of much researches for all three selected applications of water softening, heavy metal removal, and biological nutrient removal, CDI with Faradaic electrode for contaminant removal is still in its infancy. More effort is required to accommodate complicated situations and issues, such as multifarious ions with different variants, shifting pH values of wastewater, and the possible chemical instability (e.g., dissolution) of electrodes under complex solution systems.

## 5 Summary and Perspectives

### 5.1 Summary

Among the various desalination technologies, the low cost, high energy‐efficient and eco‐friendly CDI gains rapid advancement in the electrode materials and configurations in recent years. Specifically, compared with conventional carbon electrode‐based CDI which suffers from the expulsion of co‐ions, CDI with Faradaic electrode is much more competitive in the desalination market as it can treat higher salinity streams with enhanced salt removal capacity. Based on studies to date, this paper puts forth a comprehensive overview of the state‐of‐the‐art developments in Faradaic electrode materials for CDI. Specifically, we discussed fundamentals involving CDI cell architectures, key evaluation metrics and operational factors that affect CDI performance, ion capture mechanisms, and the basic requirements for high performance Faradaic electrode materials. Three categories of emerging Faradaic electrode materials for Na^+^, Cl^−^ and Na^+^&Cl^−^ capture were summarized respectively, from the aspects of crystal structures, physicochemical characteristics, ion capture mechanism and desalination performance. In addition, some tailored applications made possible by the Faradaic electrode‐based CDI system were also introduced, which includes selective ion removal and contaminant removal.

### 5.2 Challenges and Perspectives

There has been significant progress in the development of CDI cells with Faradaic electrode. However, it seems that its technical maturity is not yet sufficient to meet the requirements for practical implementation or commercialization, and several major challenges remain in several critical areas ranging from micro‐scale materials to the macro‐scale CDI system, such as not fully understanding the ion capture mechanisms, issue of matching the Na^+^ capture cathode with the Cl^−^ capture anode, undesirable desalination performance including slow salt removal and short cycle life, and the need to improve CDI cell architectures with Faradaic electrodes, etc. More details about these challenges and corresponding perspectives are discussed below.

1) Further fundamental understanding of ion capture mechanisms and material behaviors of Faradaic electrodes. Improved fundamental understandings of ion capture mechanisms and material behaviors can guide new material development and further promote the progress of CDI cells. However, current ion capture mechanisms in Faradaic electrodes are still not fully understood or even controversial, such as uncertainty of the specific ion storage site in electrodes (i.e., precise location of a lattice site or a free space within certain spatial range), or the specific ion form captured by electrode materials (bare ions, hydrated ions, or both and the preference). Moreover, the main factors that influence selective ion removal lack multilevel and systematic exploration. Many factors, including the characteristics of electrode materials, properties of ions, and the interaction between electrodes and ions need to be clarified in various feedwater conditions.

To enhance our ability to study these mechanisms further, it is imperative to employ advanced characterizations, especially in situ techniques, such as in situ scanning/transmission electron microscopy (S/TEM), X‐ray diffraction (XRD), atomic force microscopy (AFM), nuclear magnetic resonance (NMR), and Raman spectroscopy for tracking the crystal structural evolution of the electrodes and the electrochemical behaviors during desalination/salination process at the atomic scale. To gain deeper insights into the detailed mechanism, multiscale modeling and simulation such as DFT‐based first principles methods are necessary to predict relevant properties of electrode materials such as structure stability and electronic property, reveal the interaction between electrode materials and ions, and assist in situ techniques to correlate microscopic characteristics with electrochemical performance, thus achieving rational designs of electrode materials for high‐performance CDI and selective ion removal.

2) Further in‐depth study on the matching issue between the Na^+^ capture cathode and Cl^−^ capture anode. To be specific, due to the difference in material characteristics and ion capture mechanisms between the Na^+^ capture cathode and Cl^−^ capture anode, unequal amount of electrons may be required to capture the same amount of Na^+^ or Cl^−^,^[^ ^43^, ^47^ ^]^ resulting in an imbalance removal of Cl^−^ and Na^+^ from solution. And these two different ion capture processes may present unsynchronized kinetic properties, leading to unoptimized desalination efficiency. Hence, in most cases, one of the two electrodes is rate‐limiting and may dictate the entire CDI performance. Also of note is that, strictly speaking, the imbalance between Na^+^ and Cl^−^ removal may also exist even in conventional CDI with two identical carbon electrodes. The main reason is due to the different properties of Na^+^ and Cl ions in salt solution, which includes the size and wettability of the ions in aqueous solution.

Yet most of the current studies only focus on the desalination performance of whole CDI cell but ignore the balance between the Na^+^ capture cathode and Cl^−^ capture anode. We strongly recommend that future studies investigate the ion capture behavior of the two electrodes separately, in order to facilitate subsequent studies on the matching issue. In addition, choosing cathode and anode materials that require equal ratios of electron transfer in one cell set, and development of the electrode material with 3D open foam structure, nano particles, or highly conductive carbon combination are beneficial to achieve optimized matching between both electrodes and thus maximize the CDI cell desalination performance.

3) More extensive research studies and developments on materials of Cl^−^ capture Faradaic electrode. Since the introduction of the first Faradaic CDI in 2012,^[^ ^39^ ^]^ only very few materials, such as Ag,^[^ ^39^ ^]^ Bi,^[^ ^43^ ^]^ and calcined LDH,^[^ ^57^ ^]^ have been reported as viable Cl^−^ capture Faradaic electrodes.

Given to the similarity among electrochemical technologies, the rapid development of chloride ion batteries may provide inspiration for Cl^−^ storage in CDI systems. In such batteries, BiOCl and other metal oxychlorides, such as FeOCl, TiOCl, VOCl, VOCl_2_, and SnOCl_2_, have been reported as cathode materials and performed reversible reactions with good performance,^[^ ^237^, ^238^, ^242^, ^340^ ^]^ but have not yet been implemented toward Cl^−^ capture in desalination cells. Additionally, the development of Faradaic electrode materials that can capture both Na^+^ and Cl^−^ is also a viable direction. For example, modification of layered 2D materials, such as MXenes and TMDs, is expected to change their ion preference from cations (eg. Na^+^) to anions (eg. Cl^−^). Moreover, it is attractive to further investigate the anion‐exchange capacities of polymer materials and their redox active moieties (such as functional groups, electronic dopants and metallocenes, etc.) that can interact with anions for the further development of available Cl^−^ capture electrodes.

4) Further improvement of the cycle life of the CDI cell with Faradaic electrodes. Currently, most CDI studies with Faradaic electrodes have been reported with a low cycle number within 100 cycles,^[^ ^47^, ^53^, ^55^, ^58^, ^77^, ^94^, ^96^ ^]^ which is not suitable for practical applications in terms of the cost and enlargement. Tracing the reasons, large volume changes and electrode dissolution have significant impacts on the stability of Faradaic electrode. Large volume change commonly occurs in Faradaic electrode materials during prolonged charge/discharge processes, and is especially serious in conversion reaction‐type electrodes (Ag/AgCl and Bi/BiOCl) with more than 150% volume expansion.^[^ ^43^, ^237^ ^]^ Electrode dissolution in aqueous solution will limit the long‐term cyclability and cause secondary pollution to the outlet water. The dissolution of electrode materials in aqueous solution has been noted in previous electrochemical reports,^[^ ^42^, ^165^, ^169^, ^341^, ^342^, ^343^ ^]^ such as disproportionation of Mn^3+^‐containing manganese oxides to Mn^2+^ and Mn^4+^,^[^ ^165^, ^169^ ^]^ the general instability of metal hexacyanometalates at pH 7,^[^ ^342^ ^]^ and the high solubility of silver of 8.9 ppm in 600 × 10^−3^ m NaCl solution.^[^ ^42^ ^]^ Nevertheless, due to the CDI being in its infancy stage, current studies are still focused on the feasibility of various emerging Faradiac electrode materials but lack effective strategies to address the aforementioned issues.

Many strategies implemented for the energy storage systems (e.g., aqueous metal‐ion batteries,^[^ ^344^, ^345^, ^346^ ^]^ chloride ion batteries,^[^ ^238^ ^]^ supercapacitors^[^ ^347^ ^]^) to improve the stability of Faradaic electrodes will be instructive for CDI electrodes. For example, structural design of electrode material by particle nanostructuring, morphology optimization, or constructing composites with other materials can help accommodate the large volume changes during the ion capture/release processes; and strategies like surface protection using coatings or adding surface stabilizers^[^ ^341^, ^348^, ^349^ ^]^ can help alleviate the issue of electrode dissolution, thus improving the cycle life. In addition, some advanced material engineering and functionalization approaches like changing the crystalline phase,^278^ defect engineering,^279^ and element doping^280^ have been demonstrated to be effective to improve electrochemical performance, which should also be tried for the design and optimization of desalination electrode materials to improve long‐term desalination/salination stability.

5) Establishment of uniform standards of testing procedures and operational parameters for CDI performance evaluation. Although we compared the CDI desalination performances consisting of different electrode materials in Section 3, we should caution against over interpreting the performance comparisons because the performance of various CDI devices is strongly linked to electrode and CDI system design parameters rather than just materials sets per se. These include the electrode engineering (such as packing density, mass loading, thickness, and the type of conductive additive), cell engineering (such as cell architectures, with/without IEMs, the balance between cathode and anode), and operational parameters (such as SP/BM experiment approach, CV/CD mode, zero‐voltage/reversed‐voltage regeneration method, initial salt concentration, charge time, and flow rate). These extrinsic parameters are distinct from intrinsic material properties limiting performance such as the volume expansion of a given material upon the Na^+^ or Cl^−^ capture process. Yet we feel that at this point there has been very little research.

Therefore, it is important to draw a set of standard testing procedures and parameters to ensure a fair comparison of desalination performances of CDI cells based on materials sets, and allow for a possible further comparison of this CDI technology with other traditional desalination technologies. One case in point is the initial salt concentration, a uniform level/range of this operational parameter is required for performance comparisons among various CDI cells. And we strongly recommend that this parameter for Faradaic electrode‐based CDI cells should be aimed at highly saline input streams, such as seawater (35 g L^−1^ TDS) or at least brackish water (>1 g L^−1^ TDS), which can fulfill and further develop the potential of Faradaic electrode materials.

6) Further improvements of CDI cell architectures with Faradaic electrodes. Although Faradaic CDI, hybrid CDI, NID and CDI architecture with redox electrolyte have been widely adopted in recent studies and have demonstrated high promise, their respective issues are conspicuous, including little available Cl^−^ capture Faradaic electrodes, the restriction with the low ion removal capacity of the carbon electrode on the whole CDI performance, relatively complex configuration and operation, and the leaching of redox‐active ions into effluent stream. Therefore, more novel architectures and innovative approaches are highly needed to make the CDI technology more practical and competitive.

One direction is to develop the novel architectures used for CDI cell. For example, the very recent advent of carbon‐based flow‐electrode CDI^[^ ^19^, ^350^ ^]^ may provide a new inspiration for the exploitation of Faradaic flow electrode CDI. In carbon‐based flow electrode CDI, both electrodes are composed of a flowing carbon slurry, performing a continuous process without the need for in‐cell electrode regeneration. As for the design of Faradaic flow‐electrode CDI, some essential questions, such as the design of feasible flow‐type electrode, the approach to regenerating Faradaic electrodes, and the actual running of the circular system, should be taken into consideration at the same time. Another direction is the advanced integration of CDI systems with other electrochemical devices to achieve practical targeted goals, such as constructing solar‐driven desalination^[^ ^91^, ^92^ ^]^ or electrochromic desalination^[^ ^93^ ^]^) to achieve self‐sustainable or energy‐efficient desalination as mentioned in Section 2.1. Novel cell concepts should be explored. Metal air desalination seems to pave the way for the next generation of CDI systems with remarkable performance improvements. For instance, Srimuk et al.^[^ ^351^ ^]^ and Dai et al.^[^ ^352^ ^]^ recently proposed the concept of metal air desalination and adapted this for zinc‐air desalination, which provided much higher capacity (up to 1300 mg_NaCl_/g_Zn_) than that of any current CDI systems and demonstrated the potential for simultaneous water desalination and energy supply.

Overall, the recent exciting progresses in Faradaic electrode for CDI show its promise for water desalination. Great significance is supposed to be attached to the in‐depth exploration and continuous optimization of novel types of Faradaic electrode materials and CDI systems. Moreover, although there have been relatively few reports overall, other factors, such as the cost, safety, and environmental effects, should also be taken into consideration in order to realize scale‐up from the laboratory. In addition, studies with specific aims will make the Faradaic electrode‐based CDI more suitable for tailored applications. On the basis of recent progresses and further developments expected in the near future, the emerging Faradaic electrode‐based CDI are predicted to play an important role in water desalination‐related applications, which will promote a “greener” future.

## Conflict of Interest

The authors declare no conflict of interest.
